# Derivation and propagation of spermatogonial stem cells from human pluripotent cells

**DOI:** 10.1186/s13287-020-01896-0

**Published:** 2020-09-23

**Authors:** Huiming Xu, Mengbo Yang, Ruhui Tian, Yonghui Wang, Linhong Liu, Zijue Zhu, Shi Yang, Qingqing Yuan, Minghui Niu, Chencheng Yao, Erlei Zhi, Peng Li, Chenhao Zhou, Zuping He, Zheng Li, Wei-Qiang Gao

**Affiliations:** 1 State Key Laboratory of Oncogenes and Related Genes, Renji-MedX Clinical Stem Cell Research Center, Ren Ji Hospital, School of Medicine, Shanghai Jiao Tong University, 160 Pujian Road, Shanghai, 200127 China; 2 School of Biomedical Engineering & Med-X Research Institute, Shanghai Jiao Tong University, Shanghai, China; 3 Department of Andrology, the Center for Men’s Health, Urologic Medical Center, Shanghai Key Laboratory of Reproductive Medicine, Shanghai General Hospital, Shanghai Jiao Tong University School of Medicine, 100 Haining Road, Shanghai, 200080 China; 4 Department of Urology, Shanghai Human Sperm Bank, Shanghai Institute of Andrology, Renji Hospital, School of Medicine, Shanghai Jiao Tong University, Shanghai, 200135 China

**Keywords:** Human pluripotent stem cells, Differentiation, Human spermatogonia stem cells, Xeno-transplantation

## Abstract

**Objectives:**

This study is designed to generate and propagate human spermatogonial stem cells (SSCs) derived from human pluripotent stem cells (hPSCs).

**Methods:**

hPSCs were differentiated into SSC-like cells (SSCLCs) by a three-step strategy. The biological characteristics of SSCLCs were detected by immunostaining with antibodies against SSC markers. The ability of self-renewal was measured by propagating for a long time and still maintaining SSCs morphological property. The differentiation potential of SSCLCs was determined by the generation of spermatocytes and haploid cells, which were identified by immunostaining and flow cytometry. The transcriptome analysis of SSCLCs was performed by RNA sequencing. The biological function of SSCLCs was assessed by xeno-transplantation into busulfan-treated mouse testes.

**Results:**

SSCLCs were efficiently generated by a 3-step strategy. The SSCLCs displayed a grape-like morphology and expressed SSC markers. Moreover, SSCLCs could be propagated for approximately 4 months and still maintained their morphological properties. Furthermore, SSCLCs could differentiate into spermatocytes and haploid cells. In addition, SSCLCs displayed a similar gene expression pattern as human GPR125^+^ spermatogonia derived from human testicular tissues. And more, SSCLCs could survive and home at the base membrane of seminiferous tubules.

**Conclusion:**

SSCLCs were successfully derived from hPSCs and propagated for a long time. The SSCLCs resembled their counterpart human GPR125^+^ spermatogonia, as evidenced by the grape-like morphology, transcriptome, homing, and functional characteristics. Therefore, hPSC-derived SSCLCs may provide a reliable cell source for studying human SSCs biological properties, disease modeling, and drug toxicity screening.

## Introduction

Spermatogenesis is a complex and well-regulated biological process in which spermatogonial stem cells (SSCs) are the only germ cells that can self-renew and form spermatozoa. Any error during the process can result in infertility, a major health problem for men [1]. Therefore, studying human SSCs is of great importance for reproductive science and finding new treatments for male infertility. SSCs only account for 0.03% of total germ cells in the adult male mouse [2]. SSC self-renewal keeps the stability of the stem cell pool, while their differentiation gives rise to germ cells including haploid spermatids [3]. In humans, it is generally believed that there are three types of spermatogonia. Both Adark and Apale spermatogonia are considered stem cells. Adark spermatogonia function as reserve stem cells, while Apale spermatogonia are self-renewing stem cells and give rise to type B spermatogonia that further divide into spermatocytes to produce spermatids [4–6]. By now, transplantation is the only method of testing the function of SSCs. SSCs are transplanted into the lumen of seminiferous tubules and are induced into complete spermatogenesis and produce offspring [7, 8]. However, it is only applicable for animals, and difficult for human being. By now, the vast knowledge about SSCs is based on mouse models. Therefore, in this study, we focus on human SSCs. Our understanding of biological properties and embryonic development of human SSCs is limited, because human primary SSCs are relatively inaccessible due to ethical and practical problems and their limited proliferation ability [9]. Therefore, an available and reliable cell resource of human SSCs is requisite for basic research and regenerative medicine.

Human pluripotent stem cells (hPSCs) are a promising source for the generation of human SSCs due to their self-renewal ability and multi-lineage differentiation potential [10, 11]. In the past several years, human germ cells including primary germ cells (PGCs) and haploid spermatids have been successfully generated from hPSCs [12–17]. By now, the generation and propagation of human SSCs derived hPSCs is rarely reported. Although a recent work reports that spermatogonium-like cells can be produced from hPSCs by a one-step approach under feeder-free conditions, and that the spermatogonium-like cells express spermatogonial marker proteins and germ cell specific genes [18], the phenotype, differentiation efficiency, and expansion ability of these spermatogonium-like cells have not been determined.

In the present study, we report a stepwise approach that not only allows efficient generation of spermatogonial stem cells like cells (SSCLCs) from hPSCs in vitro, but also finds that SSCLCs could be propagated for a long time and differentiated into spermatocytes and haploid cells. Therefore, our study provides an in vitro method for the production and expansion of SSCLCs to facilitate the biological research of human SSCs.

## Materials and methods

### Testicular biopsy sample collection

Testicular biopsies were collected from obstructive azoospermia (OA) patients. Testicular tissue was collected for human SSC cultures. The diagnosis of OA patients was confirmed by pathological examination of testes via biopsy. This study was approved by the Institutional Ethical Review Committee of Ren Ji Hospital (license number 2012-01), Shanghai Jiao Tong University School of Medicine, and all participants provided written consent.

### Cell culture and generation of SSCLCs from hPSCs

Human ES cell line SHhES2 (named hES2) [19] and human iPS cell line N-iPSC-1, (named hiPS) [20] (gift from Ying Jin) were cultured and propagated as previously described [20]. In the differentiation system, hES2 and hiPSC were dissociated and re-plated onto six well plates precoated hES-qualified Matrigel (Corning) in the TeSR™ Medium (Stem Cell Technologies) for 1 day, then were changed to the α-MEM medium (Life Technologies) supplemented with 0.88 μM stearic acid, 60 μM putrescine, 2.36 μM palmitic acid, 0.21 μM palmitoleic acid, 2.71 μm linoleic acid, 1.02 μM oleic acid, 0.43 μM linolenic acid, 10 μg/ml transferrin, 10 mM Hepes, 2 mM L-glutamine, 50 μM β-mercaptoethanol, 0.2% BSA, 5 μg/ml insulin, 20 ng/ml GDNF (R&D systems), 1 ng/ml bFGF (Peprotech), and 0.5× penicillin/streptomycin for 10 days [14]. In addition, the cultures were maintained at 37 °C for first 6 days and then were kept at 34 °C for the following dyas. Then, the culture medium was changed to DMEM/F12 medium supplemented with 2% FBS (all from Life Technologies), 5 ng/ml GDNF, 2 μM retinoic acid (RA), and 50 ng/ml stem cell factor (SCF, Humanzyme). Lastly, the differentiated cells were allowed to grow in advanced DMEM/F12 complemented with 4 ng/ml GDNF, 0.1 μg/ml testosterone, 8 μg/ml vitamin C (VC), 3 U/ml vitamin A (VA), 0.2 μg/ml vitamin E (VE), and 0.1 U/ml recombinant human FSH (rhFSH). The above reagents were all from Sigma-Aldrich, unless otherwise stated.

### Maintenance of hPSC-generated SSCLCs

To maintain and expand the above hPSC-generated SSCLCs, the SSCLCs were dissociated by collagenase IV, and then re-plated onto combinational poly-lysine- (0.05 mg/ml, Sigma-Aldrich) and laminin-coated dishes (dishes were coated with 0.1 mg/ml laminin pre-coated with poly-lysine) in human SSC (hSSC) medium containing the StemPro-34 SFM medium with 1× B27, 1% FBS, 0.5% BSA, 6 g/l D-glucose, 10 μg/ml biotin, 10^− 4^ M VC, 1× sodium pyruvate, 2 mM glutamine, 50 μM β-mercaptoethanol, 1% Vitamine, 1× non-essential amino acid, 1% D.L-lactate (Sigma-Aldrich), 15 ng/ml GDNF, 10 ng/ml bFGF (Peprotech), 20 ng/ml EGF (Peprotech). The above reagents were all from Life Technologies, unless otherwise stated.

### Differentiation of SSCLCs

To differentiate SSCLCs into haploid cells, SSCLCs P2 were digested with by collagenase IV, and then re-plated onto combinational poly-lysine- and laminin-coated dishes in above human SSC medium for 2 days, then changed to DMEM/F12 medium with 1% knockout serum replacement (Life Technologies), 2% lipid rich BSA (Life Technologies), 5 ng/ml GDNF, 50 μM VC, 2 μM RA, 100 ng/ml SCF, and 0.1 μM testosterone for 10–20 days; the medium was changed every 2 days.

### Meiotic spread assays

Meiotic spread assays were performed to determine the meiotic progression in the differentiated cells derived from SSCLCs at P2 according to the method as described previously [15]. The cells were then incubated with primary antibodies rabbit anti-SCP3 (1:100, Abcam), mouse anti-MLH1 (1:50, BD), and human anti-CREST (1:100, ImmunoVision) overnight at 4 °C. The corresponding conjugated secondary antibodies, Alexa Fluor 488 and Alexa Fluor 594 (Life Technologies) at 400-fold dilution in 3% BSA were applied and incubated for 90 min at 37 °C. Lastly, the cells were washed three times with PBS, and the images were captured with an inverted fluorescence microscope.

### Flow cytometry and isolation of 1N haploid cells

The differentiated cells were dissociated with trypsin and washed with cold PBS. They were separately stained with IgG or APC-conjugated SSEA1 or PE-conjugated C-kit antibody (Biolegend) in PBS containing 0.1% FBS at 4 °C for 45 min. Upon completion of washing with PBS, the labeled cells were re-suspended and at least 10^5^ events were acquired by using BD Accuri C6 Flow Cytometer and analyzed using the software FlowJo.

Flow cytometry was performed to measure DNA content of SSCLC-differentiated cells. In brief, after washing with PBS, cells were stained with PBS containing 25 μg/ml propidium iodide (PI) (Sigma-Aldrich), 40 μg/ml RNase (Life Technologies), and 0.3% Tween-20 at room temperature for 20 min; then, the cells were analyzed with a BD FACS Calibur system. To isolate 1N haploid cells, cells were stained with 10 μg/ml Hoechst 33342 (Sigma-Aldrich) in culture medium at 34 °C for 60 min. The haploid 1N peak was collected and fixed with 4% PFA before immunostaining.

### Fluorescence in situ hybridization (FISH)

FISH assay was performed to determine ploidy of the sorted cells using centromeric FISH probes against X, Y, and 18 [14]. Cells were dehydrated with series of graded alcohols; the specimens were further prepared using ZytoLight® FISH-Cytology Implementation Kit (ZytoVision) according to the manufacturer’s instructions. Human chromosomes X, Y, and 18 were detected using ZytoLight® SPEC 18/CEN X/Y Triple Color Probe (ZytoVision). DAPI was used to label cell nuclei. The images were visualized by using an inverted fluorescence microscope or ZEISS confocal microscope.

### H&E and immunofluorescence assay of testicular sections

Testicular tissues were fixed in 4% PFA overnight, embedded in paraffin, and sectioned at 5-μm thickness. The sections were stained with hematoxylin and eosin (H&E) and observed under an inverted microscope.

For immunofluorescence staining for paraffin sections, endogenous peroxidase was quenched by incubating testes sections with PBS containing 3% hydrogen peroxide (H_2_O_2_) for 30 min. Subsequently, the sections were blocked in 10% donkey serum for 1 h at RT and then incubated overnight at 4 °C with primary antibodies. After washing with PBS, the sections were incubated for 1 h at room temperature with the corresponding conjugated secondary antibody.

### Immunofluorescence staining

The cells grown on glass slips were fixed with 4% PFA in PBS. After 3 washes with PBS, they were permeabilized in 0.1% Triton X (Sigma) and then blocked with 10% normal donkey serum. Cells were then incubated overnight at 4 °C with the primary antibodies. After washing with PBS for three times, cells were then incubated by the corresponding conjugated secondary antibodies, Alexa Fluor 488, Alexa Fluor 594, and Alexa Fluor 647 at 400-fold dilution in 3% BSA in PBS. Cell nuclei were labeled with DAPI (Sigma). The imagines were visualized by using an inverted fluorescence microscope or ZEISS confocal microscope.

The primary antibodies were shown as follows: PLZF (1:100, mouse, Abcam), UCHL1 (1:200, mouse, AbD Serotec), CD90 (1:100, rabbit, Abcam), GPR125 (1:100, rabbit, Abcam), GFRΑ1 (1:150, rabbit, Sigma), GFRA1 (1:50, mouse, Santa Cruz), MAGEA (1:100, rabbit, Abcam), SCP3 (1:100, rabbit, Abcam), SSEA1 (1:100, mouse, BD), c-kit (1:100, rabbit, Abcam), VASA (DDX4, 1:200, rabbit, Abcam), PCNA (1:100, rabbit, Abcam), OCT4 (1:200, rabbit, a gift from Ying Jin), Nanog (1:100, Cell Signaling Technology), and SOX2 (1:100, rabbit, Abcam).

### hPSCs-derived SSCLCs transplantation

The hPSCs-derived SSCLCs transplantation experiment was performed as described previously [21]. In brief, male nude mice of 6–8 weeks old were treated with busulfan (Sigma) at 40 mg/ kg body weight to deplete male germ cells in the testes; 1 month later, the recipient mice were transplanted with 20 × 10^6^ cells/ml hPSC-derived SSCLCs at passage 2 (P2) or P3 enriched by magnetic-activated cell sorting (MACS, Mitenyi Biotech) against GPR125 antibody in PBS containing 0.04% (w/v) trypan. Approximately 15 μl SSCLCs were transplanted into the seminiferous tubules of one testis via the efferent duct, and the testis without cell transplantation served as an internal control. Five weeks after transplantation, the testes of the recipient mice were collected for preparing frozen sections and paraffin sections.

### Quantitative real-time PCR

RNA was extracted by using the Direct-Zol™ RNA Mini kit (ZYMO RESEARCH) and reversely transcribed to cDNA using PrimedScript™ RT Master Mix (Takara). The mRNA levels were quantified by SYBR Green-based quantitative real-time PCR (Takara) using an ABI Prism 7900 HT (Applied Biosystems). Results were confirmed in at least three separate analyses. The sequences for gene primers are shown in Table S1.

### RNA sequencing

hPSCs and SSCLCs at P1, P7 which were enriched by MACS using GPR125 antibody were lysed in RNA lysate buffer and total RNA were extracted and were amplified with SMART-Seq™ v4 Ultra™ Low Input RNA Kit (Clontech). GPR125^+^ cells isolated by MACS derived from three OA patients were lysed in RNA lysate buffer and were amplified with above RNA kit. RNA library was constructed using NuGen Ovation Ultralow System (NuGen) following the instruction of the manufacture. Agilent 2100 Bioanalyzer and Qubit® 2.0 Fluorometer were used to qualify and quantify the sample library. The qualified RNA samples were subsequently sequenced using the Illumina Hiseq-2500 platform with a 2 × 50 bp modality. Raw sequencing reads were mapped to the human reference genome hg38 using Hisat2 (version 2.0.4) [22]. FPKM (fragments per kilobase of exon model per million mapped reads) was computed using Stringtie (version:1.3.0) and normalized with TMM [23–25].

### Isolation and culture of human SSCs

The biopsies of Human GPR125-positive spermatogonia were separated using procedures as previously described with minor modification [26]. In brief, human testicular cells were isolated from human testis biopsies of OA patients using two-step enzymatic digestion. Because of differential plating, cells were seeded into culture plates in DMEM/F-12 (Gibco) supplemented with 10% FBS (Hyclone) for 12 h. After incubation, other cells attached to the culture plates, whereas male germ cells remained in suspension and collected by centrifugation at 1000 rpm for 5 min. hSSCs were enriched by MACS using antibody against GPR125 according to the instruction of manufacture (Mitenyi Biotec). Then, the enriched cells were cultured in hSSC medium with the supplement of human LIF (Millipore) to form colonies.

### Statistical analysis

Data derived from at least three independent experiments were presented as mean ± SEM. The relative mRNA levels were normalized to GAPDH expression. Statistical significances were tested by Student’s *t* test, and *p* values ≤ 0.05 were considered statistically significant.

## Results

### Generation of SSCLCs from hPSCs by a three-step strategy

**Fig. 1:**
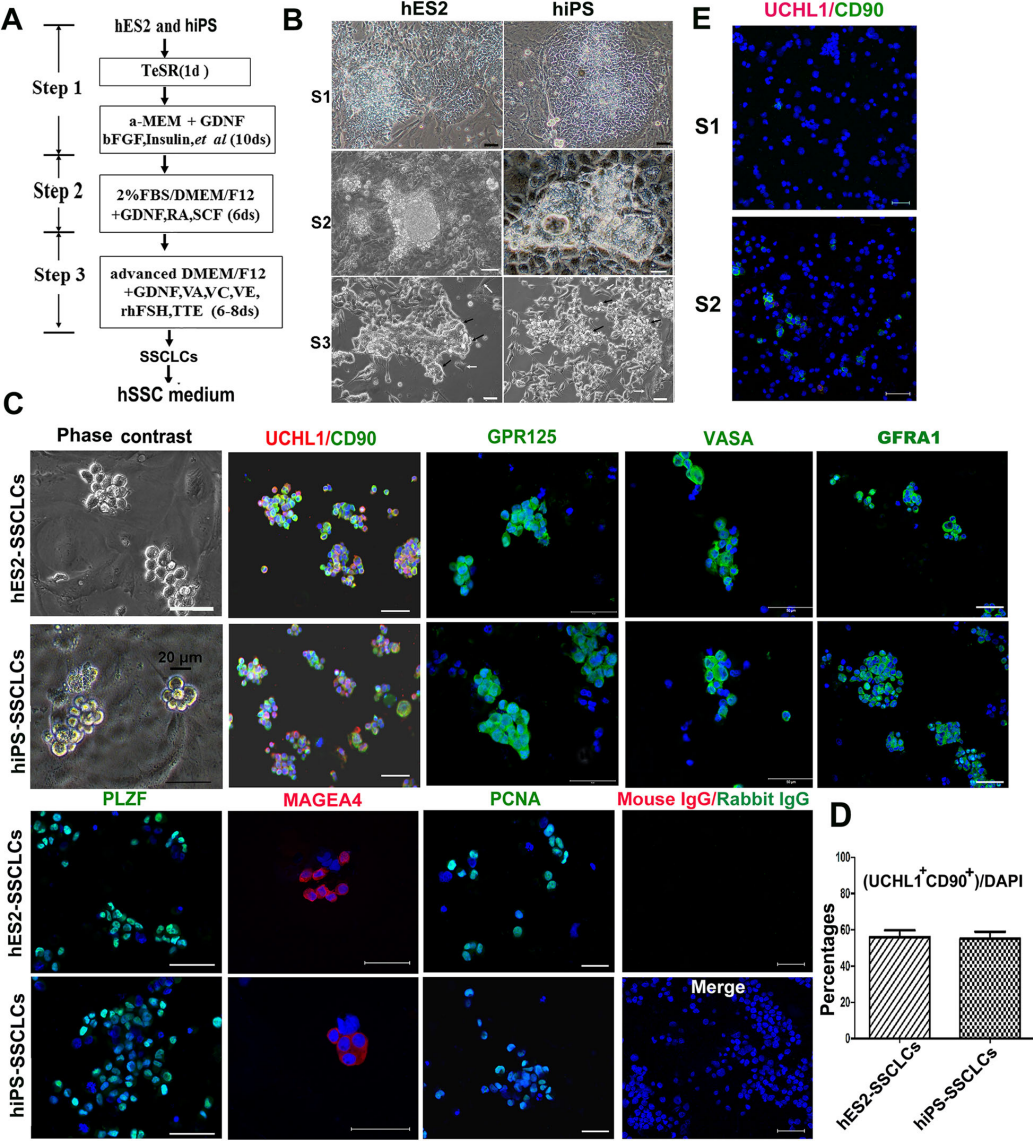
Differentiation of hES2 and hiPS into SSCLCs by a three-step approach. **a** Schematic illustration of a three-step protocol to differentiate hES2 and hiPS into SSCLCs. TTE, testosterone; RA, retinoic acid. **b** Cell morphology of differentiated cells derived from hES2 and hiPS at stage 1–stage 3 (S1-S3). Scale bars, 50 μm. Black arrows indicate the cells with grape-like phenotype. Bright arrows indicate the cells with fibroblastic phenotype. **c** Phase-contrast images and immunostaining images of the differentiated cells at stage 3 with SSC marker antibodies against UCHL1, CD90, GPR125, VASA, GFRA1, PLZF, MAGEA4, and PCNA. Mouse IgG/corresponding Alexa Fluor 594, and Rabbit IgG/corresponding Alexa Fluor 488 were used as negative control. Scale bars, 50 μm. **d** Quantification of the percentages of UCHL1 and CD90 double-positive cells over the total cells. **e** Immunostaining images of the differentiated cells derived from hiPS at stage 1 and stage 2 with the antibodies against UCHL1 and CD90. Scale bars, 50 μm

Over the last decade, much effort has been taken to obtain PGCs and haploid spermatids from hPSCs using a one-step strategy by adding various growth factors and compounds to the differentiation medium [12–17]. In the current study, we decided to induce the generation of spermatogonia by a stepwise approach according to the development process of spermatogonia, Fig 1a is a diagram of our 3-step induction approach. At the first stage, hESC line (SHhES2, named hES2) and hiPSC line (N-iPSC-1, named hiPS) were cultured in α-MEM medium containing insulin, transferrin, putrescine, bFGF, GDNF, and several fatty acids that include stearic acid, palmitic acid, palmitoleic acid, linoleic acid, oleic acid, and linolenic acid for 10 days, which appeared to facilitate the production of primordial germ cells (PGCs) [12]. To testify if PGCs were generated from hES2 or hiPS, we performed immunostaining with the differentiated cells at stage 1with PGC markers. Fig. S1a and b shows that the expression levels of STELLA, a PGC marker [26], were increased and most differentiated cells at stage 1 were positive for SSEA1, c-kit, and VASA, markers for human PGCs [26, 27]. In contrast, hES2 were negative for SSEA1, c-kit, and VASA (Fig. S1c). Based on the data, some PGC-like cells (PGCLCs) could be generated at stage 1. At the second stage, the medium was changed to DMEM/F12 medium containing RA and SCF for 6 days, which are believed to promote the proliferation of PGCs [28, 29]. In this way, the produced PGCLCs were able to form cell clusters (Fig. 1b), in which the identity of PGCLCs was confirmed by immunostaining with SSEA1 and OCT4 antibodies, which are co-expressed by human PGCs [27] (Fig. S1d). At the last stage, the cultures grew in the advanced DMEM/F12 medium supplemented with GDNF, various vitamins, and hormones including VC, VA, VE, testosterone, and rhFSH for 6 days, which provide a testicular niche to promote PGCLCs to differentiate into spermatogonia. Under these culture conditions, the vast majority of differentiated cells exhibited a grape-like SSC morphology, while a small number of cells displayed a fibroblast-like shape and the cells were named FBs (Fig. 1b). It was noted that spermatocyte and haploid spermatids could also be generated from hES2 and hiPS during the induction of SSCs, which was verified by the expression of SCP3 (a spermatocyte marker) and TNP2 (a haploid cell marker) based on real-time PCR (Fig. S1a). In addition, hPSC-differentiated cells after three-step induction were negative for Nanog (a pluripotent cell marker); moreover, no teratomas were formed after the cells were transplanted into testes (data not shown), indicating that there were almost no pluripotent cells remaining in the culture system after three-step induction (Fig. S1e).

To verify the biological characteristics of the SSC-like cells (SSCLCs) after the 3-step induction, we dissociated the differentiated cells and re-plated them onto poly-lysine- and laminin-co-coated dishes, a method to enhance the attachment and survival of SSCs [30], in the StemPro-34 SFM medium supplemented with bFGF, GDNF, and hLif, they could form grape-like colonies (Fig. 1c). At the same time, the dissociated cells were immunostained for spermatogonia markers. As there is not a specific marker for human SSCs, a panel of spermatogonia markers which include VASA, CD90, UCHL1, GFRΑ1, PLZF, GPR125, and MAGEA4 are used for the identification of human SSCs [3, 31, 32]. Figure 1c shows that the majority of the differentiated cells were positive for these spermatogonia markers. We quantified a population of UCHL1 and CD90 double-positive cells, which showed 56.11% ± 6.3% for hES2-derived cells and 55.08 ± 6.8% for hiPS-derived cells (Fig. 1d). In addition, the differentiated cells derived from hiPS at stage 1 and stage 2 showed a low expression level of UCHL1 and CD90 (Fig. 1e), and a similar result was for hES2 (data not shown), indicating that SSCLCs were mainly generated at stage 3. As a contrast, hES2 were negative for the spermatogonia markers and positive for pluripotent markers (Nanog, SOX2, OCT4) (Fig. S1c). In addition, SSCLCs were positive for PCNA (Fig. 1c), a marker reflecting the proliferation of spermatogonia [33], which indicated that SSCLCs were proliferative. Taken together, the above data suggested SSCLCs could be efficiently produced from hES2 and hiPS by the three-step approach.

### Successful propagation of SSCLCs derived from hES2 and hiPS

**Fig. 2:**
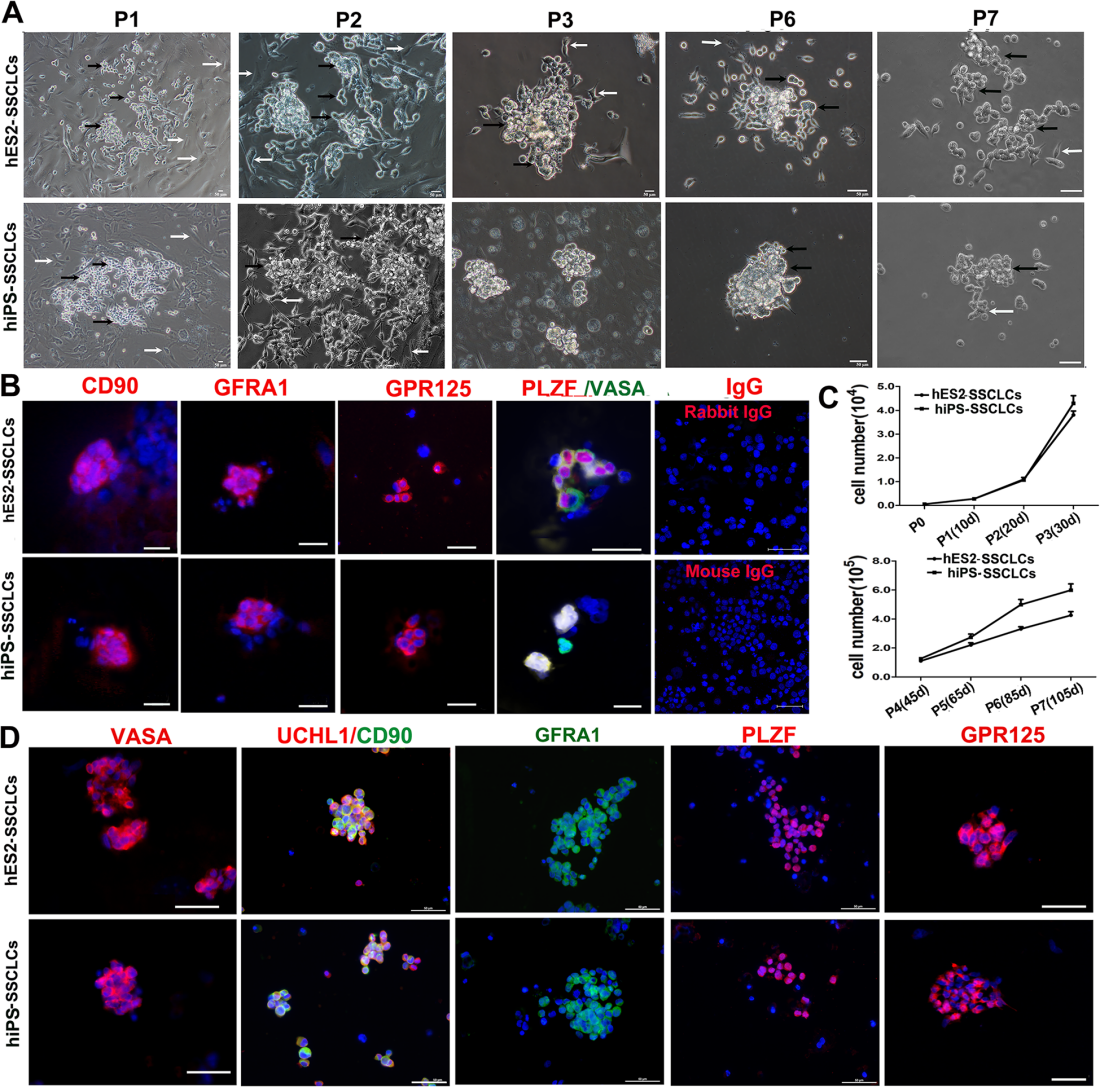
hES2-SSCLCs and hiPS-SSCLCs were maintained in vitro for a long time. **a** Phase-contrast images of the cell morphology of SSCLCs at P1-P7. Black arrows indicate grape-like morphology of SSCLCs. Bright arrows indicate fibroblast-like cells (FBs). Scale bars, 50 μm. **b** Immunostaining images of SSCLCs clusters derived from hES2 and hiPSC at passage 2 with the antibodies against CD90, GFRA1, GPR125, PLZF, and VASA. Rabbit/mouse IgG and corresponding Alexa Fluor 594 were used as negative control. Scale bars, 50 μm. **c** Cell number of VASA and UCHL1 double-positive cells at the indicated time points and passages. Cell counts are mean ± SEM from three independent different experiments. **d** Immunostaining images of SSCLCs at P7 with the antibodies against VASA, UCHL1, CD90, GFRA1, PLZF, and GPR125. Scale bars, 50 μm

SSCs can self-renew to maintain the stability of stem cell pool [3]. To determine if SSCLCs could be propagated and maintained for a long time, we dissociated the hES2- and hiPS-derived SSCLCs after the 3-step induction and re-plated the SSCLCs onto poly-lysine- and laminin-co-coated dishes in the hSSC medium including StemPro-34 SFM medium supplemented with B27, 1% FBS, 0.5% BSA, 6 g/l D-Glucose, 10 μg/ml Biotin, 10^− 4^ M VC, 15 ng/ml GDNF, 10 ng/ml bFGF, 20 ng/ml EGF, and 10 μg/ml hLif. Of note, some hPSC-derived fibroblast-like shape cells (named FBs) still appeared among the SSCLCs (Figs. 1b and 2a). However, SSCLCs at passage 3 (P3) contained very few FBs (Fig. 2a). Interestingly, the SSCLCs could form healthy colonies and gradually expanded (Fig. 2a). To confirm whether the colonies were SSCs or not, we immunoassayed the SSCLCs colonies at P2 for spermatogonia markers; the colonies expressed CD90, GFRA1, GPR125, VASA, and PLZF, while the surrounding cells did not express these spermatogonia markers (Fig. 2b). Strikingly, after sub-culturing them for 30 days (P3), a significant number of SSCLCs proliferated and formed more and larger cell clumps (Fig. 2a, P3), resulting in an approximately 80-fold increase in the number of cells that were double positive for VASA and UCHL1 (Fig. 2c). Subsequently, SSCLCs began to slowly proliferate and could be propagated for up to 7 passages (about 4 months), showing an approximately 900-fold increase in the number of cells that were double positive for VASA and UCHL1 within 115 days (Fig. 2c). However, after 7 passages, SSCLCs formed small colonies and no longer proliferated, similar to human SSC in vitro cultures [34]. To further testify whether SSCLCs could still sustain spermatogonia properties after long-time culture, we immunostained SSCLCs at P7 with spermatogonia markers. Figure 2d shows that SSCLCs at P7 still expressed the spermatogonia markers, VASA, CD90, UCHL1, GFRΑ1, PLZF, and GPR125. Moreover, SSCLCs cultured in hSSC medium did not express spermatocyte and haploid markers (data not shown). Additionally, the SSCLCs could be successfully cryopreserved at P1–P3, with a recovery rate of 70–80% for SSCLCs. Taken together, the SSCLCs derived from hES2 or hiPS could be propagated and maintained for a long time.

### Generation of spermatocytes and haploid cells from SSCLCs

**Fig. 3:**
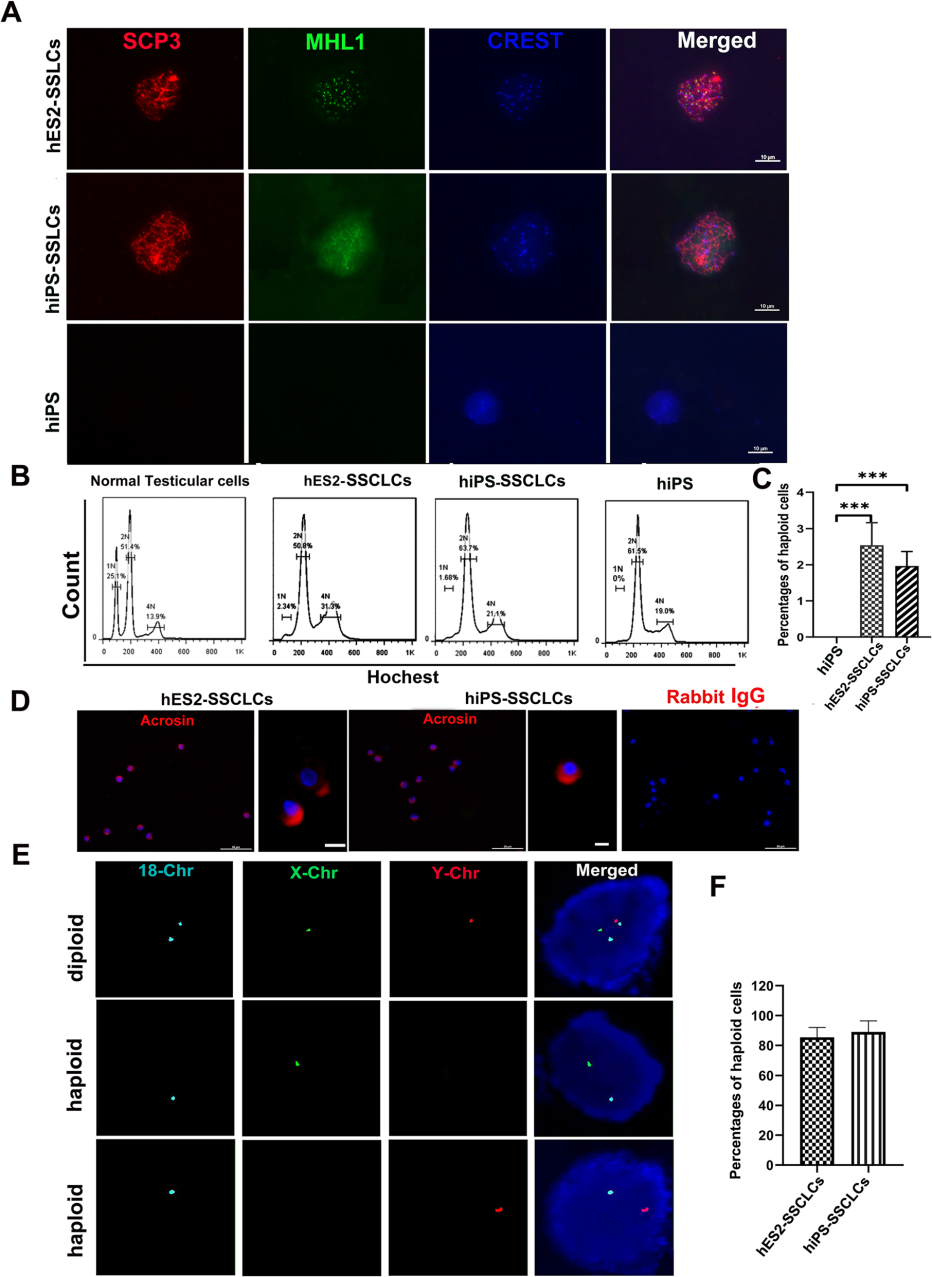
Meiotic procession of SSCLCs and formation of haploid cells. **a** Meiotic spread assays of differentiated cells derived from SSCLCs were performed with the antibodies against SCP3, MHL1, and CREST. hiPS were used as negative control. Scale bars, 10 μm. **b** Haploid cells derived from hES2-SSCLCs and hiPS-SSCLCs at P2 were analyzed by FACS with Hoechst 33342. The human testicular cells derived from OA patients were used as positive control. hiPS were used as negative control. **c** Quantification of the percentages of haploid cells in different group. **d** FACS-isolated haploid cells were stained with acrosin and DAPI. Scale bars, 50 μm in low magnification images. Scale bars, 5 μm in high magnification. Rabbit IgG and corresponding Alexa Fluor 594 were used as negative control. **e** FISH analyses of sex chromosomes and Chr 18 on diploid cells and haploid cells derived from SSCLCs. **f** A quantification of haploid cells of FISH signal of X chr or Y chr. The rate represents the number of haploid cells over total haploid cells isolate by FACS. At least 100 cells were counted per experiment and at least three independent experiments were performed. Data are represented as mean ± SEM

To determine whether SSCLCs could enter meiosis to generate haploid cells, we performed a differentiation experiment. Firstly, SSCLCs at P3 were dissociated and cultured in the hSSC medium for 2 days. Then, the cells were transferred into the differentiation medium containing knockout serum replacement, lipid rich BSA, GDNF, RA, SCF, and testosterone for 15–20 days. Next, we performed meiotic spreading assays using antibodies against SCP3 (a protein locating in the synaptonemal complex), MLH1 (a protein measuring meiotic recombination rate), and CREST (a protein determining centromeric regions) [35] to observe the progression of meiosis including chromosomal synapsis and recombination. Figure 3a shows that the differentiated cells derived from SSCLCs co-expressed SCP3, MHL1, and CREST. In contrast, none of the parental hiPS exhibited SCP3 and MLH1 expression. The results indicated some differentiated cells had entered meiotic process. To further confirm whether haploid cells were generated from SSCLCs, DNA contents of SSCLC-derived cells were analyzed by flow cytometry with Hoechst 33342. As shown in Fig. 3b, a very small haploid peak appeared in the hES2-SSCLC-derived cells and hiPS-SSCLC-derived cells. In the positive control culture, an evident haploid peak was seen in human testicular cells of obstructive azoospermia (OA) patients, while no haploid peak was observed in hiPS. The quantification was performed and the percentages of haploid cells were 2.54% ± 0.62% for hES2-SSCLCs and 1.87 ± 0.35% for hiPS-SSCLCs (Fig. 3c). Furthermore, the haploid cells selected by FACS confirmed their identity by immunostaining with acrosin (a spermatid marker), which exhibited a polar acrosin localization (Fig. 3d). To detect chromosome numbers in selected haploid cells, fluorescence in situ hybridization (FISH) analysis was performed for several chromosomes, including X and Y sex chromosomes (Chr), and Chr 18. We found the sorted haploid cells were positive for either X or Y Chr, and one copy of Chr 18 in the same nuclei (Fig. 3e). Then we quantified the rate of haploid cells with FISH signal for X or Y Chr among the sorted haploid cells is 85.4% ± 6.93% for hES2-SSCLCs and 89.0% ± 7.48% for hiPS-SSCLCs (Fig. 3f). As a control, diploid cells isolated from hiPS displayed both X and Y Chr, as well as two copies of Chr 18 in the same nuclei. Thus, the isolated haploid peak did not contain the cells undergoing apoptosis with fragmented DNA. All together, these results suggested that a small percentage of haploid cells could be generated from SSCLCs.

### Transcriptome analysis of hPSC-derived SSCLCs

**Fig. 4:**
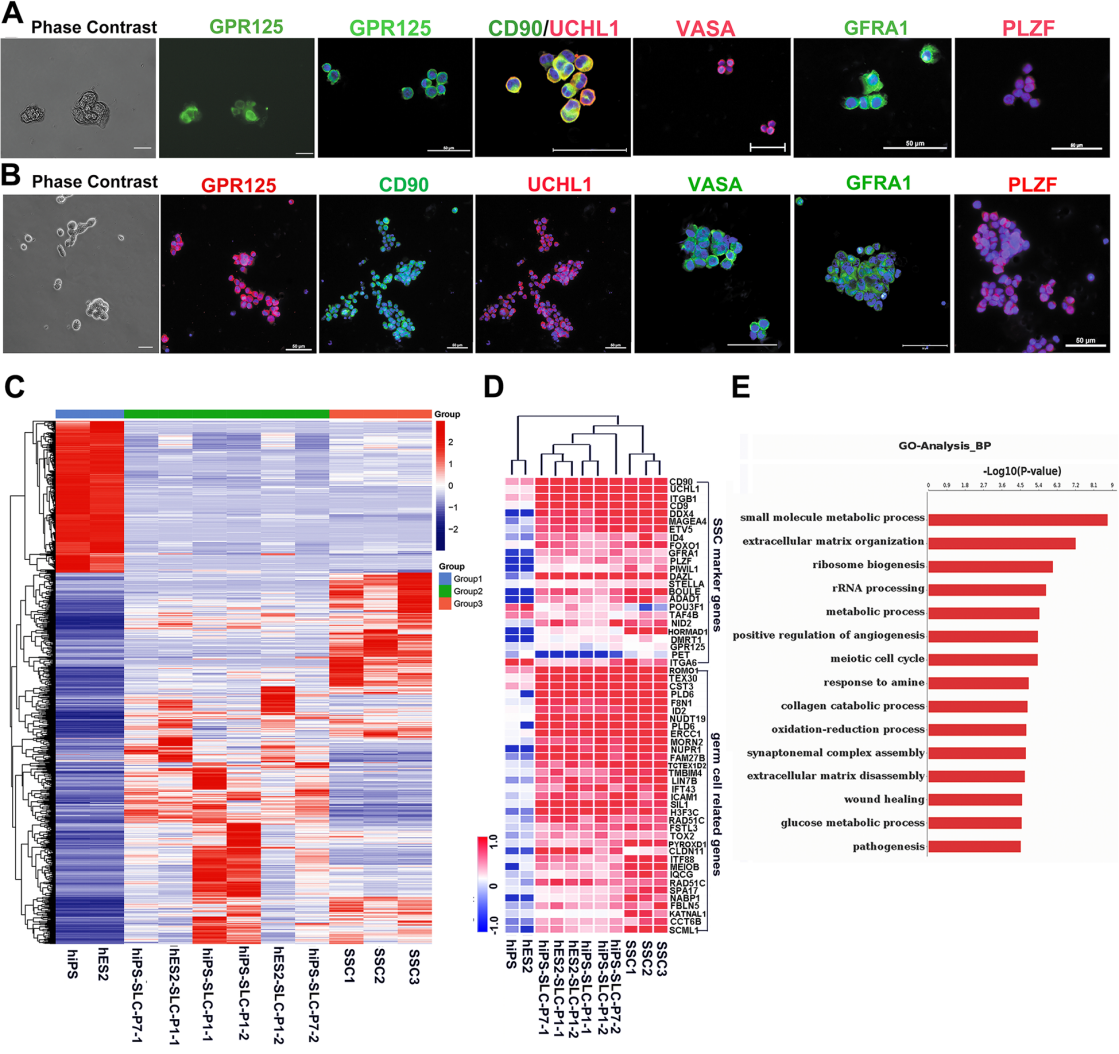
Comparison of the transcriptomes of hPSCs, SSCLCs, and enriched GPR125^+^ cells isolated from human testes. **a, b** Characterization of enriched GPR125^+^ Cells isolated from human testes of OA patients or SSCLCs. Phase contrast and identification of immunoassay of GPR125^+^ cells isolated from human testes of OA patients or hiPS-SSCLCs with antibodies against GPR125, CD90, UCHL1, VASA, GFRA1, and PLZF. **a** Enriched GPR125^+^ cells from human testes of OA patients. **b** hiPS-SSCLCs. Scale bars, 50 μm. **c** Heatmap and hierarchical clustering of significant genes expression of 1042 genes (*P* < 0.001) in SSCLCs at P1 or P7 and enriched GPR125^+^cells isolated from human testes. SLC represents SSCLCs. SSC1, SSC2, and SSC3 represent enriched GPR125^+^cells isolated from three OA patients. Data are represented as mean ± SEM. See also Table S2. **d** Heatmap on transcript expression of SSC markers, germ cell-related genes in hPSCs, SSCLCs, and enriched GPR125+ cells isolated from human testes. See also Table S3. **e** GO biological enrichment analysis of differential genes whose expression changes ≥ 2-fold identified between enriched GPR125^+^ cells and SSCLCs. BP represents biological process. Data are represented as mean ± SEM. Also see Table S2

To determine if SSCLCs have a similar gene expression pattern as their in vivo counterparts, we first isolated spermatogonia from human testicular tissues. Considering that GPR125 has been explored to successfully enrich spermatogonia from human testes [31], we decided to enrich spermatogonia from germ cells of human testes by magnetic-activated cell sorting (MACS) using GPR125 antibody. The method isolating human spermatogonia are described in the materials and methods. We enriched GPR125 positive cells (GPR125^+^) from germ cells isolated from three OA patients and named them as SSC1, SSC2, and SSC3 for transcriptome sequencing. Furthermore, GPR125^+^ cells not only were able to form healthy colonies, but also were immunostained positive for spermatogonia markers, VASA, GPR125, CD90, UCHL1, GFRA1, and PLZF (Fig. 4a). The data indicated that the isolated cells by MACS using GPR125 antibody are of human spermatogonia characters. For SSCLCs, the SSCLCs were enriched by MACS using GPR125 antibody and confirmed the identity of spermatogonia by immunostaining for spermatogonia markers, VASA, GPR125, CD90, UCHL1, GFRA1, and PLZF. Figure 4b shows that enriched iPSC-SSCLCs at P1 were positive for VASA, GPR125, CD90, UCHL1, GFRA1, and PLZF. And the similar immunostaining results were got for the iPSC-SSCLCs at P7 and SHhES2-SSCLCs at P1 and P7 (data not shown).

Next, the transcript profiles (total 50,870 transcripts) of GPR125^+^ spermatogonia, hES2-SSCLCs, and hiPS-SSCLCs were compared by RNA sequencing. After principal component analysis (PCA) of the transcripts of hPSCs, SSCLCs, and GPR125^+^ spermatogonia (named SSCs), we found similar gene expression profiles of SSCLCs and SSCs. In contrast, the expression profile of SSCLCs was different from their parent hPSCs (Fig. S2a). Next, we selected significant genes downregulated or upregulated in SSCLCs compared to those in hPSCs (*P* < 0.001) and observed the expressions of 1042 genes among the groups of hES2, hiPS, hES2-SSCLCs, hiPS-SSCLCs, and SSCs (Table S2), and performed cluster and heatmap analysis. Figure 4c showed that the expression patterns of SSCLCs were similar to those of GPR125^+^ spermatogonia (Fig. 4c), but not to their parental hPSCs, especially for the pluripotency-related genes (Fig. S2b, Table S3). Moreover, the key genes included PLZF, MAGA4, PIWIL1, ETV5, DAZL, Boule, ID4, etc. (Fig. 4d, Table S3), which are important for SSC self-renewal and development, were significantly higher expressed in the groups of SSCLCs and GPR125^+^ cells compared to those in hPSCs (Fig. 4d). Additionally, the expression pattern of SSCLCs at P7 closely resembled those of GPR125^+^ spermatogonia (Fig. 4c, d), suggesting that SSCLCs maintained their SSC biological property even after passage. Next, genes of SSCLCs and in vivo GPR125^+^ spermatogonia that showed a more than 2-fold alteration were subjected to further Gene Ontology (GO) function enrichment analysis. We found that the expression levels of genes related to metabolic process and ribosome biogenesis were significantly downregulated compared to those of GPR125^+^ spermatogonia (Fig. 4e), suggesting that the basic metabolic features of SSCLCs were different from those of GPR125^+^ spermatogonia. Taken together, the above data indicated that SSCLCs display a similar expression pattern of spermatogonia markers and germ cell development-related genes to in vivo spermatogonia and that are different from their parent PSCs.

### SSCLCs were xeno-transplanted into the seminiferous tubules of mice

**Fig. 5:**
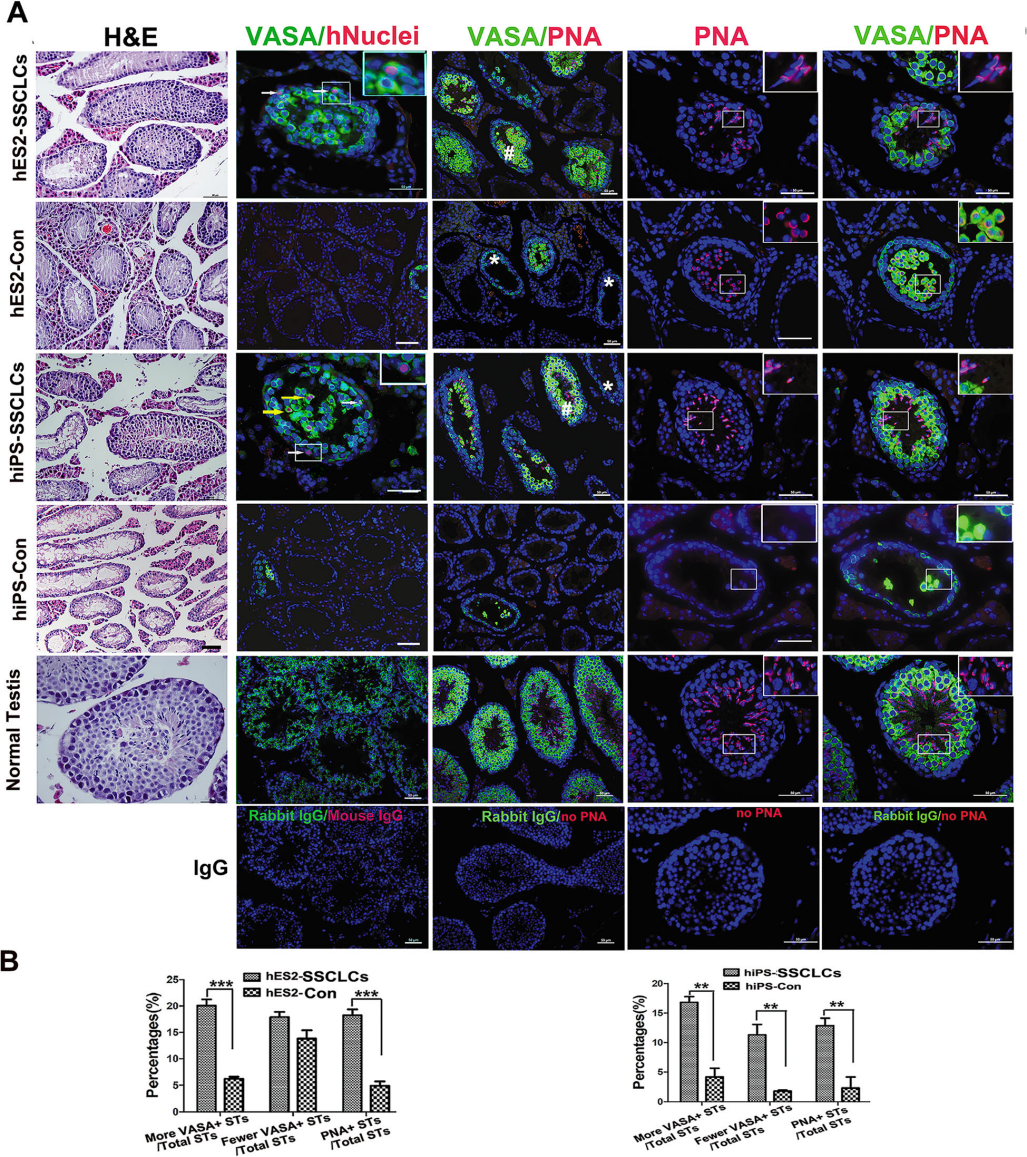
Xeno-transplantation of SSCLCs into the seminiferous tubules of recipient mice following busulfan treatment. **a** H&E staining or immunostaining of mouse testes at 5 weeks after SSCLCs transplantation. Immunostaining with VASA (green) and hNuclei (red) and PNA (red). Bright arrows indicate transplanted SSCLCs. The contralateral testes without grafts were used as internal control which was named as hES2-Con and hiPS-Con. Normal testis without busulfan treatment were used as positive control. Mouse IgG/corresponding Alexa Fluor 594, Rabbit IgG/corresponding Alexa Fluor 488, and no PNA staining were used as negative control. Pound keys (#) represent the seminiferous tubules with more VASA-positive cells, and asterisk (*) represents the seminiferous tubules with fewer VASA^+^ cells. Scale bars, 50 μm. **b** Quantification of seminiferous tubules with VASA^+^ cells or PNA+ cells in testes of different groups. STs represent seminiferous tubules. Five to six representative sections of testes were counted. At least three mice were used in every group. Data are represented as mean ± SEM

Germ cell transplantation is a good model for studying the biological function of male germ cells. Homologous transplantation of SSCs has been demonstrated to have the ability to generate spermatogenesis and restore fertile upon transplantation in many animal models including primates [36]. However, human germ cell transplantation is challenged by homologous transplantation into human testes to evaluate the spermatogenic potential [36]. By now, xeno-transplantation of hSSCs is considered to be the only reliable method to test for hSSC functionality. hSSCs were able to home on the base membrane of seminiferous tubules after xeno-transplanted into the testes of immunodeficient mice or adult azoospermia mouse testes [34, 37]. However, xeno-transplanted hSSCs cannot proceed spermatogenesis in mouse testes, and seldom divide and steadily decrease in number. To investigate the biological characteristics and function of SSCLCs in recipient testes, the SSCLCs at P3 enriched by MACS using GPR125 antibody were transplanted into the seminiferous tubules of unilateral testes of nude mice treated with busulfan, which is a toxin-destroying recipient SSCs and spermatogenesis [7, 38]. The contralateral testes without grafts were used as an internal control. Histological analysis of the testes at 5 weeks after cell transplantation were performed, the histological images show that busulfan significantly damaged spermatogenic cells and reduced the Johnsen’s score, which is a well-established method for evaluating spermatogenic function [39]. But the mean score of the transplantation groups were significantly higher than that in the control groups (Fig. 5a, Left panel, Fig. S3a and Table S4), suggesting that the SSCLCs could restore recipient spermatogenesis. Immunostaining images showed that many more VASA-positive germ cells appeared in the seminiferous tubules of the transplantation group compared to those of control groups (Fig. 5a), and the quantification was performed and the percentages of the seminiferous tubules with more VASA-positive cells over total seminiferous tubules were significantly higher in SSCLC transplanted groups than that of the control group (Fig. 5b). To evaluate the survival and position of transplanted SSCLCs, the seminiferous tubules were immunostained with the antibodies against human nuclei (hNuclei, a specific antibody for human cells) and VASA at week 2 and week 5 post-transplantation. As shown in Fig. 5a, the grafts could survive at least 5 weeks after transplantation, as demonstrated by hNuclei and VASA double-positive cells which appeared at the base membrane of seminiferous tubules and in the middle of seminiferous tubules 5 weeks post-transplantation (Fig. 5a). Moreover, more hNuclei-immunoreactive cells survived at 2 weeks post-grafting than that at 5 weeks post-grafting. However, hNuclei-immunoreactive cells only located in the middle of seminiferous tubules at 2 weeks post-grafting (Fig. 5a and Fig. S3b). These data revealed that SSCLCs could home at the base membrane of seminiferous tubules at least at 2 weeks after transplantation. Additionally, hNuclei-positive cells were negative for SCP3 or Acrosin (data not shown), indicating that SSCLCs did not further differentiate in recipient mice. Furthermore, no teratoma was formed after SSCLCs were transplanted, suggesting no pluripotent cells existed in the SSCLCs.

To further determine if SSCLCs could promote recipient mouse spermatogenesis, we analyzed recipient spermatogenesis by staining spermatozoa with lectin PNA conjugated with Alexa Fluor 594, which selectively binds to acrosome of sperms and can be used as a sperm or haploid spermatid marker [40]. Furthermore, the testicular sections were also immunostained with VASA. As shown in Fig. 5a and b, there was a higher percentage of seminiferous tubules with PNA-positive cells in the transplantation group than those of control. PNA-positive cells represent haploid spermatids including immature haploid cells and mature sperms as evidenced by VASA and PNA double-positive cells for haploid cells, and PNA-positive and VASA-negative with long cell nuclei for sperms (Fig. 5a, right rows). Interestingly, mature long sperms were observed in the seminiferous tubules of SSCLCs group at 5 weeks post-transplantation, but not in the internal control group (Fig. 5a, right rows), which indicated pro-spermatogenesis effects of SSCLCs on recipient mice.

On the other hand, the hiPS-SSCLCs at P7 which were enriched by MACS using GPR125 antibody were transplanted into unilateral testes of nude mice treated with busulfan. Five weeks later, the recipient mice were analyzed. VASA and hNuclei double-positive cells were detected at the basement of seminiferous tubules (Fig. S4a), and more, SSCLCs at P7 could promote recipient spermatogenesis (Fig. 4a, b). In contrast, fibroblast-like cells (FBs) derived from hiPS at P2 could not support recipient spermatogenesis (Fig. S4c, d). The above results implicated that SSCLCs could survive at least 5 weeks post-transplantation and home at the base membrane of seminiferous tubules and restore recipient spermatogenesis.

## Discussion

In the present study, we described a stepwise approach that efficiently generates grape-like SSCLCs derived from hPSCs after a 3-step induction (Fig. 1). The generated SSCLCs possess phenotypic, transcriptome, homing, and functional characteristics similar to their counterpart in vivo, i.e., enriched GPR125^+^ spermatogonia (Figs. 1, 2, 3, 4, and 5). More importantly, the SSCLCs can be propagated and sustained for approximately 4 months in vitro with a stable expression of SSC markers and homing property, displaying a profound expansion capability (Fig. 2). In the current study, we characterized the SSCLCs and hSSCs by immunoassays with not only a single marker GPR125 or CD90, but with a panel of other SSC markers, such as GFRA1, PLZF, UCHL1, and VASA (Fig. 2). In addition, we characterized the SSCLCs by the features of morphology, transcriptomics, and functional assays (Figs. 1, 2, 3, 4, and 5). Therefore, SSCLCs possess the characters of human spermatogonia and no other somatic cells.

Our stepwise protocol is composed of 3 steps: (1) induction of formation of PGCs, (2) generation of PGC clusters, and (3) differentiation into hSSC colonies. The first step is achieved by including insulin, transferrin, putrescine, bFGF, GDNF, and many kinds of fatty acids that facilitating the production of PGCs [12, 14, 41, 42]. In this way, many PGCLCs can be derived from hPSCs, which allows more efficient production of PGCs derived from hPSCs compared with previous approaches [12, 26, 27]. The second step is performed to generate PGC clusters by changing the medium containing RA and SCF, which enhances PGC proliferation [28, 29]. Because the formation of cell clusters is a common feature for multiple SSC culture system [34, 43, 44], which reflects the cystic nature for the spermatogonial growth in testes [45]. The cell clustering may facilitate the generation, survival, and proliferation of SSCs, which is similar to our previous study with cerebellar granule neuronal stem cells [46]. In the last step, specific growth factors and compounds including GDNF, bFGF, SCF, vitamin, RA, testosterone, and FSH are used to mimic the testicular niche for SSC production. Previously, Ishikura et al. employed a co-culture method by using embryonic testicular cells to provide the testicular growth factors and compounds to generate SSCLCs derived from mouse PSCs [47]. However, the co-culture strategy could result in inconsistent and uncontrolled differentiation efficiency and the production of heterogenous SSCLCs. Our study demonstrates that including specific growth factors and compounds at different steps is a better and repeatable approach to induce the production of SSCLCs from hPSCs.

In addition, in the study, only a small fraction haploid was produced by SSCLCs (2.54% ± 0.62% and 1.87 ± 0.35%) (Fig. 3c). There may be two reasons that account for this result. First, an efficient approach is required to be developed to differentiate SSCLCs into haploid cells. Sun et al. reported that only 6.1% of haploid cells were generated from hSSCs derived OA patients by two-dimensional-induced  (2D-I) differentiation system, but 17.9% of haploid cells were produced from hSSCs by three-dimensional-induced system (3D-I) [48]. In our study, we used 2D-I system to differentiate SSCLCs into haploid cells, thus, in the further research, 3D-I system is needed for the differentiation of SSCLCs into haploid cells. Second, some SSCLCs are not mature, evidenced by some SSCLCs that did not home at the bottom of seminiferous tubule. Therefore, how to differentiate hPSCs into more mature SSCs deserves more research in the future.

A mount of evidence demonstrates that stem cell niche has a major influence on the fate of the decision of stem cell self-renewal and differentiation [49–52]. A previous study reported that SSCs derived from older male mice with impaired spermatogenesis could regain the capability of spermatogenesis when transplanted into young testes, suggesting that self-renewal and differentiation potential of SSCs can be continually maintained in a young testicular niche [53]. Additionally, many studies showed that mesenchymal cells or Sertoli cells injected by inter-tubular route into infertile testis induced by busulfan could restore spermatogenesis [33, 54, 55], indicating mesenchymal cells or Sertoli cells can restore testicular function. Recently, researchers found that there exists a novel population of pluripotent stem cells termed very small embryonic-like stem cells (VSELs) in the testes which can survive chemotherapy. Mesenchymal cells or Sertoli cells transplanted into busulfan-treated testes could secret some paracrine factors to restore healthy stem niche and support surviving VSELs to undergo spermatogenesis [56, 57]. Surprisingly, our study showed that SSCLCs could restore the spermatogenesis of busulfan-treated recipient mice (Fig. 5, Figs. S3-S4 and Table S3), implicating that the grafted SSCLCs could secret some trophic factors contributing to the testicular stem cell niche, supporting the differentiation of surviving VSELs and restoring recipient spermatogenesis. Thus, further investigation is required to fully understand the molecular mechanism. Furthermore, SSCLCs could not continue to differentiate upon xeno-transplantation into busulfan-treated mouse testes, presumably because of interspecies difference. Consistent with our study, human SSCs could not differentiate upon xeno-transplantation into mouse testes [34, 58].

## Conclusion

In summary, this study demonstrates that expandable SSCLCs are efficiently generated from hPSCs by a 3-step approach. Moreover, the SSCLCs were similar to their counterpart human spermatogonia in view of their phenotype, protein and RNA expression, and functional properties, indicating that SSCLCs may be a suitable cell source of human SSCs for basic research and disease modeling.

## Supplementary information

**Additional file 1: Table S1.** Primers of real-time PCR for germ cell markers. **Tables S2.** List of 1042 transcripts and their normalized FPRM from RNA-seq in the group of hPSCs, SSCLCs and GPR125^+^ cells isolated from human testes. Related to Fig. 4. **Tables S3.** List of transcripts related to pluripotency, SSC markers, germ cells and their FPKM from RNA-seq in the group of hPSCs, SSCLCs and GPR125^+^ cells. Related to Fig. 4 and Figure S2. **Table S4.** SSCLCs restore recipient testicular spermatogenesis after transplantation at different time points by Johnsen’s Score. Related to Figure S3A.

**Additional file 2: Figure S1.** PGCs, spermatocytes and haploid cells were also generated from hES2 and hiPSC during hPSCs differentiation. **a** Quantitative real-time PCR of mRNA levels for germ cell marker genes expressed by hES2 and hiPS-differentiated cells at stage1–3. **b** Immunostaining images of hES2 and hiPS-differentiated cells after 1-step induction with PGC marker antibodies. Mouse and Rabbit IgG were used as negative control. Scale bars: 50 μm. **c** Immunostaining assay of hES2 with pluripotent markers , SSC markers and PGC markers. Scale bars: 50 μm. **d** Immunostaining of differentiated cells at stage 2 (S2) with OCT4 and SSEA1. Mouse and Rabbit IgG were used as negative control. Scale bars 50 μm. **e** Immunostaining of hES2- and hiPS-differentiated cells at S3 with Nanog. Scale bars: 50 μm.

**Additional file 3: Figure S2.** Transcriptome analyses of hPSCs, SSCLCs and human GPR125^+^cells isolated from human testes. **a** PCAon hPSCs, SSCLCs and human GPR125^+^cells **b** Heatmap on the transcript expression of pluripotency-related genes in the hES2, hiPS, SSCLCs and human GPR125^+^ cells. SLC represents SSCLCs. SSC1, SSC2 and SSC3 represent GPR125^+^ cells.

**Additional file 4: Figure S3.** SSCLCs promote recipient testicular spermatogenesis by H&E staining. **a** The section of mouse testes at 5 weeks after cell transplantation by H&E staining. Scale bars:100 μm. **b** Survival of SSCLCs grafts were detected by immunostaining with the antibodies against hNuclei and VASA. Scale bars:50 μm.

**Additional file 5: Figure S4.** SSCLCs but not hiPSC-derived fibroblasts promote recipient testicular spermatogenesis. H&E staining and immunostaining with VASA and hNuclei of mouse testes at 5 weeks after cells transplantation. **a** hiPSC-SSCLCs at P7 promoted recipient mouse testicular spermatogenesis. White arrow represents transplanted SSCLCs. Scale bars: 50 μm. **b** Quantification of the percentages of seminiferous tubules containing VASA^+^ cells over the total seminiferous tubules. STs represents seminiferous tubules. **c** hiPS-derived fibroblast (FBs) at P2 did not promote receipt mouse testicular spermatogenesis. post-transplantation White arrow represents transplanted FBs. Scale bars: 50 μm. **d** Quantification of the percentages of seminiferous tubules with VASA^+^ cells over total seminiferous tubules.

## Data Availability

All related data are available under request.

## Abbreviations

SSCs: Spermatogonial stem cells
hSSCs: Human spermatogonial stem cells
SSCLCs: Spermatogonial stem cells like cells
hPSCs: Human pluripotent stem cells
PGCs: Primary germ cells
RA: Retinoic acid
SCF: Stem cell factor
EGF: Epidermal factor
bFGF: Basic fibroblast growth factor
GDNF: Glial cell derived neurotrophic factor
LIF: Leukemia inhibitory factor
VC: Vitamin C
VA: Vitamin A
VE: Vitamin E
OA: Azoospermia
MACS: Magnetic-activated cell sorting
FISH: Fluorescence in situ hybridization
GO: Gene Ontology
