# Supplementary material for: Derivation and propagation of spermatogonial stem cells from human pluripotent cells
Source: Stem Cell Res Ther. 2020 Sep 23;11:408. doi: 10.1186/s13287-020-01896-0 (PMC7509941; doi:10.1186/s13287-020-01896-0)
Supplement: Supplementary file 1 — Additional file 1: Table S1. Primers of real-time PCR for germ cell markers. Tables S2. List of 1042 transcripts and their normalized FPRM from RNA-seq in the group of hPSCs, SSCLCs and GPR125+ cells isolated from human testes. Related to Fig. 4. Tables S3. List of transcripts related to pluripotency, SSC markers, germ cells and their FPKM from RNA-seq in the group of hPSCs, SSCLCs and GPR125+ cells. Related to Fig. 4 and Figure S2. Table S4. SSCLCs restore recipient testicular spermatogenesis after transplantation at different time points by Johnsen’s Score. Related to Figure S3A. [file 13287_2020_1896_MOESM1_ESM.zip › Table S4_ESM.docx]

| No | hES2-5W01 |  | hES2-5W02 | | hES2-5W03 | | hiPS-5W01 | | hiPS-5W02 | | hiPS-5W03 | |
| --- | --- | --- | --- | --- | --- | --- | --- | --- | --- | --- | --- | --- |
| Score | graft | con | graft | con | graft | con | graft | con | draft | con | draft | con |
| 1 | 2 | 20 | 0 | 0 | 14 | 30 | 0 | 24 | 11 | 30 | 10 | 41 |
| 2 | 95 | 124 | 79 | 108 | 60 | 58 | 74 | 68 | 90 | 55 | 58 | 60 |
| 3 | 23 | 23 | 15 | 16 | 20 | 15 | 16 | 1 | 12 | 3 | 15 | 2 |
| 4 | 7 | 5 | 14 | 11 | 4 | 5 | 11 | 0 | 3 | 0 | 0 | 0 |
| 5 | 10 | 2 | 32 | 7 | 0 | 0 | 6 | 0 | 15 | 4 | 23 | 8 |
| 6 | 5 | 3 | 0 | 4 | 7 | 2 | 0 | 0 | 3 | 0 | 0 | 0 |
| 7 | 18 | 5 | 8 | 2 | 0 | 3 | 0 | 0 | 0 | 0 | 0 | 0 |
| 8 | 0 | 1 | 3 | 3 | 5 | 4 | 2 | 0 | 3 | 1 | 5 | 5 |
| 9 | 2 | 0 | 14 | 2 | 10 | 0 | 9 | 0 | 10 | 0 | 15 | 2 |
| 10 | 6 | 0 | 0 | 0 | 0 | 0 | 0 | 0 | 0 | 0 | 0 | 0 |
| Mean | 3.4 | 2.3 | 3.8 | 2.8 | 3.2 | 2.5 | 3.1 | 1.8 | 4.2 | 1.9 | 3.7 | 2.2 |
| Score |  |  |  |  |  |  |  |  |  |  |  |  |
