# Supplementary material for: Derivation and propagation of spermatogonial stem cells from human pluripotent cells
Source: Stem Cell Res Ther. 2020 Sep 23;11:408. doi: 10.1186/s13287-020-01896-0 (PMC7509941; doi:10.1186/s13287-020-01896-0)
Supplement: Supplementary file 1 — Additional file 1: Table S1. Primers of real-time PCR for germ cell markers. Tables S2. List of 1042 transcripts and their normalized FPRM from RNA-seq in the group of hPSCs, SSCLCs and GPR125+ cells isolated from human testes. Related to Fig. 4. Tables S3. List of transcripts related to pluripotency, SSC markers, germ cells and their FPKM from RNA-seq in the group of hPSCs, SSCLCs and GPR125+ cells. Related to Fig. 4 and Figure S2. Table S4. SSCLCs restore recipient testicular spermatogenesis after transplantation at different time points by Johnsen’s Score. Related to Figure S3A. [file 13287_2020_1896_MOESM1_ESM.zip › Tables S2_ESM.pdf]

**gene_id**	gene name	hiPS_FPKM	hES2_FPKM	hiPS-SSCLCs-P1-1_FPKM	hiPS-SSCLCs-P1-2_FPKM	hiPS-SSCLCs-P7-1_FPKM	hiPS-SSCLCs-P7-2_FPKM	hES2-SSCLCs-P1-1_FPKM	hES2-SSC-P1-2_FPKM	SSC1_FPKM	SSC2_FPKM	SSC3_FPKM
ENSG00000088305	DNMT3B	622.774	648.715	0.499	0.297	0.946	0.110	0.559	0.135	0.297	1.273	1.233
ENSG00000240563	L1TD1	429.733	370.175	1.303	4.286	3.726	3.278	1.932	3.667	0.683	1.603	0.000
ENSG00000265992	ESRG	389.761	563.594	0.035	0.000	0.423	1.007	0.153	0.254	0.000	0.000	0.000
ENSG00000175130	MARCKSL1	345.051	289.960	30.922	40.309	21.728	27.704	28.591	21.295	36.501	22.281	12.403
ENSG00000131914	LIN28A	314.452	290.440	0.015	0.035	4.474	0.000	0.058	0.035	0.000	0.000	0.000
ENSG00000145423	SFRP2	312.107	394.938	0.000	0.000	0.788	0.000	0.236	0.000	0.000	0.000	0.119
ENSG00000169710	FASN	231.064	214.713	13.809	5.683	17.167	15.853	12.692	25.980	4.873	4.250	5.555
ENSG00000204531	POU5F1	229.914	197.223	10.740	6.944	14.278	10.306	12.073	6.594	0.000	0.000	0.000
ENSG00000155760	FZD7	220.814	215.598	0.339	0.097	0.321	0.096	0.373	0.302	2.101	2.334	2.071
ENSG00000130203	APOE	210.151	216.173	1.158	0.162	0.551	0.096	0.124	1.081	0.114	1.124	0.000
ENSG00000103257	SLC7A5	207.878	205.183	10.094	9.056	11.206	13.696	12.434	16.143	2.618	6.170	4.047
ENSG00000179899	PHC1P1	172.989	206.133	0.107	0.204	0.492	0.122	0.964	0.541	1.145	0.163	1.424
ENSG00000179051	RCC2	164.065	141.675	12.174	10.354	13.401	12.438	15.422	13.388	4.290	6.180	4.201
ENSG00000128567	PODXL	157.426	162.520	0.654	0.563	0.880	0.445	1.010	0.852	0.206	0.281	0.000
ENSG00000196924	FLNA	144.841	156.216	12.035	1.615	10.549	9.019	12.164	13.521	9.945	11.144	9.151
ENSG00000170027	YWHAG	139.777	135.207	11.801	8.198	10.175	10.443	19.814	26.409	26.062	16.231	16.793
ENSG00000165349	SLC7A3	133.300	97.426	0.000	0.000	1.991	0.000	0.000	0.000	0.000	0.000	0.000
ENSG00000272398	CD24	133.169	119.321	0.013	0.060	1.886	0.155	0.073	0.401	1.017	1.032	0.000
ENSG00000188486	H2AFX	125.074	102.412	8.758	1.841	10.907	4.609	13.483	8.561	3.448	12.841	3.243
ENSG00000137267	TUBB2A	124.072	132.002	8.469	7.781	15.809	6.824	11.622	9.086	16.499	18.189	6.312
ENSG00000088325	TPX2	111.315	133.418	9.101	12.133	26.374	6.502	29.664	33.119	22.309	24.471	16.394
ENSG00000101057	MYBL2	109.564	89.729	10.163	16.141	12.968	10.348	9.575	5.422	4.343	2.361	6.330
ENSG00000143799	PARP1	105.100	118.323	20.148	12.224	29.158	12.030	25.088	12.787	7.715	5.648	4.326
ENSG00000107581	EIF3A	102.918	101.238	10.463	7.854	9.371	8.127	12.772	15.906	10.712	10.981	9.461
ENSG00000116560	SFPQ	101.803	95.105	6.429	6.421	5.414	5.643	9.029	9.355	9.768	12.094	16.273
ENSG00000140988	RPS2	100.830	80.448	13.853	15.668	9.365	16.679	4.108	5.697	10.392	5.742	11.381
ENSG00000153187	HNRNPU	99.550	83.353	10.039	7.228	6.753	7.478	13.154	14.571	13.608	12.605	14.458
ENSG00000158246	FAM46B	93.833	81.509	3.496	5.555	4.000	5.045	3.738	6.245	1.296	1.268	2.205
ENSG00000070756	PABPC1	91.945	88.505	8.272	10.013	16.676	8.925	31.263	52.979	24.998	20.609	21.400
ENSG00000008083	JARID2	91.848	67.398	2.767	2.012	1.798	2.131	2.974	7.883	5.426	5.845	11.279
ENSG00000175928	LRRN1	88.171	70.619	0.066	0.000	0.479	0.168	0.000	0.615	0.000	0.201	0.000
ENSG00000206557	TRIM71	84.278	83.423	0.000	0.000	0.275	0.032	0.000	0.056	0.404	2.266	0.809
ENSG00000206557	TRIM71	84.278	83.423	0.000	0.000	0.275	0.032	0.000	0.056	0.404	2.266	0.809

ENSG00000119888	EPCAM	83.883	86.190	0.229	0.642	2.529	0.695	0.945	0.959	5.476	5.111	5.374
ENSG00000166831	RBPMS2	83.308	71.072	0.442	0.117	1.160	0.000	0.407	0.727	0.821	0.465	0.000
ENSG00000108424	KPNB1	80.574	70.957	14.136	13.058	13.419	10.567	27.830	27.259	14.129	9.096	10.270
ENSG00000133980	VRTN	79.653	55.251	0.000	0.000	0.133	0.000	0.034	0.000	0.000	0.000	0.000
ENSG00000008311	AASS	79.350	66.657	0.168	0.289	0.728	0.658	2.143	4.177	1.042	1.715	5.354
ENSG00000086475	SEPHS1	76.728	68.642	6.027	7.033	5.393	3.940	4.456	7.134	7.838	5.171	6.150
ENSG00000134323	MYCN	75.580	61.690	0.000	0.000	0.204	0.000	0.000	0.000	0.000	0.256	0.000
ENSG00000176619	LMNB2	74.988	59.524	11.391	4.509	4.470	6.219	3.339	9.885	3.074	2.196	3.312
ENSG00000046604	DSG2	73.794	76.347	0.000	0.019	1.225	0.043	0.043	0.562	2.279	1.961	0.565
ENSG00000183684	ALYREF	71.994	60.326	6.522	7.161	3.277	3.208	2.853	3.265	5.556	7.019	6.387
ENSG00000102096	PIM2	71.939	104.162	10.194	5.946	6.536	14.037	4.231	7.806	5.251	4.176	3.371
ENSG00000004478	FKBP4	71.516	74.787	19.504	16.966	6.687	10.286	5.239	9.518	4.289	6.372	2.032
ENSG00000128245	YWHAH	71.025	54.633	3.117	8.071	5.106	3.370	4.971	5.276	16.426	16.493	11.891
ENSG00000173473	SMARCC1	70.586	61.740	9.201	6.455	6.109	5.420	4.993	13.620	5.516	6.858	5.256
ENSG00000130402	ACTN4	69.719	69.479	5.004	4.845	9.500	9.928	10.238	5.959	5.803	7.473	3.947
ENSG00000104332	SFRP1	69.179	18.948	0.105	0.151	0.203	0.146	0.166	0.081	0.577	0.563	0.380
ENSG00000147601	TERF1	68.043	58.617	1.410	1.398	1.012	0.644	2.806	1.546	2.030	3.108	3.316
ENSG00000112972	HMGCS1	67.829	59.534	6.722	4.541	8.791	8.618	21.344	21.331	8.311	8.304	13.534
ENSG00000095002	MSH2	67.600	54.358	1.027	1.229	4.514	1.469	13.965	1.485	5.648	6.632	5.061
ENSG00000133026	MYH10	66.969	56.254	3.401	2.050	4.208	2.615	2.557	2.021	0.369	2.245	1.240
ENSG00000063244	U2AF2	65.903	47.442	2.241	1.997	2.015	6.759	5.652	8.215	0.975	2.143	2.714
ENSG00000125144	MT1G	65.295	66.783	4.122	1.864	0.189	0.529	0.439	0.000	3.268	4.423	8.601
ENSG00000197451	HNRNPAB	62.988	52.306	9.815	9.900	5.143	7.271	5.200	4.939	8.622	8.543	5.788
ENSG00000163931	TKT	61.812	73.284	10.196	12.086	8.786	16.627	10.381	16.230	3.500	12.492	2.190
ENSG00000177733	HNRNPA0	61.083	56.290	5.496	1.876	5.113	3.358	2.080	5.126	4.244	2.695	8.799
ENSG00000035403	VCL	60.191	30.316	0.531	0.641	1.954	1.002	7.964	9.937	7.113	5.749	9.278
ENSG00000197102	DYNC1H1	59.522	62.001	5.319	5.386	4.306	5.100	10.867	12.186	7.137	7.839	10.702
ENSG00000205339	IPO7	59.327	66.529	3.414	3.756	3.326	2.914	12.816	11.419	6.611	10.390	9.295
ENSG00000108468	CBX1	58.667	59.354	4.912	2.548	6.127	2.845	11.484	8.751	6.290	10.601	8.251
ENSG00000165821	SALL2	56.216	43.011	0.501	0.232	0.599	0.487	0.531	1.013	0.283	0.457	0.000
ENSG00000152284	TCF7L1	55.941	34.695	0.209	0.000	0.245	0.119	0.132	0.222	1.727	1.172	0.722
ENSG00000164985	PSIP1	55.103	64.550	2.112	1.975	1.977	1.404	4.498	3.827	6.257	4.740	6.545
ENSG00000174231	PRPF8	54.621	63.241	6.491	2.895	6.766	5.622	14.685	13.916	6.484	7.317	13.418
ENSG00000111642	CHD4	54.395	53.357	13.741	9.095	5.600	6.381	12.135	7.152	6.882	9.391	6.206
ENSG00000107438	PDLIM1	53.068	57.453	1.220	1.788	9.304	0.810	3.200	0.324	10.766	11.874	11.448

ENSG00000089280	FUS	52.852	37.043	26.802	15.867	23.844	18.990	33.180	21.879	2.584	3.800	2.874
ENSG00000112759	SLC29A1	52.095	59.589	5.045	15.622	4.612	10.980	3.475	12.782	2.343	2.660	0.637
ENSG00000114767	RRP9	51.682	37.662	66.810	67.421	55.008	150.436	112.914	70.181	19.026	26.631	26.447
ENSG00000007520	TSR3	50.069	47.793	9.896	12.900	2.445	7.853	2.797	10.080	14.263	19.406	14.433
ENSG00000065150	IPO5	49.780	49.482	2.910	3.382	3.427	2.472	4.034	9.420	11.327	12.323	8.390
ENSG00000004487	KDM1A	49.421	56.736	4.298	4.101	2.784	2.995	6.001	5.394	10.391	10.425	10.125
ENSG00000078369	GNB1	47.710	40.139	4.794	8.335	4.660	5.058	9.312	10.852	10.554	12.854	11.665
ENSG00000110917	MLEC	46.148	62.363	3.453	3.901	0.144	0.899	3.852	5.807	8.806	8.469	8.943
ENSG00000143384	MCL1	45.385	62.252	2.061	2.192	2.799	2.160	6.062	6.732	6.559	8.689	9.587
ENSG00000168286	THAP11	44.490	43.549	9.848	8.867	5.528	6.072	8.406	8.296	6.220	6.705	3.781
ENSG00000121774	KHDRBS1	44.339	44.205	2.294	6.517	3.384	3.469	2.187	2.600	2.008	3.019	2.774
ENSG00000179059	ZFP42	44.082	32.323	0.000	0.000	0.750	0.000	0.028	0.000	0.210	0.406	0.621
ENSG00000064666	CNN2	44.056	35.774	3.274	7.698	7.304	3.537	4.383	3.715	8.165	4.903	7.507
ENSG00000111752	PHC1	43.807	47.342	0.066	0.124	0.262	0.092	0.224	0.196	2.032	1.699	0.921
ENSG00000136110	LECT1	43.466	71.374	0.177	0.712	0.288	2.005	0.251	1.421	0.000	2.035	0.000
ENSG00000113758	DBN1	43.228	33.425	8.196	1.842	9.717	7.403	9.410	13.351	1.375	3.422	3.016
ENSG00000147596	PRDM14	42.921	38.778	0.000	0.000	0.000	0.000	0.000	0.000	0.000	0.000	0.000
ENSG00000174721	FGFBP3	42.259	42.052	0.814	0.540	0.139	0.073	0.162	0.289	0.000	0.262	0.000
ENSG00000188322	SBK1	42.215	35.980	0.742	0.050	0.301	0.149	0.316	0.049	0.263	0.301	0.000
ENSG00000111371	SLC38A1	42.059	39.815	5.222	4.800	6.996	5.436	4.174	5.864	2.950	3.444	1.384
ENSG00000184575	XPOT	41.583	49.131	8.251	7.097	5.412	8.318	3.593	9.201	12.008	20.253	16.115
ENSG00000139514	SLC7A1	41.299	38.056	3.177	1.912	4.404	3.462	7.727	7.829	3.563	8.376	4.493
ENSG00000184697	CLDN6	41.129	34.397	0.092	0.455	1.538	0.652	0.211	0.052	0.000	11.307	3.597
ENSG00000117724	CENPF	41.002	45.323	3.094	2.428	2.325	1.434	6.485	4.210	4.774	2.006	2.171
ENSG00000126787	DLGAP5	40.710	49.508	1.045	1.634	5.863	0.709	6.835	5.003	3.246	5.027	4.063
ENSG00000130294	KIF1A	39.885	32.945	0.356	0.096	0.431	0.006	0.305	0.170	0.122	0.301	0.000
ENSG00000160710	ADAR	39.538	39.282	5.620	2.311	3.479	3.470	8.617	8.221	5.065	6.885	7.932
ENSG00000050344	NFE2L3	39.095	39.071	0.105	0.100	0.255	0.388	0.748	2.064	1.967	2.834	0.039
ENSG00000178035	IMPDH2	38.095	42.972	42.794	60.353	86.420	60.046	89.237	63.373	14.872	16.047	10.136
ENSG00000124541	RRP36	37.943	23.827	5.335	5.535	1.341	4.192	2.932	4.026	7.793	7.904	6.399
ENSG00000066135	KDM4A	37.868	39.538	3.985	2.021	4.731	3.908	10.239	13.333	0.308	0.147	0.085
ENSG00000177383	MAGEF1	37.539	43.791	4.930	8.097	5.195	2.615	8.513	6.335	2.813	3.965	0.444
ENSG00000241697	TMEFF1	37.389	41.106	0.125	0.479	0.509	0.000	0.788	0.000	3.273	0.894	1.392
ENSG00000138160	KIF11	37.090	35.569	1.346	1.234	3.352	1.129	4.340	2.902	6.808	5.765	10.167
ENSG00000187678	SPRY4	36.975	44.150	1.964	0.737	1.113	0.973	2.404	3.785	3.872	4.785	4.055

ENSG00000160973	FOXH1	36.589	30.705	0.109	0.000	0.296	0.000	0.000	0.409	0.000	0.000	0.000
ENSG00000064419	TNPO3	36.203	40.231	8.897	2.014	10.159	6.668	7.572	13.797	3.597	7.886	5.216
ENSG00000138668	HNRNPD	36.185	33.552	5.163	5.250	3.917	3.543	3.305	2.522	6.151	9.247	8.760
ENSG00000106278	PTPRZ1	36.021	39.612	0.000	0.009	0.000	0.000	0.069	0.607	0.000	0.000	0.556
ENSG00000141101	NOB1	35.860	35.752	19.408	31.014	39.395	29.852	54.098	34.591	6.685	12.523	13.908
ENSG00000177700	POLR2L	35.424	24.574	597.598	296.819	198.813	243.037	85.611	74.524	426.102	249.859	242.863
ENSG00000101193	GID8	35.308	42.475	3.969	5.292	5.230	3.269	4.658	9.179	10.764	9.639	8.359
ENSG00000136813	KIAA0368	35.195	35.743	2.008	1.888	2.218	1.619	2.495	3.222	3.774	4.792	4.149
ENSG00000143933	CALM2	35.132	30.704	61.772	62.881	245.267	160.199	101.075	149.702	226.517	217.669	263.975
ENSG00000188986	NELFB	35.123	37.486	4.523	8.787	6.600	10.390	5.352	6.394	5.349	6.239	3.856
ENSG00000204469	PRRC2A	34.568	35.876	3.149	1.366	4.699	4.594	5.238	4.198	4.432	3.964	3.695
ENSG00000086758	HUWE1	34.224	36.298	3.986	1.504	2.705	2.968	5.517	5.446	4.048	5.686	4.598
ENSG00000104635	SLC39A14	34.123	28.977	0.941	1.078	3.584	1.387	3.843	11.637	9.300	6.502	5.396
ENSG00000182871	COL18A1	33.973	36.159	0.191	0.164	0.194	0.122	0.475	0.483	1.958	0.497	1.103
ENSG00000012660	ELOVL5	33.665	36.481	1.767	2.533	4.803	2.523	8.019	5.077	5.808	8.948	7.234
ENSG00000140545	MFGE8	33.122	23.091	4.542	2.494	3.630	2.530	2.225	4.350	3.536	2.760	2.921
ENSG00000124486	USP9X	32.741	62.699	3.413	4.256	4.027	5.096	6.196	8.254	10.031	8.471	8.158
ENSG00000128602	SMO	32.542	26.859	0.385	0.104	0.405	0.387	0.057	0.153	0.000	0.000	0.000
ENSG00000157227	MMP14	32.261	22.483	1.412	0.125	2.529	1.313	3.054	1.701	2.533	2.909	9.294
ENSG00000141985	SH3GL1	32.209	30.335	6.551	5.179	8.103	6.474	7.321	8.965	6.221	7.401	4.861
ENSG00000241186	TDGF1	32.159	28.054	0.000	0.000	0.036	0.000	0.047	0.000	0.357	0.000	0.000
ENSG00000147676	MAL2	32.010	42.734	0.000	0.000	0.297	0.072	0.265	0.000	1.751	6.877	5.930
ENSG00000153201	RANBP2	31.838	33.511	1.950	1.893	1.051	1.133	6.346	9.907	10.544	11.350	9.404
ENSG00000178921	PFAS	31.661	27.186	4.866	4.359	5.302	3.954	4.063	2.931	1.060	2.915	3.631
ENSG00000117523	PRRC2C	31.639	33.682	5.620	6.153	3.071	4.877	6.795	6.171	6.851	3.939	4.477
ENSG00000125107	CNOT1	31.463	37.346	1.959	2.641	2.472	1.647	3.208	2.791	4.431	5.612	5.079
ENSG00000009954	BAZ1B	31.448	28.664	4.388	2.690	2.601	2.609	4.911	3.455	4.160	4.416	6.325
ENSG00000173848	NET1	31.089	31.324	2.130	1.770	4.785	2.245	4.737	8.830	6.381	8.182	10.917
ENSG00000146904	EPHA1	30.927	31.011	0.063	0.145	0.502	0.352	0.060	0.250	0.255	0.097	0.528
ENSG00000130764	LRRC47	30.270	38.192	4.640	6.891	3.858	4.394	5.772	6.166	6.107	6.322	2.062
ENSG00000175216	CKAP5	30.205	41.225	1.686	1.795	2.865	1.423	8.743	8.493	4.129	5.975	8.330
ENSG00000085449	WDFY1	30.080	28.122	1.110	0.318	2.054	0.849	10.291	11.585	3.280	9.736	5.149
ENSG00000105323	HNRNPUL1	29.616	26.163	2.770	1.911	6.049	4.636	4.013	4.582	3.906	4.287	4.566
ENSG00000143321	HDGF	29.212	20.014	7.242	6.538	5.701	5.689	7.023	7.428	5.105	5.702	4.703
ENSG00000167635	ZNF146	29.187	32.075	5.756	3.957	4.030	3.999	3.681	5.557	6.170	7.306	6.650

ENSG00000101115	SALL4	29.161	25.163	0.982	0.439	1.401	1.053	2.714	1.440	0.000	0.898	0.000
ENSG00000141736	ERBB2	29.077	18.163	0.540	0.427	0.469	0.297	0.680	0.845	0.262	0.285	0.028
ENSG00000119139	TJP2	28.913	26.174	1.556	1.594	0.869	0.461	1.743	0.428	1.862	1.148	0.981
ENSG00000221823	PPP3R1	28.807	24.148	1.200	1.066	0.780	1.254	1.982	2.244	3.859	1.285	6.932
ENSG00000114631	PODXL2	28.493	29.654	3.662	5.260	7.249	8.808	8.506	7.097	2.401	2.441	3.148
ENSG00000102241	HTATSF1	28.208	36.936	2.823	3.436	4.679	1.996	5.406	4.811	5.791	6.571	4.683
ENSG00000174695	TMEM167A	28.173	26.103	1.794	3.689	4.247	2.894	10.818	8.941	8.261	7.201	8.140
ENSG00000065911	MTHFD2	28.065	38.440	2.984	6.817	6.616	5.633	8.662	9.773	5.792	9.887	8.034
ENSG00000198728	LDB1	27.913	27.006	4.019	2.230	3.264	6.970	3.900	4.143	1.720	2.777	4.206
ENSG00000104112	SCG3	27.563	22.993	0.000	0.000	0.554	0.000	0.237	0.013	0.946	1.836	0.628
ENSG00000060982	BCAT1	27.218	19.283	1.866	2.115	1.606	1.773	3.736	3.000	9.181	9.841	3.782
ENSG00000115306	SPTBN1	27.177	24.421	1.871	1.315	2.127	1.597	6.773	6.318	7.144	6.271	7.134
ENSG00000158710	TAGLN2	27.047	24.015	222.611	92.880	278.153	286.733	131.649	200.543	131.325	147.021	120.555
ENSG00000066735	KIF26A	26.970	29.776	0.152	0.052	0.074	0.000	0.105	0.026	0.397	0.000	0.236
ENSG00000130227	XPO7	26.501	29.165	3.295	3.561	4.743	4.745	4.782	5.242	4.186	2.474	4.369
ENSG00000152102	FAM168B	26.468	24.249	3.550	8.765	7.170	4.322	18.123	16.711	6.982	14.278	9.916
ENSG00000187109	NAP1L1	25.934	26.635	2.207	3.285	3.352	1.999	7.646	6.205	6.765	8.722	10.224
ENSG00000162551	ALPL	25.334	45.430	0.052	0.059	1.438	0.029	0.411	0.251	0.035	0.289	1.222
ENSG00000159399	HK2	25.245	20.997	2.890	2.130	5.156	4.918	2.924	6.156	7.298	3.527	4.587
ENSG00000135052	GOLM1	25.081	22.609	0.659	3.708	4.004	3.725	6.009	7.464	8.334	6.342	6.036
ENSG00000255508	RP11-864I4.1	24.785	87.172	3.781	2.390	1.933	1.747	2.369	6.935	6.453	4.911	0.041
ENSG00000131899	LLGL1	24.774	24.949	0.753	0.059	0.176	0.272	0.112	0.115	0.051	0.137	0.028
ENSG00000141543	EIF4A3	24.644	20.018	32.405	88.051	147.202	87.570	109.942	85.181	8.750	29.830	11.539
ENSG00000130768	SMPDL3B	24.617	13.562	0.125	0.029	0.732	0.299	0.158	0.141	0.000	0.575	0.000
ENSG00000169764	UGP2	24.108	23.250	0.452	0.782	3.091	1.318	5.583	8.105	9.601	7.460	8.133
ENSG00000043355	ZIC2	24.087	9.256	0.040	0.092	0.016	0.068	0.013	0.000	0.483	1.225	2.001
ENSG00000091409	ITGA6	23.953	20.333	2.121	2.160	2.561	3.367	2.140	5.571	9.132	3.715	4.809
ENSG00000082898	XPO1	23.200	22.945	1.628	2.332	3.267	1.169	6.350	4.062	7.199	5.789	8.532
ENSG00000107164	FUBP3	23.124	22.132	1.327	1.408	2.022	1.412	8.557	8.597	7.639	8.770	8.662
ENSG00000179115	FARSA	22.987	18.493	228.982	199.178	212.683	249.807	98.318	73.855	7.565	6.989	5.159
ENSG00000198087	CD2AP	22.808	21.939	0.534	0.563	0.997	0.801	3.417	7.895	7.662	7.002	5.212
ENSG00000163251	FZD5	22.712	16.642	100.904	11.197	18.872	14.824	82.182	23.091	0.000	0.696	0.217
ENSG00000101447	FAM83D	22.403	28.214	2.426	1.335	5.497	1.470	3.774	6.305	2.975	3.801	5.930
ENSG00000105447	GRWD1	22.257	18.477	58.044	36.927	90.765	66.945	68.783	43.802	2.526	6.584	5.087
ENSG00000186193	SAPCD2	22.197	28.500	6.090	3.670	8.282	5.197	6.597	5.291	0.114	0.000	0.126
ENSG00000169679	BUB1	22.161	29.249	0.739	0.917	3.238	0.610	3.581	5.475	5.092	6.211	3.474

ENSG00000128045	RASL11B	22.101	19.840	0.000	0.029	0.349	0.065	0.143	0.171	0.915	4.641	2.640
ENSG00000170522	ELOVL6	21.914	14.949	2.022	2.591	4.275	2.436	2.579	2.670	2.132	2.002	4.141
ENSG00000243709	LEFTY1	21.811	29.336	0.051	0.000	0.041	0.044	0.064	0.029	0.336	0.308	0.000
ENSG00000137693	YAP1	21.748	22.017	0.270	0.596	0.177	0.541	0.308	1.374	3.604	2.560	4.227
ENSG00000127481	UBR4	21.626	22.979	4.950	2.668	1.316	1.872	2.523	2.978	2.669	3.268	3.343
ENSG00000074842	MYDGF	21.537	18.555	293.986	297.761	181.029	347.256	90.939	108.043	329.892	278.460	378.813
ENSG00000155827	RNF20	21.310	18.051	1.506	1.705	2.682	1.582	3.249	3.277	5.279	2.629	2.017
ENSG00000160963	COL26A1	21.302	20.224	0.000	0.000	0.160	0.000	0.000	0.000	0.000	0.515	0.000
ENSG00000062038	CDH3	21.248	22.190	0.012	0.040	0.588	0.030	0.029	0.945	0.559	0.319	0.000
ENSG00000092421	SEMA6A	21.117	25.725	0.057	0.013	0.579	0.058	0.132	0.131	0.444	0.095	0.000
ENSG00000168411	RFWD3	21.112	14.777	0.632	0.552	1.996	0.788	7.658	4.740	1.492	6.138	4.859
ENSG00000180340	FZD2	21.096	15.670	0.052	0.059	0.419	0.132	0.000	0.174	1.735	0.869	0.670
ENSG00000186298	PPP1CC	21.012	29.219	2.838	3.064	1.986	1.804	6.201	2.766	3.635	5.612	4.347
ENSG00000137815	RTF1	20.952	23.576	6.038	4.566	2.094	2.368	5.650	5.793	5.512	8.065	3.880
ENSG00000114554	PLXNA1	20.938	19.349	3.236	3.103	2.593	5.764	3.144	5.327	3.875	2.154	2.644
ENSG00000105426	PTPRS	20.826	23.045	5.678	1.948	2.426	2.329	4.331	7.372	5.948	5.198	6.871
ENSG00000140575	IQGAP1	20.825	19.867	0.604	0.399	0.751	0.715	2.746	3.860	3.744	3.574	3.254
ENSG00000143375	CGN	20.680	15.484	0.000	0.093	0.999	0.035	0.655	0.160	0.000	0.620	0.000
ENSG00000197694	SPTAN1	20.661	19.909	2.709	1.980	2.012	1.516	3.705	5.152	5.465	6.107	3.201
ENSG00000130182	ZSCAN10	20.626	32.482	0.000	0.000	0.061	0.000	0.027	0.018	0.000	0.000	0.000
ENSG00000130182	ZSCAN10	20.626	32.482	0.000	0.000	0.061	0.000	0.027	0.000	0.000	0.000	0.000
ENSG00000100983	GSS	20.504	21.413	117.566	108.884	101.105	108.674	121.793	97.719	20.856	32.969	12.138
ENSG00000104413	ESRP1	20.350	21.073	0.000	0.000	0.457	0.009	0.045	0.046	0.000	0.078	0.000
ENSG00000164024	METAP1	20.303	20.435	2.093	2.636	6.195	2.639	7.497	6.640	8.257	8.480	5.144
ENSG00000108091	CCDC6	20.228	17.491	2.515	1.987	1.525	1.520	8.227	4.963	4.331	4.898	7.060
ENSG00000105968	H2AFV	20.196	19.664	3.567	3.092	2.717	1.602	7.241	5.483	5.456	7.599	8.041
ENSG00000215301	DDX3X	20.189	44.756	1.665	2.317	1.973	2.039	2.753	2.913	9.303	14.129	4.022
ENSG00000132382	MYBBP1A	20.178	16.102	5.906	2.257	12.925	5.753	18.086	24.265	5.967	6.666	2.189
ENSG00000189306	RRP7A	20.158	13.971	130.973	106.989	59.851	60.490	45.946	35.201	5.988	13.707	7.205
ENSG00000106211	HSPB1	20.143	18.923	641.843	699.397	168.291	385.138	94.307	125.251	299.469	213.573	392.731
ENSG00000116679	IVNS1ABP	20.048	19.481	2.184	3.151	6.311	2.781	3.555	20.106	2.376	3.383	5.292
ENSG00000022556	NLRP2	20.036	9.770	0.000	0.000	1.064	0.009	0.025	0.000	1.054	0.339	0.000
ENSG00000128829	EIF2AK4	19.946	24.676	4.926	2.266	1.036	2.060	2.992	2.152	5.581	4.144	6.018
ENSG00000069248	NUP133	19.917	24.091	1.094	1.165	2.425	1.427	2.179	3.128	2.985	6.767	5.522
ENSG00000156925	ZIC3	19.794	43.414	0.000	0.000	0.016	0.000	0.000	0.000	1.282	0.059	1.460

ENSG00000173915	USMG5	19.411	18.843	271.376	366.549	129.252	187.194	67.758	63.488	130.557	89.325	78.233
ENSG00000123200	ZC3H13	19.405	20.537	5.110	3.331	2.905	2.608	10.739	8.912	4.661	5.776	4.142
ENSG00000039068	CDH1	19.281	18.398	0.015	0.009	0.649	0.006	0.014	0.008	0.000	0.023	0.084
ENSG00000086232	EIF2AK1	19.280	16.111	3.195	2.600	2.344	2.661	5.661	5.001	6.165	5.519	5.039
ENSG00000100422	CERK	19.222	19.651	1.890	3.799	3.150	3.260	4.661	7.477	5.015	6.947	5.114
ENSG00000001497	LAS1L	19.158	17.456	5.254	9.140	6.237	6.733	3.300	5.507	3.872	2.803	1.643
ENSG00000197785	ATAD3A	19.132	19.056	6.774	7.789	7.008	6.814	4.857	5.042	1.773	2.334	1.748
ENSG00000128849	CGNL1	19.080	16.451	0.000	0.000	0.213	0.000	0.039	0.000	0.000	0.057	0.000
ENSG00000112685	EXOC2	19.031	21.297	3.115	2.882	4.398	2.567	3.938	3.212	8.164	5.092	2.912
ENSG00000180182	MED14	18.824	26.512	0.385	0.486	0.258	0.543	1.935	3.187	1.317	2.536	2.554
ENSG00000138336	TET1	18.764	17.750	0.760	0.431	0.381	0.381	1.009	0.693	0.731	0.269	0.052
ENSG00000176105	YES1	18.660	25.508	1.795	2.278	2.644	1.335	5.016	11.820	7.938	10.120	4.501
ENSG00000112742	TTK	18.660	15.934	0.740	0.675	2.679	0.675	2.142	6.958	1.285	5.741	3.851
ENSG00000101544	ADNP2	18.518	19.819	1.895	2.156	1.457	0.921	0.459	3.543	5.364	1.271	7.905
ENSG00000134590	FAM127A	18.102	10.282	148.477	130.588	135.207	223.275	144.296	137.291	113.737	124.172	138.594
ENSG00000100836	PABPN1	17.888	15.762	8.486	8.473	2.605	6.191	4.081	6.868	5.606	2.319	1.384
ENSG00000133818	RRAS2	17.819	18.261	0.135	0.463	0.298	0.157	0.370	0.557	2.730	2.489	3.941
ENSG00000091009	RBM27	17.773	20.135	3.189	1.647	2.603	2.264	6.698	5.578	3.650	6.618	5.227
ENSG00000100811	YY1	17.757	18.197	2.442	2.136	0.198	1.027	3.075	2.504	5.019	6.075	2.915
ENSG00000114353	GNAI2	17.547	21.066	7.116	2.429	1.559	3.059	1.329	2.980	4.664	4.956	4.111
ENSG00000197417	SHPK	17.451	11.614	1.874	1.726	8.627	3.127	13.640	12.287	0.000	0.000	0.000
ENSG00000126464	PRR12	17.416	14.944	0.973	0.199	0.320	0.514	0.262	0.508	0.210	0.514	0.720
ENSG00000042753	AP2S1	17.398	19.956	253.257	174.083	85.627	113.076	48.892	40.190	179.183	119.313	138.899
ENSG00000100412	ACO2	17.281	19.126	19.538	28.578	37.502	35.300	63.442	51.436	7.693	9.550	13.943
ENSG00000144730	IL17RD	17.180	16.427	0.008	0.036	0.071	0.027	0.060	0.085	0.956	1.334	0.616
ENSG00000132297	HHLA1	17.099	34.336	0.000	0.000	0.000	0.000	0.000	0.000	0.000	0.000	0.000
ENSG00000075340	ADD2	16.928	15.529	0.117	0.187	0.199	0.095	0.286	1.023	0.023	0.357	0.000
ENSG00000248333	CDK11B	16.927	14.586	8.123	5.443	2.659	3.592	5.361	3.905	3.959	5.649	1.696
ENSG00000248333	CDK11B	16.927	14.586	8.123	5.443	2.659	3.592	5.361	3.905	3.959	5.649	1.696
ENSG00000171794	UTF1	16.855	17.914	0.000	0.000	0.000	0.161	0.000	0.000	1.136	1.306	1.256
ENSG00000068796	KIF2A	16.851	14.624	2.203	1.067	2.028	1.588	7.818	6.079	5.775	8.390	4.316
ENSG00000004864	SLC25A13	16.788	16.853	1.684	2.104	5.101	2.328	3.433	3.341	5.586	5.152	1.085
ENSG00000100726	TELO2	16.641	14.854	2.760	1.869	1.776	3.330	1.316	2.345	1.034	1.365	1.153
ENSG00000167680	SEMA6B	16.515	16.024	0.201	0.051	0.072	0.000	0.000	0.000	0.359	0.000	0.000
ENSG00000169018	FEM1B	16.509	21.288	1.084	0.860	1.267	1.037	2.475	1.165	2.214	3.043	1.499

ENSG00000081913	PHLPP1	16.451	14.846	0.198	0.152	0.215	0.235	0.562	0.285	2.039	0.961	7.276
ENSG00000187140	FOXD3	16.124	11.334	0.000	0.000	0.000	0.000	0.000	0.000	0.000	0.000	0.348
ENSG00000136193	SCRN1	16.122	23.690	1.131	0.914	1.587	0.907	5.550	5.278	4.496	5.178	2.209
ENSG00000141510	TP53	16.072	19.820	3.460	3.036	4.375	6.857	5.376	5.820	1.109	1.301	1.511
ENSG00000166889	PATL1	16.069	17.036	1.425	0.776	4.140	2.206	9.412	8.481	4.225	7.725	4.456
ENSG00000070669	ASNS	15.883	25.740	2.200	2.420	5.005	2.194	4.719	3.562	3.317	2.932	2.644
ENSG00000119707	RBM25	15.858	13.837	2.428	2.585	1.937	2.173	6.560	6.823	4.924	7.700	4.843
ENSG00000127946	HIP1	15.787	14.357	0.346	0.316	0.236	0.215	0.292	0.602	1.043	1.047	1.677
ENSG00000160214	RRP1	15.660	10.787	50.282	37.206	58.129	37.934	51.434	33.273	1.307	3.723	0.124
ENSG00000161980	POLR3K	15.624	12.424	91.502	128.669	52.177	59.478	37.543	27.180	12.677	4.787	6.321
ENSG00000177425	PAWR	15.620	15.318	0.131	0.183	0.177	0.217	0.275	0.745	1.682	2.081	0.501
ENSG00000116685	KIAA2013	15.595	15.527	5.638	5.321	2.071	3.417	1.471	2.464	4.140	4.525	5.120
ENSG00000125753	VASP	15.534	11.202	0.826	0.127	0.516	0.244	0.770	0.652	0.255	0.948	0.212
ENSG00000148468	FAM171A1	15.493	17.842	2.582	5.647	2.932	3.081	6.817	2.819	6.489	4.024	1.169
ENSG00000081181	ARG2	15.411	18.451	2.172	2.450	3.297	1.763	2.331	0.741	6.371	5.666	6.963
ENSG00000065268	WDR18	15.404	13.137	228.777	110.546	109.102	110.859	50.355	33.152	8.978	7.740	8.824
ENSG00000114346	ECT2	15.233	18.878	1.281	0.827	1.949	0.900	8.286	6.334	4.284	7.707	4.759
ENSG00000197043	ANXA6	15.192	15.958	3.214	1.780	4.161	2.720	6.251	5.348	2.844	5.800	3.724
ENSG00000182512	GLRX5	15.188	12.423	3.894	5.501	1.530	1.479	2.830	3.180	5.353	3.613	3.684
ENSG00000275832	ARHGAP23	14.995	14.882	0.136	0.026	0.073	0.058	0.014	0.038	1.501	2.232	0.377
ENSG00000138430	OLA1	14.741	13.851	2.650	4.698	3.207	3.311	3.392	3.909	6.564	5.975	4.780
ENSG00000067840	PDZD4	14.615	43.810	0.000	0.000	0.000	0.000	0.000	0.000	0.387	0.000	0.000
ENSG00000157110	RBPMS	14.479	10.861	0.021	0.000	0.098	0.024	0.036	0.080	3.338	1.697	3.344
ENSG00000111704	NANOG	14.358	14.786	0.000	0.000	0.013	0.000	0.051	0.145	0.000	0.000	0.000
ENSG00000213465	ARL2	14.158	17.260	294.789	213.371	78.058	126.118	37.464	20.939	7.058	9.903	14.567
ENSG00000116273	PHF13	14.147	15.048	2.093	2.093	4.131	1.516	7.628	5.148	5.431	8.563	3.839
ENSG00000184640	9-Sep	14.136	14.128	6.333	1.938	5.068	3.623	4.251	11.111	0.353	0.533	0.638
ENSG00000184640	9-Sep	14.136	14.128	2.333	1.938	2.068	3.623	4.251	3.111	0.353	0.533	0.638
ENSG00000073792	IGF2BP2	14.022	16.617	2.022	2.039	3.340	2.664	1.003	1.598	3.520	4.160	2.081
ENSG00000102755	FLT1	13.880	8.113	0.000	0.009	0.000	0.000	0.068	0.039	0.603	0.176	0.444
ENSG00000160211	G6PD	13.832	12.966	93.848	23.745	60.164	69.881	60.335	50.355	3.802	6.532	5.492
ENSG00000114739	ACVR2B	13.675	9.804	0.481	0.112	0.206	0.155	0.405	0.055	1.946	1.014	0.029
ENSG00000114739	ACVR2B	13.675	9.804	0.481	0.112	0.206	0.155	0.405	0.055	0.946	1.014	0.029
ENSG00000129625	REEP5	13.489	14.795	35.408	55.975	21.615	20.390	25.260	29.925	42.846	42.838	55.939
ENSG00000112996	MRPS30	13.484	12.805	9.293	14.322	22.295	17.478	25.241	32.484	1.297	5.266	3.569

ENSG00000154310	TNIK	13.416	9.809	0.125	0.092	0.312	0.069	0.338	0.327	0.665	0.383	0.616
ENSG00000111144	LTA4H	13.365	16.096	1.689	3.752	6.173	4.537	3.663	7.005	3.839	3.244	5.075
ENSG00000185483	ROR1	13.350	7.565	0.046	0.021	0.090	0.047	0.006	0.088	0.074	0.000	0.143
ENSG00000126247	CAPNS1	13.342	13.151	3.753	4.131	2.731	3.664	2.948	4.788	4.382	5.259	3.669
ENSG00000122965	RBM19	13.217	11.778	6.880	5.696	3.919	2.223	2.649	1.291	0.979	1.546	0.379
ENSG00000184983	NDUFA6	12.784	9.165	107.126	54.412	30.592	33.365	20.638	13.411	69.014	37.033	41.889
ENSG00000099364	FBXL19	12.577	10.796	0.180	0.069	0.029	0.113	0.023	0.027	0.072	0.018	0.000
ENSG00000181061	HIGD1A	12.558	14.136	46.664	59.357	66.116	45.782	122.997	33.504	96.255	126.519	121.074
ENSG00000182718	ANXA2	12.536	12.221	27.281	43.521	110.516	122.064	86.308	148.623	276.797	254.092	423.165
ENSG00000228474	OST4	12.464	10.751	177.270	70.818	64.798	77.259	27.301	49.781	254.896	185.174	130.691
ENSG00000212993	POU5F1B	12.248	11.341	56.633	57.427	302.003	139.565	193.987	185.760	0.000	0.000	0.407
ENSG00000107651	SEC23IP	12.234	11.934	1.483	1.108	2.664	1.400	2.910	2.891	4.178	7.768	2.072
ENSG00000143842	SOX13	12.217	12.749	0.036	0.041	0.206	0.154	0.097	0.417	0.000	0.249	0.000
ENSG00000138759	FRAS1	11.939	7.013	0.009	0.000	0.125	0.012	0.003	0.236	0.000	0.346	0.161
ENSG00000184344	GDF3	11.864	22.779	87.971	362.308	104.605	632.149	36.769	181.142	0.000	0.000	0.977
ENSG00000204065	TCEAL5	11.803	23.582	4.386	3.210	2.230	0.717	1.102	0.000	0.000	0.000	0.000
ENSG00000103449	SALL1	11.756	17.214	0.881	0.477	0.473	0.341	1.120	2.235	2.217	0.978	0.000
ENSG00000108651	UTP6	11.617	11.264	2.633	2.643	9.001	4.449	23.062	6.456	9.059	8.061	5.007
ENSG00000136383	ALPK3	11.576	11.663	0.020	0.079	0.080	0.000	0.043	0.028	0.650	0.075	0.000
ENSG00000071051	NCK2	11.548	11.125	2.128	2.393	2.098	2.610	2.497	3.208	2.700	4.228	3.352
ENSG00000175215	CTDSP2	11.445	15.427	1.419	1.768	1.615	1.211	2.173	2.696	6.536	5.265	6.306
ENSG00000175215	CTDSP2	11.445	15.427	1.419	1.768	1.615	1.211	2.173	2.696	6.536	5.265	6.306
ENSG00000137642	SORL1	11.281	10.638	0.039	0.011	0.249	0.037	0.058	0.055	0.391	0.268	0.000
ENSG00000138685	FGF2	11.235	9.598	0.138	0.143	0.117	0.241	0.348	0.255	3.411	5.392	10.038
ENSG00000090905	TNRC6A	11.176	9.818	1.137	0.684	0.531	0.531	2.092	2.481	2.897	1.897	2.458
ENSG00000080345	RIF1	11.150	11.703	0.736	0.392	0.575	0.528	3.614	2.771	4.070	3.936	4.540
ENSG00000110108	TMEM109	10.945	8.682	41.193	21.926	59.326	44.116	44.631	29.918	3.248	5.177	3.881
ENSG00000017483	SLC38A5	10.917	7.101	77.060	123.750	120.142	138.908	53.566	53.966	3.397	2.208	6.743
ENSG00000168280	KIF5C	10.715	14.569	0.000	0.000	0.000	0.000	0.020	0.000	0.499	1.237	0.000
ENSG00000165804	ZNF219	10.449	8.961	0.726	0.407	0.185	0.173	0.069	0.104	0.051	0.181	0.131
ENSG00000156453	PCDH1	10.416	8.562	0.000	0.007	0.040	0.047	0.000	0.010	0.000	0.112	0.353
ENSG00000182866	LCK	10.320	10.373	0.000	0.014	0.000	0.000	0.000	0.000	0.000	0.674	0.000
ENSG00000213079	SCAF8	10.273	10.496	1.107	0.960	0.847	0.593	2.845	3.789	4.531	4.335	1.288
ENSG00000105647	PIK3R2	10.235	9.510	0.112	0.064	0.011	0.060	0.009	0.016	1.383	0.043	1.248
ENSG00000119402	FBXW2	10.217	10.671	1.617	1.477	2.437	2.221	3.019	5.015	3.928	2.394	4.073

ENSG00000157106	SMG1	10.115	9.437	0.309	0.377	0.313	0.323	1.335	1.380	2.195	2.531	2.709
ENSG00000169783	LINGO1	10.095	8.009	0.032	0.049	0.070	0.018	0.034	0.018	0.000	0.000	0.000
ENSG00000139800	ZIC5	10.007	7.919	0.094	0.054	0.038	0.040	0.104	0.013	0.047	0.180	0.470
ENSG00000077782	FGFR1	10.002	10.558	0.038	0.050	0.083	0.102	0.062	0.095	0.603	0.156	1.960
ENSG00000136270	TBRG4	9.973	10.016	29.204	18.315	36.615	30.440	25.088	21.413	1.374	4.108	2.604
ENSG00000119772	DNMT3A	9.763	8.814	0.549	0.234	0.591	0.381	1.210	1.146	0.886	1.505	1.632
ENSG00000156574	NODAL	9.743	6.975	0.000	0.000	0.087	0.000	0.000	0.000	0.000	0.000	0.000
ENSG00000130396	MLLT4	9.666	8.161	0.142	0.081	0.112	0.071	0.126	0.166	0.494	0.638	1.333
ENSG00000163530	DPPA2	9.638	14.128	0.079	1.627	1.088	0.605	0.744	2.040	2.535	1.235	0.000
ENSG00000142449	FBN3	9.443	8.897	0.000	0.000	0.401	0.000	0.011	0.000	0.000	0.140	0.000
ENSG00000168894	RNF181	9.404	8.393	185.165	187.149	32.401	73.516	16.348	18.445	77.695	43.613	69.347
ENSG00000189043	NDUFA4	9.301	10.075	101.857	213.938	101.487	129.535	42.445	48.342	147.877	84.935	96.262
ENSG00000237515	SHISA9	9.186	5.241	0.046	0.000	0.030	0.000	0.023	0.000	0.000	0.185	0.000
ENSG00000143494	VASH2	9.117	25.538	0.036	0.050	0.036	0.100	0.096	0.070	0.000	0.000	0.000
ENSG00000168495	POLR3D	8.958	10.067	7.580	7.404	7.471	5.598	15.315	9.866	4.377	3.717	2.194
ENSG00000007062	PROM1	8.829	4.990	0.008	0.018	0.190	0.047	0.069	0.202	0.110	0.107	0.000
ENSG00000125966	MMP24	8.711	9.153	0.247	0.000	0.060	0.063	0.000	0.181	1.489	0.076	0.000
ENSG00000130707	ASS1	8.556	17.685	51.903	32.564	36.857	38.831	48.365	40.894	1.950	3.303	0.108
ENSG00000099954	CECR2	8.459	7.661	0.075	0.023	0.099	0.017	0.013	0.004	0.027	0.332	0.000
ENSG00000168610	STAT3	8.354	8.759	12.895	5.616	19.201	12.478	40.649	36.477	3.933	5.113	6.421
ENSG00000067141	NEO1	8.275	6.446	0.147	0.064	0.171	0.054	0.040	0.343	0.973	1.438	2.900
ENSG00000092758	COL9A3	8.247	11.829	0.086	0.028	0.060	0.010	0.062	0.007	0.467	0.037	0.136
ENSG00000100348	TXN2	8.242	8.166	105.839	125.091	67.321	75.552	46.554	40.924	18.990	14.411	12.766
ENSG00000279483	AC090498.1	7.956	6.002	85.162	23.950	63.514	42.968	36.075	27.421	129.757	115.544	101.762
ENSG00000151276	MAGI1	7.836	5.930	0.120	0.162	0.058	0.084	0.062	0.202	0.942	1.167	2.107
ENSG00000146701	MDH2	7.675	5.069	279.075	342.434	317.309	319.776	156.990	96.665	38.635	42.319	38.624
ENSG00000166833	NAV2	7.629	4.228	0.017	0.000	0.057	0.000	0.018	0.039	0.245	0.329	0.452
ENSG00000046889	PREX2	7.547	6.949	0.000	0.010	0.063	0.000	0.005	0.161	0.000	0.013	0.000
ENSG00000185201	IFITM2	7.274	6.330	126.344	231.805	26.496	237.967	36.158	42.067	125.032	50.134	105.869
ENSG00000088832	FKBP1A	7.171	7.862	14.638	39.254	43.894	32.222	28.731	45.723	45.866	43.153	63.707
ENSG00000001617	SEMA3F	6.868	6.109	0.000	0.000	0.009	0.019	0.000	0.038	0.000	0.000	0.000
ENSG00000105676	ARMC6	6.839	3.587	7.932	3.356	6.829	4.393	8.109	4.829	2.319	1.177	2.793
ENSG00000101680	LAMA1	6.820	7.152	0.051	0.065	0.123	0.014	0.050	0.291	0.611	0.577	0.350
ENSG00000142669	SH3BGRL3	6.705	8.251	233.894	235.384	103.660	368.564	69.170	120.606	703.592	266.502	546.296
ENSG00000163694	RBM47	6.501	5.454	0.011	0.046	0.494	0.005	0.504	0.163	0.514	0.452	0.063

ENSG00000197119	SLC25A29	6.372	6.900	0.410	0.174	0.193	0.276	0.299	0.236	0.071	0.449	0.292
ENSG00000166595	FAM96B	6.358	4.506	141.636	103.708	28.019	45.358	19.994	16.351	45.462	31.432	31.396
ENSG00000182944	EWSR1	6.349	5.578	17.508	18.257	60.922	40.076	40.648	38.851	13.834	7.207	14.544
ENSG00000116852	KIF21B	6.274	7.122	0.040	0.027	0.026	0.007	0.000	0.013	0.032	1.052	0.000
ENSG00000214706	IFRD2	6.167	4.971	26.893	14.749	43.703	37.263	29.244	22.446	2.408	3.814	4.679
ENSG00000096092	TMEM14A	6.153	5.540	20.766	71.809	51.493	78.580	51.526	105.581	167.222	118.178	149.670
ENSG00000148143	ZNF462	6.012	5.048	0.000	0.000	0.202	0.009	0.000	0.000	0.510	0.158	0.218
ENSG00000243449	C4orf48	5.950	6.293	457.028	298.387	59.286	144.371	33.839	45.413	124.936	153.178	102.655
ENSG00000133773	CCDC59	5.799	5.400	5.114	6.376	5.994	3.559	10.360	5.166	10.386	25.548	28.336
ENSG00000242616	GNG10	5.781	6.399	16.141	10.838	12.841	15.098	32.257	50.669	96.119	46.304	102.828
ENSG00000105607	GCDH	5.688	5.440	25.481	51.013	15.755	13.524	12.039	7.184	0.374	1.292	0.477
ENSG00000161960	EIF4A1	5.595	4.779	91.771	247.269	230.902	216.661	86.904	63.208	36.157	21.390	27.483
ENSG00000158716	DUSP23	5.492	4.894	161.175	128.961	35.631	69.600	19.585	19.299	101.019	32.968	56.257
ENSG00000107874	CUEDC2	5.426	3.717	125.948	75.283	33.833	47.746	30.101	36.598	32.579	30.294	34.868
ENSG00000182054	IDH2	5.150	4.270	96.778	185.653	96.864	178.228	85.440	47.733	28.531	18.293	21.350
ENSG00000165912	PACSIN3	5.080	5.679	19.045	7.504	9.922	11.478	11.200	10.693	0.725	0.174	0.042
ENSG00000183207	RUVBL2	4.990	5.860	53.506	33.386	59.518	23.186	18.866	88.914	8.980	19.781	21.363
ENSG00000105669	COPE	4.925	5.706	258.929	230.316	95.840	164.247	42.589	37.736	23.371	38.458	37.151
ENSG00000115446	UNC50	4.899	5.265	3.169	3.393	6.530	4.135	10.616	9.002	17.796	23.199	19.786
ENSG00000116586	LAMTOR2	4.872	3.521	165.168	208.972	38.343	73.471	22.117	22.458	41.648	28.281	45.148
ENSG00000185475	TMEM179B	4.843	4.360	146.555	111.060	42.437	74.754	34.927	32.712	63.657	31.756	56.045
ENSG00000135587	SMPD2	4.836	4.732	19.681	12.461	11.646	12.159	14.113	10.918	1.872	1.677	1.102
ENSG00000165688	PMPCA	4.782	7.622	63.345	61.639	64.532	54.447	49.721	36.281	3.565	9.963	3.503
ENSG00000117410	ATP6V0B	4.730	3.568	77.100	92.062	58.024	64.627	22.804	19.930	35.800	30.934	45.499
ENSG00000120509	PDZD11	4.716	5.956	45.632	104.223	29.015	37.920	30.103	25.995	84.089	36.703	41.372
ENSG00000184840	TMED9	4.680	3.471	105.631	87.928	99.460	51.158	51.975	73.911	11.656	9.361	7.321
ENSG00000130724	CHMP2A	4.628	5.730	56.008	66.504	28.021	54.047	24.406	26.686	44.732	37.920	62.799
ENSG00000113387	SUB1	4.614	5.977	22.866	52.330	47.711	29.657	33.285	31.287	33.450	32.716	49.119
ENSG00000115514	TXNDC9	4.594	3.343	14.061	15.037	18.873	16.092	33.338	24.365	24.023	28.447	24.178
ENSG00000100387	RBX1	4.537	3.461	108.904	200.750	57.292	67.266	25.405	21.550	64.803	44.107	55.679
ENSG00000138111	MFSD13A	4.476	2.631	11.010	6.523	11.809	8.115	11.938	7.750	0.000	0.000	0.234
ENSG00000100285	NEFH	4.467	4.152	35.986	45.952	61.780	32.285	158.673	56.248	4.750	3.482	0.704
ENSG00000122034	GTF3A	4.461	5.021	10.076	19.864	47.522	26.799	55.119	44.609	29.355	36.057	51.439
ENSG00000166136	NDUFB8	4.143	4.840	760.148	1460.948	179.020	417.361	63.309	53.240	77.614	41.679	59.823
ENSG00000185909	KLHDC8B	4.093	3.016	146.149	63.589	92.318	142.080	80.827	72.270	1.134	1.844	0.798

ENSG00000179091	CYC1	4.074	5.509	692.709	503.494	422.577	496.479	188.813	122.880	37.019	67.629	50.726
ENSG00000049323	LTBP1	4.073	5.417	0.286	0.155	0.278	0.244	0.210	0.510	25.406	12.731	38.325
ENSG00000173418	NAA20	4.046	4.336	21.238	44.565	47.559	29.810	46.040	33.950	37.302	40.800	47.991
ENSG00000232388	LINC00493	3.925	3.820	43.723	42.562	18.464	21.900	11.056	8.725	35.938	21.822	28.373
ENSG00000120697	ALG5	3.909	2.402	15.470	24.018	23.808	26.058	31.777	23.987	65.033	41.223	71.963
ENSG00000146425	DYNLT1	3.887	3.352	71.453	55.460	50.808	53.622	29.674	22.193	137.305	79.527	150.871
ENSG00000163382	APOA1BP	3.859	5.165	210.785	120.036	73.879	100.257	52.781	27.743	26.687	17.415	28.337
ENSG00000128463	EMC4	3.849	3.029	16.129	53.346	18.864	18.949	13.581	12.103	17.910	12.649	16.620
ENSG00000103274	NUBP1	3.828	2.925	10.854	20.581	12.918	8.657	18.341	7.938	17.457	16.223	25.417
ENSG00000197956	S100A6	3.805	1.361	233.648	174.800	56.695	480.537	46.521	191.314	751.503	413.639	341.657
ENSG00000129518	EAPP	3.786	2.380	16.776	30.530	19.486	13.164	29.695	27.737	25.894	37.486	30.883
ENSG00000102007	PLP2	3.775	3.503	123.344	73.256	79.385	88.758	50.197	56.148	167.020	79.318	200.812
ENSG00000130303	BST2	3.762	3.247	73.181	47.289	18.668	29.122	15.044	16.461	28.065	19.308	111.860
ENSG00000129559	NEDD8	3.750	2.956	97.425	118.883	29.209	50.818	17.180	22.101	28.992	26.879	29.465
ENSG00000105404	RABAC1	3.703	2.191	109.679	55.209	17.615	44.922	12.467	14.470	68.791	56.968	64.549
ENSG00000147155	EBP	3.702	2.990	53.007	57.238	43.116	26.802	31.293	17.937	32.795	15.315	31.868
ENSG00000164096	C4orf3	3.686	4.213	59.231	99.819	44.179	66.579	62.688	67.475	175.634	77.932	142.039
ENSG00000164405	UQCRQ	3.682	4.073	403.103	777.846	160.750	276.210	56.961	49.455	143.148	75.213	101.380
ENSG00000173486	FKBP2	3.582	3.331	86.909	53.861	20.930	34.046	18.361	16.073	45.532	43.649	55.395
ENSG00000125995	ROMO1	3.564	3.362	346.881	411.798	70.450	147.419	26.438	26.084	139.003	116.642	125.376
ENSG00000095380	NANS	3.539	3.925	27.884	27.666	26.762	13.701	28.320	16.962	32.732	23.002	28.231
ENSG00000075239	ACAT1	3.528	2.462	13.640	10.027	20.583	14.487	32.887	24.011	11.440	10.258	11.591
ENSG00000002586	CD99	3.513	4.703	33.757	41.696	53.893	34.066	48.525	46.116	134.214	106.436	85.223
ENSG00000188739	RBM34	3.498	2.453	14.949	12.141	14.732	12.552	30.239	24.054	22.512	20.564	22.180
ENSG00000135211	TMEM60	3.483	3.583	14.916	39.092	19.558	16.004	20.724	17.443	48.839	41.405	63.926
ENSG00000164919	COX6C	3.451	2.662	102.195	71.562	43.457	58.416	25.402	22.354	69.337	54.195	51.943
ENSG00000167775	CD320	3.441	3.367	224.512	112.315	101.254	124.557	47.073	28.942	20.299	16.974	15.827
ENSG00000161179	YDJC	3.438	2.974	62.277	23.439	25.028	33.347	19.836	16.681	1.303	1.907	1.256
ENSG00000171159	C9orf16	3.418	2.970	40.120	30.502	12.915	22.760	8.050	12.263	37.766	9.326	27.313
ENSG00000083444	PLOD1	3.412	3.279	12.303	15.190	19.513	17.936	26.458	39.758	21.996	31.372	46.512
ENSG00000065518	NDUFB4	3.382	3.840	46.973	34.665	33.075	37.730	16.143	18.911	35.812	20.111	31.297
ENSG00000196923	PDLIM7	3.313	2.742	29.792	15.486	21.242	25.562	16.170	35.217	17.615	32.444	22.154
ENSG00000230989	HSBP1	3.273	3.750	40.748	77.075	36.002	24.593	37.018	27.904	34.368	25.315	31.519
ENSG00000185608	MRPL40	3.270	3.428	117.547	121.415	45.570	60.152	35.483	25.648	44.192	27.784	29.341
ENSG00000143158	MPC2	3.267	3.508	20.247	58.078	32.718	20.260	16.142	10.875	68.790	53.806	59.258

ENSG00000168734	PKIG	3.254	3.057	11.863	25.042	16.266	11.196	13.419	11.503	24.130	18.950	45.278
ENSG00000105677	TMEM147	3.249	2.553	119.868	141.027	41.923	67.222	21.705	18.448	16.634	15.026	16.250
ENSG00000198937	CCDC167	3.232	2.192	334.737	357.352	63.214	83.553	32.919	22.587	85.317	39.333	71.606
ENSG00000105185	PDCD5	3.222	3.612	24.539	37.609	36.190	26.101	30.247	26.051	46.993	30.756	36.784
ENSG00000154096	CD90	3.186	3.661	53.823	66.722	57.564	210.217	17.233	150.464	8.140	13.858	8.961
ENSG00000164758	MED30	3.177	3.740	3.408	2.982	5.135	2.449	5.645	4.641	14.873	12.759	17.336
ENSG00000187608	ISG15	3.169	2.080	30.698	28.738	1.892	14.584	12.154	11.933	22.549	14.672	18.136
ENSG00000211450	C11orf31	3.142	2.865	66.638	34.901	21.367	22.386	12.067	12.612	37.926	22.656	31.333
ENSG00000119013	NDUFB3	3.141	3.927	114.837	42.599	51.208	23.180	15.370	12.945	76.676	53.829	52.225
ENSG00000101439	CST3	3.121	2.242	211.654	127.019	37.564	84.369	35.133	59.789	53.415	64.772	36.054
ENSG00000122873	CISD1	3.115	2.748	27.297	32.791	19.328	18.965	20.684	17.815	31.204	15.111	29.605
ENSG00000172172	MRPL13	3.112	3.808	19.364	66.693	35.371	24.788	29.512	16.577	19.963	20.411	32.102
ENSG00000105355	PLIN3	3.103	3.059	10.416	12.394	13.178	17.807	26.819	26.873	27.204	25.096	27.401
ENSG00000134248	LAMTOR5	3.097	4.839	122.933	159.117	32.447	64.680	20.588	33.970	75.296	28.129	26.379
ENSG00000181019	NQO1	3.090	2.467	14.202	36.266	28.403	21.048	71.241	47.306	53.037	101.867	37.667
ENSG00000075188	NUP37	3.086	3.247	10.057	12.741	12.439	10.796	11.749	9.663	19.370	12.480	17.294
ENSG00000183741	CBX6	3.059	3.555	66.897	79.015	40.810	51.911	71.008	82.682	4.465	7.641	9.547
ENSG00000184990	SIVA1	3.000	3.507	88.779	53.857	25.352	23.412	11.523	16.318	15.646	19.873	18.156
ENSG00000037241	RPL26L1	2.989	2.048	57.610	63.878	49.338	34.963	25.065	14.604	53.341	42.349	40.515
ENSG00000103507	BCKDK	2.989	2.456	46.246	13.968	34.294	30.508	25.186	17.246	12.521	10.192	13.605
ENSG00000150093	ITGB1	2.972	2.295	14.387	15.488	17.592	13.298	33.685	38.099	113.070	51.828	131.239
ENSG00000139233	LLPH	2.956	2.992	7.938	11.241	18.011	9.981	17.458	15.533	15.003	13.429	13.244
ENSG00000114902	SPCS1	2.935	2.231	12.640	26.566	22.086	18.946	21.889	19.429	45.007	34.656	43.372
ENSG00000099624	ATP5D	2.933	1.110	108.776	51.348	43.273	52.280	24.344	23.090	15.197	16.308	13.861
ENSG00000109390	NDUFC1	2.890	2.209	59.994	46.671	30.872	34.255	14.708	21.191	47.334	25.931	41.208
ENSG00000170043	TRAPPC1	2.877	2.671	66.312	44.737	18.985	32.865	13.137	25.913	32.545	27.877	67.347
ENSG00000137806	NDUFAF1	2.865	1.557	15.539	51.607	18.532	13.757	19.338	14.043	11.132	24.526	27.188
ENSG00000198818	SFT2D1	2.862	2.957	15.132	19.340	22.354	11.552	19.258	16.451	24.003	19.435	12.455
ENSG00000277586	NEFL	2.856	3.027	17.698	12.868	39.303	15.247	107.576	21.056	0.000	0.724	0.136
ENSG00000174886	NDUFA11	2.852	3.279	99.122	114.022	37.120	68.154	17.288	14.996	62.088	36.682	27.218
ENSG00000054148	PHPT1	2.846	2.738	189.182	205.823	42.468	76.856	24.312	20.141	53.106	24.135	37.082
ENSG00000138175	ARL3	2.824	2.216	11.229	23.896	9.788	8.857	12.298	10.449	37.739	21.604	17.958
ENSG00000071462	WBSCR22	2.809	2.149	16.494	66.179	25.580	23.826	22.835	20.463	17.854	23.628	24.884
ENSG00000141232	TOB1	2.808	2.121	10.888	12.317	10.832	13.895	36.587	54.353	11.853	25.714	15.372
ENSG00000167700	MFSD3	2.805	1.260	52.811	17.309	13.069	15.949	10.404	13.422	0.586	1.857	0.593

ENSG00000163528	CHCHD4	2.805	1.428	17.918	58.300	28.448	21.957	36.028	18.412	12.448	11.483	10.728
ENSG00000106400	ZNHIT1	2.804	2.968	46.669	81.961	19.539	34.565	14.243	17.438	30.836	20.834	25.400
ENSG00000109534	GAR1	2.797	2.672	12.580	14.743	16.102	14.813	22.176	12.923	3.922	5.314	61.327
ENSG00000259112	NDUFC2-KCTD1	2.789	1.704	64.259	133.754	38.428	35.924	14.336	10.411	40.474	22.608	40.217
ENSG00000177954	RPS27	2.777	3.065	49.557	36.258	16.469	21.428	11.243	12.437	41.207	44.350	40.503
ENSG00000069509	FUNDC1	2.773	2.916	8.095	19.474	15.514	12.327	22.781	23.784	17.020	17.648	20.811
ENSG00000276023	DUSP14	2.769	2.082	14.471	20.829	30.169	20.281	39.590	29.464	39.617	51.390	52.683
ENSG00000170430	MGMT	2.756	2.323	53.996	30.412	11.075	21.093	12.106	8.463	21.642	11.990	9.696
ENSG00000135046	ANXA1	2.756	2.611	106.758	62.567	94.641	116.828	143.724	287.716	222.120	110.140	211.479
ENSG00000114125	RNF7	2.746	2.810	12.833	11.322	12.543	10.433	13.682	11.483	28.066	23.168	25.483
ENSG00000087074	PPP1R15A	2.738	2.820	9.324	13.472	10.659	12.436	16.755	25.452	24.942	31.987	14.935
ENSG00000065833	ME1	2.730	1.709	12.554	14.226	18.719	14.967	42.700	46.659	15.926	31.462	26.944
ENSG00000086504	MRPL28	2.705	3.363	200.302	230.807	81.310	118.108	35.066	38.798	13.308	19.770	18.526
ENSG00000124172	ATP5E	2.704	2.506	45.093	63.784	31.455	39.533	16.050	20.042	85.030	55.787	65.178
ENSG00000107175	CREB3	2.682	2.621	20.554	16.111	10.704	13.523	12.828	9.265	16.279	17.915	15.612
ENSG00000120333	MRPS14	2.671	2.761	11.364	33.289	15.964	26.472	17.047	14.659	17.376	20.766	5.477
ENSG00000139505	MTMR6	2.654	1.950	10.020	9.752	8.701	9.855	12.023	12.539	7.102	12.481	15.214
ENSG00000269028	MTRNR2L12	2.652	2.749	107.957	13.462	64.982	126.096	22.368	35.600	69.354	92.203	48.257
ENSG00000148335	NTMT1	2.646	2.179	48.668	31.057	24.878	20.819	16.005	10.610	11.809	18.398	17.410
ENSG00000101187	SLCO4A1	2.644	3.857	23.184	15.102	21.522	24.692	17.571	25.445	0.000	0.792	1.151
ENSG00000101558	VAPA	2.626	2.891	16.594	23.327	18.590	12.290	29.301	25.875	27.294	38.666	31.016
ENSG00000126254	RBM42	2.594	3.232	94.945	30.406	74.469	66.816	49.837	40.978	15.686	13.848	16.235
ENSG00000221821	C6orf226	2.589	2.732	44.823	17.659	10.292	9.100	8.847	10.888	26.201	23.564	16.235
ENSG00000175768	TOMM5	2.581	2.475	53.395	64.586	33.754	38.929	16.898	11.070	42.607	17.990	25.341
ENSG00000174807	CD248	2.578	1.440	19.132	13.270	11.663	14.073	74.322	142.707	50.543	70.342	63.818
ENSG00000071553	ATP6AP1	2.557	2.278	13.435	10.954	22.198	12.045	28.853	36.323	9.296	11.759	18.747
ENSG00000175324	LSM1	2.555	2.996	9.295	26.194	9.609	13.660	10.242	13.433	20.312	32.677	31.942
ENSG00000188643	S100A16	2.553	2.094	50.232	85.345	20.710	222.762	13.626	103.310	180.020	60.802	75.325
ENSG00000204922	UQCC3	2.530	1.542	91.521	118.362	20.549	25.303	19.623	16.979	25.147	13.821	14.698
ENSG00000157916	RER1	2.523	2.749	36.226	57.021	26.620	25.832	30.982	19.312	32.753	29.895	33.031
ENSG00000175550	DRAP1	2.517	2.568	38.763	27.492	12.740	18.459	10.282	14.009	56.340	63.968	78.257
ENSG00000142227	EMP3	2.507	2.320	152.718	137.533	22.112	138.410	17.953	27.351	114.812	56.529	85.993
ENSG00000204525	HLA-C	2.489	1.232	10.004	13.019	13.728	15.480	12.097	37.918	20.366	27.108	27.711
ENSG00000140264	SERF2	2.488	2.497	251.457	235.258	81.854	134.656	28.230	32.597	77.875	38.785	60.307
ENSG00000162076	FLYWCH2	2.480	2.184	49.775	51.768	25.880	25.488	32.861	17.420	10.401	28.303	23.167

ENSG00000140307	GTF2A2	2.433	2.064	10.696	24.933	15.860	11.676	16.180	9.680	21.838	17.348	20.222
ENSG00000267855	NDUFA7	2.411	2.513	111.410	106.099	36.148	47.952	19.325	18.223	31.735	21.270	25.315
ENSG00000164647	STEAP1	2.403	2.826	0.000	0.219	0.621	0.163	0.271	1.452	130.846	89.412	84.948
ENSG00000234745	HLA-B	2.403	1.671	14.992	11.645	13.219	50.901	12.387	94.501	92.634	87.462	82.782
ENSG00000168090	COPS6	2.393	3.931	33.271	17.277	36.657	17.914	34.637	26.622	27.987	30.804	40.854
ENSG00000241370	RPP21	2.392	1.292	37.610	28.120	10.290	7.452	8.229	7.648	18.020	13.889	15.370
ENSG00000120306	CYSTM1	2.376	2.462	28.690	56.315	17.279	24.846	14.325	13.494	53.335	53.426	54.901
ENSG00000099817	POLR2E	2.372	2.415	210.169	78.312	81.521	110.373	34.070	29.656	17.553	14.666	18.456
ENSG00000147955	SIGMAR1	2.370	1.298	55.526	41.938	70.853	55.805	49.561	43.919	6.961	9.086	7.461
ENSG00000148175	STOM	2.369	1.423	13.816	19.140	43.921	20.444	123.922	129.580	14.631	19.286	12.952
ENSG00000174851	YIF1A	2.364	2.438	112.653	76.327	33.020	64.232	20.717	20.825	34.097	35.510	43.201
ENSG00000163453	IGFBP7	2.362	3.282	1.007	0.547	0.775	0.136	0.567	0.954	143.817	74.017	110.330
ENSG00000165898	ISCA2	2.341	2.123	21.076	19.747	12.656	10.496	10.292	12.250	10.952	13.612	17.814
ENSG00000175606	TMEM70	2.336	2.297	6.172	10.110	11.231	6.978	12.677	9.730	8.501	10.792	14.562
ENSG00000143314	MRPL24	2.324	3.166	199.486	200.413	60.774	88.933	47.289	33.200	12.436	19.996	40.682
ENSG00000228300	C19orf24	2.299	2.218	45.549	37.221	13.260	18.362	9.247	8.725	34.404	29.261	23.976
ENSG00000159479	MED8	2.299	2.298	10.769	24.076	11.846	10.449	13.380	11.985	19.532	23.782	29.600
ENSG00000125037	EMC3	2.289	2.113	26.677	99.881	30.237	35.709	29.772	28.753	40.609	32.683	39.402
ENSG00000219200	RNASEK	2.287	1.251	22.637	13.331	9.542	10.929	12.142	11.562	27.885	14.900	19.772
ENSG00000123136	DDX39A	2.284	3.076	46.875	69.855	41.667	31.394	22.584	11.938	12.134	10.997	14.854
ENSG00000149328	GLB1L2	2.274	1.522	4.465	1.955	8.247	4.922	11.660	12.927	0.045	0.566	0.374
ENSG00000116521	SCAMP3	2.263	5.525	186.026	258.187	70.020	105.127	43.463	28.807	11.056	19.969	18.163
ENSG00000151239	TWF1	2.259	1.347	6.051	8.126	8.775	10.428	16.369	12.319	20.217	25.924	17.120
ENSG00000262664	OVCA2	2.255	2.202	16.029	14.257	10.178	9.203	18.989	7.815	27.148	16.200	76.850
ENSG00000173530	TNFRSF10D	2.249	2.360	6.711	6.155	12.686	8.233	53.270	38.963	35.118	17.157	11.775
ENSG00000074319	TSG101	2.243	2.822	8.364	9.480	8.088	12.675	13.716	12.827	15.091	22.740	26.621
ENSG00000170891	CYTL1	2.243	1.058	10.135	29.712	21.639	25.638	58.600	91.640	0.000	0.000	0.135
ENSG00000196792	STRN3	2.234	3.227	8.580	13.702	8.114	9.190	52.438	34.248	16.373	31.046	23.186
ENSG00000125863	MKKS	2.232	2.005	11.206	12.912	11.880	9.930	16.426	16.817	16.764	11.825	13.205
ENSG00000270757	HSPE1-MOB4	2.226	2.594	11.634	19.658	14.261	12.539	12.632	12.747	18.713	15.927	30.208
ENSG00000168275	COA6	2.181	1.703	14.594	21.019	12.725	10.215	16.746	10.448	19.907	13.385	31.988
ENSG00000165264	NDUFB6	2.178	2.801	143.358	195.531	86.194	75.818	63.182	34.783	87.819	83.726	94.328
ENSG00000215472	RPL17-C18orf32	2.161	2.382	12.149	19.324	30.192	29.663	34.272	29.538	2.533	58.318	82.719
ENSG00000118939	UCHL3	2.159	2.344	16.515	36.650	24.233	14.814	20.652	13.173	39.621	19.371	34.333
ENSG00000171421	MRPL36	2.150	1.644	29.693	125.803	17.255	54.322	10.131	15.325	42.225	21.361	34.064

ENSG00000204520	MICA	2.147	2.951	17.576	10.709	13.284	17.106	10.497	16.847	41.828	29.623	57.723
ENSG00000070010	UFD1L	2.140	2.377	10.361	20.593	13.893	8.416	13.217	9.125	17.389	13.467	16.300
ENSG00000198225	FKBP1C	2.102	3.884	12.425	24.377	38.237	27.924	23.724	35.690	31.503	33.162	36.354
ENSG00000123353	ORMDL2	2.098	2.402	24.496	33.581	10.880	10.290	10.878	10.052	19.320	17.745	16.087
ENSG00000111237	VPS29	2.095	2.433	24.586	32.660	14.485	15.802	13.934	10.441	39.858	31.588	38.424
ENSG00000105825	TFPI2	2.069	3.401	26.243	36.787	25.863	27.950	173.476	101.203	175.685	35.334	74.391
ENSG00000141378	PTRH2	2.056	1.284	12.879	30.493	9.014	10.072	10.064	8.307	9.112	10.086	7.000
ENSG00000129055	ANAPC13	2.055	1.535	11.625	8.342	19.160	12.224	27.248	11.985	18.416	19.380	19.425
ENSG00000126756	UXT	2.049	2.640	38.395	37.440	11.148	11.752	9.317	10.965	53.927	27.793	28.008
ENSG00000092010	PSME1	2.045	2.893	26.666	32.260	15.297	17.587	18.429	12.763	23.799	18.809	19.658
ENSG00000181649	PHLDA2	2.044	2.079	507.432	386.563	161.668	545.363	97.202	174.974	254.242	217.924	487.204
ENSG00000115944	COX7A2L	2.017	3.980	12.821	17.468	19.812	16.933	29.053	10.098	17.726	39.692	42.353
ENSG00000128626	MRPS12	2.012	5.009	96.151	60.283	240.482	84.194	113.551	81.150	24.949	19.552	18.982
ENSG00000085231	AK6	1.974	2.588	8.980	16.680	9.021	10.052	19.019	10.797	18.167	15.109	18.775
ENSG00000162704	ARPC5	1.944	1.971	13.838	26.237	51.635	22.397	93.815	84.389	30.839	29.962	42.671
ENSG00000112667	DNPH1	1.943	1.634	294.254	89.750	49.938	82.090	30.302	27.940	19.987	14.593	12.041
ENSG00000100142	POLR2F	1.925	1.147	48.992	46.338	18.791	29.192	9.207	18.331	18.041	12.342	15.736
ENSG00000135441	BLOC1S1	1.925	2.606	48.105	44.992	10.557	16.156	12.225	15.623	45.020	14.644	30.060
ENSG00000167792	NDUFV1	1.911	1.019	51.033	51.048	45.525	50.097	30.718	23.131	13.881	8.669	7.591
ENSG00000163463	KRTCAP2	1.910	1.371	23.512	20.284	8.746	13.164	10.261	12.076	20.731	19.084	22.682
ENSG00000084072	PPIE	1.909	1.821	29.277	32.973	11.636	15.420	9.231	10.166	12.182	10.432	15.929
ENSG00000105619	TFPT	1.908	2.003	93.896	90.875	13.550	18.964	11.788	19.237	31.021	17.453	30.184
ENSG00000105364	MRPL4	1.905	1.666	35.208	68.066	65.593	84.605	42.630	21.418	15.270	9.495	14.345
ENSG00000100319	ZMAT5	1.899	1.015	43.670	37.377	11.122	15.140	7.254	5.573	15.425	16.939	17.193
ENSG00000205155	PSENEN	1.881	2.176	65.282	31.607	22.077	25.560	11.737	11.377	29.991	34.058	39.129
ENSG00000160049	DFFA	1.880	2.746	105.998	92.675	40.923	51.735	49.279	26.019	6.207	5.288	4.070
ENSG00000115520	COQ10B	1.860	1.559	10.705	12.035	13.190	10.738	43.934	27.310	25.383	15.422	15.668
ENSG00000141552	ANAPC11	1.851	1.505	32.891	19.380	7.748	12.615	13.138	7.293	11.623	9.010	23.182
ENSG00000128039	SRD5A3	1.833	1.158	10.675	17.581	10.134	10.003	16.448	9.098	10.306	11.569	18.811
ENSG00000105755	ETHE1	1.832	1.275	17.105	10.486	12.355	12.083	9.146	12.003	18.318	11.867	16.107
ENSG00000184743	ATL3	1.815	1.481	6.910	7.054	10.399	10.820	23.832	15.475	22.459	18.591	14.632
ENSG00000106628	POLD2	1.809	1.513	54.993	62.120	88.497	66.436	37.062	33.605	4.562	5.140	8.385
ENSG00000178695	KCTD12	1.808	1.122	5.743	5.441	6.716	13.482	5.797	6.383	18.904	8.947	15.330
ENSG00000214160	ALG3	1.805	1.816	45.700	62.030	29.165	43.423	22.069	25.882	15.754	17.009	15.948
ENSG00000171861	RNMTL1	1.791	2.037	33.910	92.584	46.423	36.940	45.878	32.457	5.629	9.048	15.868

ENSG00000099940	SNAP29	1.788	1.076	9.236	8.654	8.332	7.594	10.554	8.209	11.237	17.522	11.897
ENSG00000122958	VPS26A	1.788	1.615	12.095	10.217	11.237	8.927	23.612	13.899	21.785	48.961	26.475
ENSG00000183155	RABIF	1.785	1.769	17.718	23.143	10.966	11.714	13.214	10.271	20.741	32.117	19.337
ENSG00000188243	COMMD6	1.781	1.039	8.951	10.831	8.282	8.371	7.526	9.746	17.678	16.229	10.197
ENSG00000116209	TMEM59	1.779	1.029	13.514	9.977	9.076	14.197	15.324	20.481	31.449	35.362	41.123
ENSG00000257704	INAFM1	1.779	1.027	69.567	37.741	8.042	12.674	10.248	11.043	13.620	41.856	27.405
ENSG00000136783	NIPSNAP3A	1.777	1.806	15.957	10.740	12.256	10.998	25.090	27.783	14.971	12.233	15.328
ENSG00000198612	COPS8	1.764	1.625	15.354	10.493	12.351	11.327	16.577	28.465	41.280	21.593	23.763
ENSG00000156411	C14orf2	1.750	1.822	50.752	56.360	16.879	22.902	10.156	12.586	19.574	15.424	11.569
ENSG00000180879	SSR4	1.744	1.086	103.747	165.759	21.890	58.265	12.051	18.732	40.791	24.073	35.695
ENSG00000154277	UCHL1	1.742	1.918	39.650	65.513	87.411	62.796	100.225	68.478	110.415	129.179	74.544
ENSG00000172922	RNASEH2C	1.738	1.721	34.558	18.454	10.697	10.568	10.151	9.730	22.775	11.389	12.999
ENSG00000161939	RNASEK-C17orf4	1.724	1.244	12.392	12.621	10.273	8.418	10.945	8.721	8.534	10.888	14.358
ENSG00000150787	PTS	1.719	1.131	12.410	12.845	8.905	8.675	7.153	8.145	13.008	10.083	13.091
ENSG00000104671	DCTN6	1.694	1.690	9.870	14.506	13.638	11.589	18.429	19.304	35.623	27.211	38.491
ENSG00000151287	TEX30	1.691	1.980	13.111	8.451	9.568	8.401	11.684	10.836	101.555	103.437	194.123
ENSG00000173436	MINOS1	1.691	1.557	40.938	31.872	19.638	23.171	11.940	12.819	26.419	12.542	14.802
ENSG00000244509	APOBEC3C	1.690	3.874	344.215	247.893	75.793	109.171	49.710	44.293	11.036	19.534	18.196
ENSG00000023572	GLRX2	1.675	1.815	9.281	14.462	10.003	9.292	14.306	14.230	26.674	16.658	26.177
ENSG00000156603	MED19	1.673	1.959	11.353	9.092	8.989	12.573	12.174	8.409	15.370	27.713	30.109
ENSG00000010278	CD9	1.673	1.140	10.798	12.355	13.624	16.229	13.051	13.142	40.399	21.746	16.876
ENSG00000124380	SNRNP27	1.672	2.077	9.777	10.591	11.395	8.100	17.196	12.745	20.498	27.907	18.622
ENSG00000152377	SPOCK1	1.662	0.992	8.479	10.712	8.479	13.065	7.973	39.538	13.968	10.897	19.212
ENSG00000020129	NCDN	1.655	2.502	5.382	1.979	14.526	9.379	19.894	17.730	4.975	5.621	5.527
ENSG00000124786	SLC35B3	1.653	1.191	12.479	10.579	10.764	12.089	14.480	13.257	25.226	24.737	11.221
ENSG00000174109	C16orf91	1.628	1.054	48.754	30.313	20.759	21.625	22.766	16.235	16.465	22.958	12.947
ENSG00000169019	COMMD8	1.618	1.830	24.450	14.182	16.892	15.157	33.002	21.655	62.938	61.390	103.077
ENSG00000213523	SRA1	1.616	1.460	61.154	138.393	34.419	50.835	23.330	21.033	23.191	22.335	31.642
ENSG00000117054	ACADM	1.615	1.777	8.342	10.629	14.363	9.036	17.980	28.199	9.440	12.560	13.929
ENSG00000116096	SPR	1.602	2.681	16.168	19.979	14.372	21.617	15.887	24.003	4.257	6.321	8.782
ENSG00000120738	EGR1	1.599	2.108	99.514	12.885	14.726	12.895	19.131	14.637	31.774	21.204	36.972
ENSG00000184220	CMSS1	1.593	1.676	10.080	12.186	10.074	10.887	13.522	10.559	11.894	15.075	18.722
ENSG00000048162	NOP16	1.588	1.912	72.044	80.229	45.351	56.981	27.914	17.514	18.146	19.045	17.929
ENSG00000183605	SFXN4	1.575	1.717	15.273	24.917	21.859	18.374	27.444	16.258	6.435	4.529	7.849
ENSG00000183172	SMDT1	1.566	1.833	22.197	18.943	7.707	9.942	10.399	7.960	8.943	8.037	6.025

ENSG00000167565	SERTAD3	1.549	1.920	6.535	8.509	6.426	7.066	8.320	9.216	10.767	12.441	12.025
ENSG00000143198	MGST3	1.539	1.882	26.128	25.560	11.164	12.291	10.458	10.177	30.274	17.956	22.125
ENSG00000178718	RPP25	1.539	2.768	17.955	91.567	32.142	18.183	40.099	36.000	7.359	6.563	6.224
ENSG00000217930	PAM16	1.537	1.581	19.030	22.138	10.340	10.883	9.144	9.803	11.450	13.177	8.180
ENSG00000265972	TXNIP	1.521	1.211	13.364	12.214	12.680	12.234	15.024	37.214	19.653	42.189	16.518
ENSG00000159199	ATP5G1	1.515	1.874	346.980	353.077	93.033	185.739	35.077	31.564	29.231	18.128	21.661
ENSG00000106333	PCOLCE	1.510	1.395	21.008	12.160	17.200	39.796	14.074	74.095	25.872	30.176	39.141
ENSG00000142166	IFNAR1	1.509	1.299	8.342	9.766	10.113	8.979	16.276	12.218	9.625	11.329	8.405
ENSG00000215712	TMEM242	1.508	2.270	12.770	12.936	9.683	10.291	7.647	9.835	15.076	11.194	19.956
ENSG00000243147	MRPL33	1.506	1.941	19.912	10.958	13.326	12.720	13.286	10.060	49.720	32.048	32.654
ENSG00000111678	C12orf57	1.495	1.469	28.592	19.457	10.587	9.962	12.172	10.545	25.789	28.267	21.030
ENSG00000243279	PRAF2	1.485	2.311	43.150	58.408	33.946	79.938	46.592	34.877	61.552	30.968	38.486
ENSG00000163156	SCNM1	1.483	1.566	28.178	80.229	15.705	19.862	10.191	10.754	14.815	15.515	15.886
ENSG00000115738	ID2	1.479	1.167	5.071	5.423	6.306	5.377	6.388	5.981	71.332	25.939	41.496
ENSG00000130287	NCAN	1.477	0.612	10.837	7.212	8.088	8.119	26.144	10.957	0.000	0.000	0.000
ENSG00000187051	RPS19BP1	1.476	2.079	29.650	22.542	10.687	19.740	8.715	9.666	12.502	12.001	11.248
ENSG00000124570	SERPINB6	1.466	1.155	26.177	48.735	31.451	59.878	27.062	89.111	19.387	10.772	18.114
ENSG00000129235	TXNDC17	1.459	0.840	16.310	15.348	9.559	11.160	10.715	9.729	23.042	12.589	15.705
ENSG00000081870	HSPB11	1.448	1.986	16.091	13.837	11.811	12.954	12.490	14.679	30.548	17.383	31.227
ENSG00000256060	TRAPPC2P1	1.443	1.275	11.406	17.723	8.006	10.487	10.766	8.732	9.562	8.894	13.016
ENSG00000166710	B2M	1.429	1.723	21.509	115.938	26.978	76.283	20.930	73.951	65.852	129.617	355.572
ENSG00000155868	MED7	1.424	1.293	6.524	10.294	8.535	6.383	12.914	8.171	15.137	9.768	17.696
ENSG00000137100	DCTN3	1.422	1.096	35.867	34.474	8.290	11.487	6.642	9.769	20.069	8.164	12.057
ENSG00000165169	DYNLT3	1.417	1.669	10.877	10.043	15.678	12.602	26.299	13.673	67.081	36.343	34.663
ENSG00000175348	TMEM9B	1.406	1.314	12.983	8.417	10.969	12.225	20.611	21.004	21.318	12.337	12.170
ENSG00000130489	SCO2	1.398	1.719	105.622	71.315	47.585	55.047	37.902	26.306	13.965	19.980	10.761
ENSG00000197019	SERTAD1	1.398	1.594	17.840	8.927	9.380	8.624	12.625	8.267	25.293	28.714	29.101
ENSG00000187689	AMTN	1.397	0.000	29.957	261.560	50.364	44.125	13.634	91.490	0.000	0.000	0.582
ENSG00000108528	SLC25A11	1.397	1.150	47.009	35.048	52.387	15.191	58.533	40.237	15.489	11.781	8.037
ENSG00000213965	NUDT19	1.393	1.383	14.070	19.544	39.803	21.006	99.780	147.340	10.340	15.400	26.041
ENSG00000115756	HPCAL1	1.390	1.280	12.331	5.770	20.887	17.391	21.167	16.272	0.479	0.780	0.176
ENSG00000138777	PPA2	1.387	1.217	6.162	10.841	12.128	6.620	17.089	13.152	7.254	7.319	14.353
ENSG00000171202	TMEM126A	1.387	1.639	17.113	16.772	11.914	11.093	13.517	7.932	23.839	14.463	14.668
ENSG00000105671	DDX49	1.381	0.911	21.019	14.003	19.644	19.478	19.808	15.592	7.672	6.113	5.346
ENSG00000173163	COMMD1	1.381	1.182	23.222	45.427	11.556	17.184	9.032	11.896	21.089	13.429	18.541

ENSG00000171223	JUNB	1.377	0.926	47.012	50.631	47.474	27.293	40.864	99.158	24.683	58.183	31.141
ENSG00000169964	TMEM42	1.372	1.065	25.032	22.463	12.309	10.971	8.474	8.653	9.484	10.200	10.547
ENSG00000131435	PDLIM4	1.366	1.959	28.823	12.069	10.941	10.529	10.652	18.607	19.525	13.711	23.156
ENSG00000164414	SLC35A1	1.352	0.925	10.710	8.276	8.949	8.873	15.377	10.341	8.512	8.931	11.862
ENSG00000159231	CBR3	1.350	1.455	52.773	61.577	14.843	17.347	20.072	13.042	51.363	15.692	17.404
ENSG00000184164	CRELD2	1.343	1.074	20.884	19.233	14.379	21.727	15.276	12.722	10.081	12.710	11.745
ENSG00000104660	LEPROTL1	1.331	1.804	10.542	8.258	12.042	10.677	15.680	13.245	16.757	10.755	11.078
ENSG00000204822	MRPL53	1.325	1.744	27.916	21.662	8.542	10.965	8.373	8.026	10.901	10.926	29.370
ENSG00000105223	PLD3	1.323	1.051	58.667	7.692	60.198	47.484	32.276	63.313	13.741	5.340	8.448
ENSG00000140395	WDR61	1.320	0.663	10.634	8.001	10.544	12.877	11.663	9.304	10.101	10.954	13.982
ENSG00000177683	THAP5	1.320	1.188	8.200	13.957	8.215	10.977	19.791	23.535	13.447	13.741	23.037
ENSG00000063241	ISOC2	1.318	0.643	85.202	57.501	33.721	50.356	25.298	18.288	7.915	9.273	8.748
ENSG00000102471	NDFIP2	1.311	1.259	5.182	5.259	6.491	6.747	14.848	15.126	6.706	7.751	8.993
ENSG00000138495	COX17	1.310	1.568	26.068	29.055	10.897	9.023	8.993	13.104	26.105	13.092	9.544
ENSG00000108061	SHOC2	1.305	1.652	10.964	8.894	2.067	9.344	43.476	12.409	9.931	12.260	20.228
ENSG00000110711	AIP	1.300	1.734	262.564	226.294	39.743	69.290	36.607	22.852	18.312	14.459	15.043
ENSG00000134107	BHLHE40	1.300	1.606	10.772	12.861	8.971	8.772	8.403	47.728	11.362	13.508	13.648
ENSG00000108590	MED31	1.300	1.346	10.874	9.452	6.771	8.692	10.291	10.659	16.688	12.861	19.539
ENSG00000229833	PET100	1.298	1.140	52.199	47.678	12.340	13.541	10.626	10.689	12.326	7.296	9.146
ENSG00000143771	CNIH4	1.297	1.175	28.380	18.147	10.141	13.136	12.696	10.089	19.338	12.420	20.740
ENSG00000099246	RAB18	1.291	1.014	2.430	12.158	10.987	13.248	15.751	11.901	8.549	10.347	13.142
ENSG00000119333	WDR34	1.280	1.361	85.127	33.645	74.009	39.856	36.338	21.355	6.774	7.780	5.189
ENSG00000161638	ITGA5	1.268	1.315	10.872	9.735	12.091	12.445	10.739	10.864	45.615	27.883	45.192
ENSG00000158019	BRE	1.252	1.411	14.096	33.608	21.018	17.586	33.314	22.918	7.720	10.838	7.208
ENSG00000126088	UROD	1.248	1.184	59.199	117.378	38.091	67.501	19.586	20.980	10.048	14.607	12.104
ENSG00000204264	PSMB8	1.247	1.885	8.558	12.853	12.470	30.635	10.204	48.647	18.771	37.389	36.150
ENSG00000171453	POLR1C	1.246	0.930	23.872	66.041	19.906	21.765	18.919	15.459	12.251	10.288	8.532
ENSG00000217555	CKLF	1.240	0.846	32.790	51.151	10.071	12.937	15.497	41.797	19.545	18.277	17.850
ENSG00000105974	CAV1	1.235	1.173	20.376	14.720	13.617	11.063	33.443	30.869	33.726	17.013	64.206
ENSG00000162909	CAPN2	1.213	2.051	10.988	13.419	15.399	10.931	10.532	32.030	18.750	18.934	21.285
ENSG00000186340	THBS2	1.209	1.923	4.317	5.080	4.038	6.020	5.036	7.274	32.772	23.522	69.897
ENSG00000204316	MRPL38	1.202	1.764	28.746	19.584	38.628	20.453	22.532	16.966	5.181	5.390	8.730
ENSG00000180185	FAHD1	1.201	1.793	38.723	22.120	15.575	19.159	18.851	16.574	12.328	10.257	8.460
ENSG00000168701	TMEM208	1.201	0.927	43.170	53.276	10.967	14.324	9.919	10.681	15.280	12.093	14.898
ENSG00000159720	ATP6V0D1	1.196	1.467	15.244	9.389	12.442	14.428	9.929	12.454	11.399	14.204	10.828

ENSG00000179598	PLD6	1.184	0.027	6.685	9.617	23.654	12.245	48.294	17.199	9.929	33.417	43.048
ENSG00000181991	MRPS11	1.173	2.538	20.006	59.342	17.183	26.076	15.160	13.157	17.331	11.838	15.619
ENSG00000119408	NEK6	1.171	2.431	7.914	5.893	8.540	10.400	7.790	13.135	6.371	11.963	23.716
ENSG00000178057	NDUFAF3	1.167	1.834	192.753	141.997	36.935	57.222	26.335	18.553	42.771	24.976	39.375
ENSG00000243989	ACY1	1.161	1.684	58.360	48.088	23.755	32.941	22.434	20.190	13.996	16.876	15.872
ENSG00000153214	TMEM87B	1.161	1.560	10.317	9.078	8.436	9.124	6.418	10.996	13.237	12.981	13.711
ENSG00000100911	PSME2	1.158	1.280	30.598	66.099	18.643	30.401	19.934	19.054	25.471	19.641	21.340
ENSG00000172465	TCEAL1	1.147	1.211	12.007	8.925	11.967	10.927	14.379	8.357	25.102	12.162	14.274
ENSG00000110536	PTPMT1	1.144	1.825	14.887	14.541	8.827	8.440	8.923	9.026	8.296	7.211	11.699
ENSG00000140983	RHOT2	1.139	1.972	27.480	6.229	13.020	24.100	14.733	15.704	3.436	2.034	4.449
ENSG00000163683	SMIM14	1.137	1.403	8.684	7.753	8.768	6.352	9.327	8.527	16.973	15.006	13.820
ENSG00000115641	FHL2	1.137	1.662	10.919	17.787	14.409	10.788	17.416	9.681	13.790	16.509	15.265
ENSG00000012061	ERCC1	1.134	1.594	33.282	26.934	13.198	15.142	13.131	10.582	20.603	19.563	25.548
ENSG00000108961	RANGRF	1.132	1.208	32.742	23.639	13.567	16.766	11.142	6.749	11.468	11.858	17.508
ENSG00000112651	MRPL2	1.131	1.209	127.749	154.611	56.345	64.374	31.121	21.573	15.193	18.199	11.728
ENSG00000104823	ECH1	1.129	1.825	324.939	389.859	93.616	213.137	66.577	51.259	9.602	13.194	6.230
ENSG00000101883	MORN2	1.126	0.752	5.803	8.431	5.328	5.753	4.970	6.198	23.551	14.117	21.545
ENSG00000164885	CDK5	1.113	1.845	32.909	17.574	10.914	16.643	11.662	8.572	14.213	12.946	18.756
ENSG00000099377	HSD3B7	1.103	1.167	28.121	13.179	16.551	35.063	7.760	27.989	0.176	0.000	0.000
ENSG00000070495	JMJD6	1.098	1.885	6.898	9.604	6.275	5.538	10.200	6.355	5.357	9.344	8.726
ENSG00000064601	CTSA	1.087	1.822	34.886	29.503	16.742	18.190	16.676	17.525	18.365	18.175	16.537
ENSG00000118363	SPCS2	1.085	0.965	10.489	24.121	12.471	10.231	15.234	13.475	13.799	10.684	11.677
ENSG00000051108	HERPUD1	1.082	1.547	6.855	8.447	12.773	9.072	12.410	9.322	14.845	25.820	19.319
ENSG00000169189	NSMCE1	1.082	0.706	9.771	20.995	10.584	10.844	12.323	8.263	9.858	10.225	17.048
ENSG00000090266	NDUFB2	1.078	1.233	84.934	137.462	33.977	65.200	13.355	12.037	32.426	19.726	22.869
ENSG00000198832	SELM	1.073	1.414	15.599	12.527	12.169	12.615	12.767	9.545	76.013	37.767	59.665
ENSG00000087884	AAMDC	1.071	1.388	13.039	12.312	8.053	12.497	10.824	11.470	15.020	12.951	21.904
ENSG00000119878	CRIPT	1.055	1.329	19.703	21.613	11.797	9.216	14.509	10.978	24.630	25.845	21.049
ENSG00000122378	FAM213A	1.054	1.455	6.448	10.883	15.534	5.634	28.213	14.166	5.852	6.903	4.695
ENSG00000128283	CDC42EP1	1.051	1.181	29.797	5.704	45.738	27.174	38.968	55.163	10.838	12.755	10.976
ENSG00000107872	FBXL15	1.048	1.527	66.738	28.228	11.808	16.702	11.436	7.302	6.007	12.176	10.302
ENSG00000005486	RHBDD2	1.044	2.712	22.966	10.690	14.964	14.989	17.085	23.698	8.472	10.047	10.871
ENSG00000155957	TMBIM4	1.024	0.941	3.571	4.057	3.721	3.635	6.499	6.124	12.109	6.599	8.939
ENSG00000177556	ATOX1	1.009	0.533	55.006	77.204	9.851	19.439	10.237	16.262	24.753	15.961	21.259
ENSG00000131981	LGALS3	1.005	0.797	13.140	10.513	13.836	11.326	16.062	41.371	97.764	77.910	59.746

ENSG00000066322	ELOVL1	1.002	1.870	26.416	58.763	12.873	16.698	8.802	6.273	8.560	13.988	6.044
ENSG00000119865	CNRIP1	0.990	0.668	6.576	7.472	14.200	12.333	22.457	14.998	12.588	10.729	9.619
ENSG00000127922	SHFM1	0.979	0.569	19.780	12.247	9.945	12.453	14.449	14.811	17.365	12.933	15.751
ENSG00000120725	SIL1	0.961	0.957	16.180	14.800	11.026	9.544	12.039	8.911	9.163	6.127	6.981
ENSG00000168899	VAMP5	0.961	0.651	59.152	42.987	12.560	33.184	10.585	12.724	36.552	25.021	35.954
ENSG00000023191	RNH1	0.957	0.225	34.907	13.515	23.450	21.379	16.339	9.542	6.125	9.928	7.900
ENSG00000002822	MAD1L1	0.956	0.229	19.156	8.948	8.275	10.010	9.525	9.343	3.337	2.324	4.788
ENSG00000164292	RHOBTB3	0.953	0.805	6.949	6.581	6.699	6.464	8.424	6.643	20.842	14.337	18.072
ENSG00000032742	IFT88	0.949	0.934	4.263	3.079	5.170	2.260	4.367	2.502	8.179	9.040	7.595
ENSG00000064199	SPA17	0.949	0.903	1.934	2.088	1.664	2.254	2.207	2.853	4.977	7.571	7.282
ENSG00000166147	FBN1	0.948	0.888	11.171	6.547	8.354	5.558	6.996	10.383	88.437	16.180	72.608
ENSG00000095906	NUBP2	0.948	0.900	57.970	14.235	28.236	13.048	12.922	7.437	4.741	2.062	5.131
ENSG00000114279	FGF12	0.945	0.914	7.075	7.670	5.695	2.908	21.866	16.531	0.147	0.223	0.671
ENSG00000115762	PLEKHB2	0.943	0.897	11.110	17.381	19.784	8.413	58.318	33.807	3.766	6.126	6.050
ENSG00000087116	ADAMTS2	0.937	1.705	0.052	0.148	0.158	0.122	0.057	0.962	21.112	25.595	25.809
ENSG00000008196	TFAP2B	0.935	0.000	10.183	3.238	9.848	4.759	36.771	15.549	0.000	0.000	0.118
ENSG00000213123	TCTEX1D2	0.924	0.725	7.390	9.135	6.517	10.005	6.053	7.761	16.481	9.893	24.375
ENSG00000120860	CCDC53	0.909	0.655	7.989	7.723	8.426	5.480	6.432	6.706	18.174	11.653	19.330
ENSG00000143164	DCAF6	0.908	1.372	5.143	5.311	6.013	6.406	11.416	10.702	8.969	10.187	7.099
ENSG00000184254	ALDH1A3	0.896	0.583	9.097	8.185	10.196	10.839	12.770	32.460	32.240	56.170	16.116
ENSG00000143333	RGS16	0.893	0.620	50.997	41.913	49.352	41.662	67.874	21.097	6.049	5.566	10.299
ENSG00000115685	PPP1R7	0.893	1.033	7.322	19.570	7.119	8.177	8.614	6.994	12.532	12.197	9.778
ENSG00000178381	ZFAND2A	0.891	0.779	8.313	10.899	6.575	6.260	7.715	6.821	20.790	15.185	10.681
ENSG00000067082	KLF6	0.888	0.650	10.382	9.404	8.932	12.240	10.343	10.217	27.746	19.956	17.187
ENSG00000141741	MIEN1	0.885	0.428	23.930	18.447	8.867	9.948	8.708	7.477	14.652	18.877	9.866
ENSG00000134531	EMP1	0.872	0.011	10.338	13.003	10.509	12.333	31.807	38.085	18.409	15.576	20.110
ENSG00000101460	MAP1LC3A	0.871	0.781	46.719	16.862	9.614	17.946	11.402	9.207	7.752	14.680	14.845
ENSG00000260342	RP11-1035H13.3	0.870	0.932	15.408	12.759	10.824	23.742	13.053	14.447	22.659	20.300	58.016
ENSG00000110074	FOXRED1	0.860	0.204	11.885	8.719	9.180	6.987	8.586	5.308	3.811	2.480	4.096
ENSG00000197766	CFD	0.859	0.240	181.686	192.457	21.064	31.563	15.520	9.388	10.138	10.290	6.258
ENSG00000161513	FDXR	0.859	0.212	23.443	9.048	11.136	7.738	9.722	6.597	2.019	1.350	2.557
ENSG00000258102	MAP1LC3B2	0.858	0.625	10.651	10.785	9.217	14.672	18.578	18.984	45.228	53.759	44.349
ENSG00000130005	GAMT	0.855	0.968	181.082	72.983	56.396	51.404	36.367	15.534	16.081	13.834	9.851
ENSG00000105649	RAB3A	0.853	7.823	12.809	6.990	13.779	12.298	15.040	8.488	5.971	4.743	6.805
ENSG00000125726	CD70	0.849	0.399	252.949	176.527	97.812	110.831	70.520	35.944	2.479	0.435	0.211

ENSG00000143443	C1orf56	0.844	1.010	11.465	4.689	4.563	4.142	8.492	10.729	21.821	21.225	5.860
ENSG00000261884	CTC-479C5.12	0.843	0.607	12.505	13.380	2.858	3.701	2.313	2.107	3.490	3.453	3.896
ENSG00000108384	RAD51C	0.841	0.440	5.971	4.236	5.424	6.836	7.782	4.245	4.091	4.561	5.244
ENSG00000182492	BGN	0.837	0.510	0.269	0.119	0.152	0.195	0.117	0.152	13.295	16.587	25.059
ENSG00000165501	LRR1	0.835	0.952	13.501	12.853	11.846	6.287	11.011	9.121	7.467	8.858	7.555
ENSG00000139180	NDUFA9	0.835	0.543	40.654	78.682	49.748	40.500	38.360	26.924	11.206	14.107	13.720
ENSG00000121350	PYROXD1	0.834	1.003	2.311	3.178	3.433	3.085	2.037	4.391	10.909	9.441	7.260
ENSG00000105379	ETFB	0.831	0.557	138.221	114.642	42.096	76.724	26.703	23.352	13.607	10.174	15.662
ENSG00000100294	MCAT	0.829	0.572	67.175	26.332	16.930	12.244	18.446	8.385	6.640	5.883	4.829
ENSG00000130529	TRPM4	0.829	0.861	41.119	36.733	4.485	6.180	3.732	4.449	4.206	3.821	4.271
ENSG00000182645	CCDC172	0.824	0.430	2.317	4.317	4.430	2.418	5.218	3.417	15.705	21.155	21.018
ENSG00000174165	ZDHHC24	0.808	0.573	22.987	8.388	7.106	10.594	8.406	8.098	4.621	3.717	3.591
ENSG00000277972	CISD3	0.808	0.833	76.684	85.549	19.079	24.963	14.379	7.791	10.811	8.455	12.279
ENSG00000145623	OSMR	0.805	0.147	8.599	12.194	9.586	13.961	9.087	10.468	20.957	14.376	17.674
ENSG00000153071	DAB2	0.804	0.587	5.608	4.309	4.748	5.467	5.433	6.411	9.483	12.878	21.348
ENSG00000170961	HAS2	0.803	0.631	0.126	0.030	0.570	0.044	0.033	0.416	228.339	168.711	212.552
ENSG00000134352	IL6ST	0.801	0.650	8.264	6.266	6.281	5.253	10.167	7.407	10.558	7.120	15.788
ENSG00000117069	ST6GALNAC5	0.800	1.131	5.400	5.403	5.519	3.058	21.585	10.455	0.000	0.000	0.000
ENSG00000167112	TRUB2	0.791	0.380	28.761	15.351	25.529	18.280	26.253	16.596	6.657	5.140	4.843
ENSG00000116815	CD58	0.790	0.639	9.873	10.984	9.789	9.756	31.442	25.875	10.060	10.918	9.348
ENSG00000167645	YIF1B	0.784	0.845	17.529	23.931	62.695	17.050	31.843	37.589	4.923	3.969	9.029
ENSG00000165688	PMPCA	0.782	0.622	63.345	61.639	64.532	54.447	49.721	36.281	13.565	9.963	8.503
ENSG00000158062	UBXN11	0.778	0.819	11.022	13.463	3.748	4.979	3.247	5.060	4.739	6.068	5.229
ENSG00000176978	DPP7	0.773	0.475	33.394	7.081	18.082	18.555	16.624	20.979	5.011	6.098	5.277
ENSG00000108639	SYNGR2	0.770	0.563	14.566	9.153	15.858	10.680	9.438	7.981	5.027	3.920	4.450
ENSG00000205220	PSMB10	0.768	0.529	65.035	48.928	10.892	24.242	11.462	10.629	10.433	14.756	16.441
ENSG00000244405	ETV5	0.755	0.554	6.518	7.558	5.546	4.733	6.134	8.631	9.933	6.952	10.492
ENSG00000148481	FAM188A	0.755	0.707	6.993	7.485	6.453	8.050	11.630	9.593	8.114	11.492	15.252
ENSG00000093010	COMT	0.739	0.589	13.359	8.379	8.365	12.090	9.453	10.927	13.985	16.092	13.800
ENSG00000174917	C19orf70	0.736	0.757	245.969	177.228	30.867	60.917	19.464	15.621	43.207	28.696	49.548
ENSG00000175906	ARL4D	0.735	0.756	156.327	153.269	55.138	69.304	55.117	47.322	9.719	7.448	10.451
ENSG00000119048	UBE2B	0.735	0.690	12.361	13.602	10.552	12.811	12.082	13.070	18.146	16.635	32.032
ENSG00000102802	MEDAG	0.734	1.604	0.000	0.000	0.000	0.000	0.000	0.000	41.619	44.880	32.965
ENSG00000117308	GALE	0.734	0.414	15.180	9.515	9.645	12.565	9.377	7.548	5.306	3.660	6.781
ENSG00000164081	TEX264	0.724	0.784	12.771	9.553	6.088	8.826	7.776	5.219	6.722	7.203	5.862

ENSG00000174132	FAM174A	0.722	0.637	6.632	7.929	7.164	7.632	18.314	10.441	8.104	9.465	6.281
ENSG00000161677	JOSD2	0.719	0.640	58.797	38.516	14.306	22.441	10.295	15.383	16.273	23.484	11.835
ENSG00000187024	PTRH1	0.718	0.591	50.374	35.724	7.151	13.526	5.328	4.815	5.739	5.576	4.952
ENSG00000267544	AC007229.3	0.713	0.564	12.143	16.076	11.603	9.257	11.324	10.567	12.765	9.714	19.988
ENSG00000205352	PRR13	0.708	0.516	16.212	49.425	17.425	23.638	13.433	9.340	8.663	12.100	10.851
ENSG00000139209	SLC38A4	0.701	1.999	14.488	13.480	18.048	12.557	53.817	30.936	0.118	0.090	0.174
ENSG00000164932	CTHRC1	0.691	0.152	10.407	12.292	17.140	12.338	14.703	10.457	87.981	53.699	100.947
ENSG00000102309	PIN4	0.682	0.557	10.592	15.594	3.678	4.865	4.422	6.859	5.730	3.537	4.468
ENSG00000115657	ABCB6	0.675	0.600	6.759	3.119	11.349	8.714	19.613	12.123	2.723	3.100	3.985
ENSG00000015475	BID	0.672	0.350	22.389	53.648	8.322	16.716	9.886	10.938	5.452	6.650	4.947
ENSG00000167920	TMEM99	0.661	0.802	7.921	5.724	8.972	8.448	9.488	6.764	13.345	17.255	29.118
ENSG00000011422	PLAUR	0.658	0.853	13.515	24.526	13.588	13.521	12.110	15.799	68.247	37.485	78.789
ENSG00000090339	ICAM1	0.650	1.019	6.031	4.773	4.852	3.538	6.955	30.454	10.249	3.733	7.493
ENSG00000248098	BCKDHA	0.650	0.405	55.505	39.684	14.593	34.835	27.805	14.145	3.785	4.448	4.947
ENSG00000024862	CCDC28A	0.643	0.491	6.480	3.024	5.202	6.170	10.216	5.369	16.157	12.776	20.509
ENSG00000130513	GDF15	0.638	1.216	32.048	12.092	12.745	16.542	11.249	12.701	30.597	64.395	30.128
ENSG00000033011	ALG1	0.637	0.531	15.024	34.811	17.996	21.654	17.537	13.127	8.041	6.971	4.368
ENSG00000106991	ENG	0.632	0.850	10.241	13.982	14.673	15.500	12.928	8.349	31.573	15.595	38.084
ENSG00000243789	JMJD7	0.631	0.443	11.065	7.103	2.583	4.100	2.085	10.286	2.666	4.101	3.274
ENSG00000127125	PPCS	0.631	0.708	6.411	7.398	6.312	8.133	5.864	7.387	8.184	7.656	12.157
ENSG00000221988	PPT2	0.630	0.851	9.124	11.559	8.097	10.959	7.386	6.612	2.416	2.183	3.796
ENSG00000160688	FLAD1	0.629	0.146	58.992	50.054	20.701	31.234	12.040	14.198	5.676	2.062	2.081
ENSG00000134330	IAH1	0.625	1.005	9.286	10.443	6.724	6.544	8.602	11.935	21.173	14.560	16.817
ENSG00000173581	CCDC106	0.623	0.493	34.428	5.652	12.456	12.988	14.246	10.059	3.933	2.215	3.043
ENSG00000148180	GSN	0.602	0.740	14.059	15.266	40.941	81.022	40.057	145.641	15.958	7.886	10.036
ENSG00000167779	IGFBP6	0.601	0.996	8.482	8.974	5.919	10.481	7.650	9.868	17.983	31.146	16.535
ENSG00000160789	LMNA	0.596	0.858	15.920	8.453	24.158	23.704	17.995	27.412	8.753	8.708	10.011
ENSG00000112667	DNPH1	0.594	0.634	294.254	89.750	49.938	82.090	30.302	27.940	19.987	14.593	12.041
ENSG00000148331	ASB6	0.594	0.700	14.354	6.952	24.307	13.637	28.119	20.120	6.922	6.193	5.373
ENSG00000108679	LGALS3BP	0.593	0.715	40.391	13.071	24.152	74.052	25.348	64.155	7.409	9.235	8.644
ENSG00000026508	CD44	0.593	0.663	9.889	10.008	12.042	9.421	14.740	19.203	16.273	16.621	24.972
ENSG00000141994	DUS3L	0.585	0.576	9.043	2.300	10.867	7.757	10.794	6.092	5.932	4.781	6.546
ENSG00000147804	SLC39A4	0.582	0.774	33.274	33.142	6.605	12.062	4.874	3.219	6.283	4.403	3.760
ENSG00000167264	DUS2	0.571	0.711	8.621	7.604	9.372	10.074	10.482	8.424	5.333	3.578	4.164
ENSG00000106633	GCK	0.568	0.583	16.490	10.288	13.072	9.735	12.388	3.766	10.120	6.639	5.148

ENSG00000100290	BIK	0.566	1.068	13.209	10.004	73.851	8.990	33.217	6.048	4.865	1.925	3.762
ENSG00000136235	GPNMB	0.563	0.359	8.978	10.958	20.189	11.360	89.501	36.870	10.951	13.905	15.277
ENSG00000090674	MCOLN1	0.563	0.472	21.657	10.645	13.509	14.977	17.414	13.817	3.856	5.427	5.431
ENSG00000119632	IFI27L2	0.562	0.918	37.374	27.467	15.592	14.179	12.550	12.917	25.847	13.435	15.143
ENSG00000111364	DDX55	0.562	1.550	6.145	9.849	4.849	4.723	7.084	3.353	4.702	5.568	4.485
ENSG00000141905	NFIC	0.555	0.379	10.209	2.193	3.137	6.301	8.001	3.651	3.520	4.600	6.343
ENSG00000136936	XPA	0.535	0.401	6.865	6.204	8.411	2.951	12.984	10.644	14.560	14.379	15.098
ENSG00000175197	DDIT3	0.532	0.554	12.541	8.823	6.061	10.471	8.576	10.429	30.648	20.213	16.655
ENSG00000140092	FBLN5	0.531	0.520	3.266	2.682	3.214	2.854	5.062	4.161	4.812	3.352	12.438
ENSG00000136826	KLF4	0.530	0.501	7.101	5.540	11.137	19.087	13.471	6.491	2.147	2.165	3.976
ENSG00000129244	ATP1B2	0.530	0.580	26.008	6.782	10.502	6.976	23.799	19.732	5.331	3.502	4.425
ENSG00000103253	HAGHL	0.527	0.624	19.916	10.304	4.022	5.480	3.371	2.563	5.875	4.666	3.140
ENSG00000083123	BCKDHB	0.527	0.523	11.050	21.249	19.437	9.134	65.489	17.347	6.260	6.438	5.096
ENSG00000119574	ZBTB45	0.525	0.829	21.460	3.380	10.266	7.490	8.406	7.902	3.449	2.187	4.191
ENSG00000189129	PLAC9	0.524	0.767	8.027	8.865	0.978	7.327	10.841	9.968	28.934	15.001	27.393
ENSG00000100906	NFKBIA	0.523	0.404	25.914	10.804	13.656	12.230	15.929	14.562	10.533	15.143	4.857
ENSG00000189280	GJB5	0.521	0.200	4.826	16.694	3.267	6.384	2.532	10.907	0.000	0.000	0.000
ENSG00000139278	GLIPR1	0.520	0.569	0.270	0.447	0.256	0.115	0.623	1.771	53.593	21.654	38.113
ENSG00000016082	ISL1	0.519	0.053	6.335	8.904	10.845	4.360	20.004	20.198	0.000	0.261	0.454
ENSG00000143387	CTSK	0.519	0.290	7.050	6.093	12.113	10.350	10.206	5.130	48.819	60.196	36.716
ENSG00000113083	LOX	0.518	0.282	10.568	15.352	12.978	13.112	17.625	66.584	135.643	67.006	133.369
ENSG00000156384	SFR1	0.515	0.876	8.240	6.357	7.187	8.645	8.727	9.449	34.332	40.957	40.952
ENSG00000175701	LINC00116	0.515	0.602	13.377	6.539	5.569	6.057	6.303	5.865	12.566	8.571	10.671
ENSG00000196139	AKR1C3	0.513	0.181	3.066	3.149	2.013	4.808	4.105	4.795	13.660	8.210	6.866
ENSG00000188010	MORN2	0.513	0.752	9.803	12.431	5.328	9.753	7.970	8.198	23.551	14.117	21.545
ENSG00000166922	SCG5	0.513	0.951	13.468	12.570	12.853	12.201	10.416	8.942	85.634	32.413	45.102
ENSG00000177830	CHID1	0.509	0.426	16.134	12.918	9.947	11.115	8.611	9.392	2.882	3.953	2.268
ENSG00000127445	PIN1	0.508	0.595	104.067	83.512	18.322	38.041	8.302	5.518	4.411	3.593	6.524
ENSG00000175592	FOSL1	0.508	0.416	47.704	54.163	38.402	33.379	18.624	18.849	6.127	7.545	8.421
ENSG00000079150	FKBP7	0.507	0.430	7.880	6.411	6.351	6.248	8.438	8.893	9.379	6.324	11.377
ENSG00000105472	CLEC11A	0.505	0.820	208.777	97.714	37.298	53.268	31.270	42.892	16.581	18.601	18.088
ENSG00000002330	BAD	0.504	0.837	28.887	30.185	17.051	17.731	10.794	16.769	15.773	7.970	24.875
ENSG00000100413	POLR3H	0.501	0.268	14.945	28.028	13.230	11.091	12.287	10.243	8.530	6.387	7.365
ENSG00000099250	NRP1	0.498	0.233	8.314	7.937	8.019	10.962	9.935	21.509	27.764	17.534	22.919
ENSG00000100196	KDELR3	0.498	0.427	6.473	10.907	8.991	13.811	12.528	12.374	83.940	53.310	121.612

ENSG00000121680	PEX16	0.497	0.392	40.720	26.286	11.589	20.812	11.687	10.117	6.265	5.468	2.040
ENSG00000077463	SIRT6	0.494	0.602	12.045	3.855	6.988	6.883	4.746	4.419	3.396	2.869	4.292
ENSG00000256269	HMBS	0.492	0.306	27.892	27.639	15.974	20.223	11.358	7.966	6.898	3.715	4.620
ENSG00000121851	POLR3GL	0.491	0.418	8.553	19.687	6.946	6.630	9.123	10.138	12.666	8.540	14.798
ENSG00000104356	POP1	0.491	0.386	5.517	6.061	9.121	3.891	24.824	13.783	5.855	4.407	4.183
ENSG00000182809	CRIP2	0.481	0.470	24.823	17.172	20.649	17.929	11.889	11.597	6.648	3.911	5.575
ENSG00000167272	POP5	0.479	0.502	21.406	56.728	10.569	12.833	7.944	6.812	8.940	6.278	6.014
ENSG00000255823	MTRNR2L8	0.478	0.182	90.259	10.550	54.829	112.367	15.744	36.567	44.903	62.967	31.043
ENSG00000176171	BNIP3	0.478	1.212	15.698	16.878	15.347	15.115	31.319	15.621	84.448	76.244	133.819
ENSG00000119138	KLF9	0.473	0.623	6.496	4.835	6.044	3.971	10.778	6.401	14.228	11.085	9.548
ENSG00000104267	CA2	0.473	0.230	5.243	15.662	16.663	53.907	47.191	27.007	3.333	1.583	5.079
ENSG00000136153	LMO7	0.472	0.416	14.098	13.738	6.305	5.038	43.238	37.622	10.353	5.606	6.754
ENSG00000126709	IFI6	0.469	0.550	17.675	17.871	3.959	4.708	4.653	8.257	6.030	6.146	0.000
ENSG00000119185	ITGB1BP1	0.468	0.308	5.049	8.076	5.297	3.393	5.681	6.273	11.562	8.346	8.545
ENSG00000172201	ID4	0.466	0.351	5.033	3.157	4.374	2.456	4.021	4.241	2.711	9.611	3.389
ENSG00000149260	CAPN5	0.464	0.632	7.510	2.049	7.992	11.853	9.141	25.261	1.001	3.571	3.377
ENSG00000101463	SYNDIG1	0.455	0.877	14.909	8.717	14.489	13.641	32.846	8.137	8.996	10.036	6.049
ENSG00000144591	GMPPA	0.451	0.397	62.917	56.111	13.077	29.668	9.664	9.119	10.786	8.945	12.836
ENSG00000231925	TAPBP	0.451	1.622	11.914	6.586	11.602	13.903	12.268	18.507	12.774	10.522	10.775
ENSG00000145945	FAM50B	0.448	1.091	8.286	9.423	10.915	9.143	8.486	6.136	7.694	24.124	10.680
ENSG00000066248	NGEF	0.439	0.416	13.128	4.748	8.853	5.293	24.300	16.547	0.448	0.455	0.289
ENSG00000106348	IMPDH1	0.433	0.927	15.950	4.782	32.080	24.578	30.877	37.450	6.030	6.859	7.190
ENSG00000185088	RPS27L	0.432	0.581	13.282	32.952	8.962	15.272	9.577	8.990	36.881	22.192	50.835
ENSG00000109220	CHIC2	0.429	0.661	10.228	4.574	12.844	4.125	8.014	8.238	39.585	52.456	63.760
ENSG00000143774	GUK1	0.429	0.230	49.081	29.966	10.442	13.807	6.355	5.496	8.350	8.350	8.711
ENSG00000155366	RHOC	0.428	0.367	38.483	23.746	10.950	19.170	12.092	10.109	28.125	26.186	23.564
ENSG00000160050	CCDC28B	0.426	0.355	80.183	56.224	14.323	22.697	11.512	9.185	4.915	5.353	3.223
ENSG00000198925	ATG9A	0.425	0.447	5.538	2.386	8.392	5.184	12.337	12.975	3.118	4.249	6.258
ENSG00000196776	CD47	0.422	0.378	10.923	10.810	9.840	9.509	28.989	26.382	13.925	13.362	29.015
ENSG00000130066	SAT1	0.419	0.861	12.391	14.306	10.384	12.360	14.445	17.894	220.449	155.127	248.600
ENSG00000115665	SLC5A7	0.419	0.043	8.250	5.800	5.667	7.875	29.029	46.190	0.000	0.000	0.078
ENSG00000118855	MFSD1	0.419	0.387	5.459	8.395	6.184	1.456	8.947	10.141	8.423	8.607	10.640
ENSG00000150456	N6AMT2	0.417	0.408	10.318	23.935	4.580	8.754	7.325	4.354	14.713	8.352	6.398
ENSG00000257950	P2RX5-TAX1BP3	0.415	0.643	10.835	9.862	7.991	5.481	8.261	10.977	3.932	4.429	3.231
ENSG00000166825	ANPEP	0.413	0.531	23.255	22.115	26.835	20.541	20.993	111.499	51.940	56.101	67.224

ENSG00000122971	ACADS	0.413	0.516	17.944	4.326	7.673	5.709	8.441	4.370	2.510	1.310	2.876
ENSG00000167965	MLST8	0.413	0.412	12.311	5.568	7.445	8.299	4.973	4.592	2.345	4.092	2.648
ENSG00000104863	LIN7B	0.412	0.480	7.912	4.602	5.129	4.988	4.850	6.673	5.975	12.696	10.206
ENSG00000136271	DDX56	0.409	0.378	22.293	23.271	40.527	29.429	36.046	25.151	12.260	7.899	6.870
ENSG00000125454	SLC25A19	0.407	0.502	16.107	24.202	8.343	6.012	7.820	2.557	3.541	4.378	4.076
ENSG00000113369	ARRDC3	0.406	0.583	7.818	8.681	6.000	9.246	15.027	25.570	16.546	24.643	10.303
ENSG00000162604	TM2D1	0.402	0.301	4.980	3.027	4.384	5.895	3.739	4.402	8.516	5.398	4.453
ENSG00000057252	SOAT1	0.401	0.588	7.707	4.196	6.199	4.236	6.096	5.922	5.374	9.270	7.880
ENSG00000081985	IL12RB2	0.396	0.720	9.956	2.922	2.553	7.522	9.677	5.792	2.635	2.130	5.010
ENSG00000278763	FAM27B	0.390	0.319	20.469	26.989	13.325	9.367	12.255	5.182	8.231	7.566	6.823
ENSG00000164172	MOCS2	0.387	0.956	9.200	8.647	12.598	9.540	7.377	8.263	10.513	9.883	6.608
ENSG00000047634	SCML1	0.385	0.151	7.872	6.400	6.360	8.191	8.952	6.486	8.625	20.545	27.773
ENSG00000047634	SCML1	0.385	0.151	2.872	2.400	3.360	2.191	3.952	3.486	8.625	20.545	27.773
ENSG00000127666	TICAM1	0.384	0.274	7.584	4.051	7.449	6.021	8.929	10.305	3.064	4.658	5.512
ENSG00000130193	THEM6	0.384	1.846	23.324	5.255	24.320	27.509	24.604	18.978	0.129	0.819	0.000
ENSG00000110031	LPXN	0.383	0.303	4.244	3.384	8.495	8.416	9.668	5.662	31.226	48.877	34.514
ENSG00000133466	C1QTNF6	0.380	0.400	13.478	2.221	4.513	8.970	1.987	10.045	0.149	0.202	0.037
ENSG00000132004	FBXW9	0.380	0.617	10.357	39.854	38.340	40.657	21.567	19.591	6.681	8.799	11.172
ENSG00000152778	IFIT5	0.376	0.681	12.063	12.428	13.969	9.622	18.968	8.339	11.680	11.754	13.573
ENSG00000108479	GALK1	0.369	0.901	48.692	22.108	12.620	22.593	8.150	8.250	4.890	5.448	3.335
ENSG00000110090	CPT1A	0.368	0.397	12.300	6.517	6.785	4.985	30.790	17.725	5.616	6.745	7.009
ENSG00000108691	CCL2	0.362	0.378	9.115	8.015	7.864	12.631	10.078	14.187	20.700	11.338	10.055
ENSG00000099282	TSPAN15	0.362	0.643	3.965	2.725	8.673	2.906	10.672	2.641	2.632	3.521	0.000
ENSG00000116711	PLA2G4A	0.358	0.802	10.516	8.694	12.189	8.006	40.283	17.426	11.814	6.638	7.960
ENSG00000163359	COL6A3	0.357	0.751	0.000	0.005	0.024	0.032	0.005	0.045	130.652	102.419	119.917
ENSG00000106819	ASPN	0.353	0.000	6.001	9.158	10.860	9.641	21.248	13.847	2.278	3.203	3.275
ENSG00000258153	HSPE1P4	0.348	0.441	136.953	137.956	79.330	123.222	30.963	39.421	20.633	20.288	14.155
ENSG00000132141	CCT6B	0.347	0.371	2.521	2.555	2.892	2.810	3.609	3.393	2.545	5.781	6.618
ENSG00000147381	MAGEA4	0.346	0.783	7.568	8.120	5.456	5.560	6.880	7.559	26.527	31.764	17.107
ENSG00000145335	SNCA	0.345	0.340	4.781	11.777	6.559	7.627	7.293	4.277	3.954	4.955	5.191
ENSG00000004776	HSPB6	0.340	0.556	13.188	7.072	10.863	5.544	8.562	5.383	113.499	55.999	372.473
ENSG00000111907	TPD52L1	0.339	0.435	14.604	29.762	44.994	30.810	38.959	31.582	0.016	0.085	0.106
ENSG00000072682	P4HA2	0.327	0.532	8.169	7.368	9.515	5.980	10.218	8.669	14.111	15.223	19.754
ENSG00000092345	DAZL	0.327	0.654	12.126	10.158	20.123	8.567	15.357	11.127	75.258	120.604	86.650
ENSG00000013588	GPRC5A	0.320	0.051	66.125	19.231	21.738	10.017	73.156	6.859	0.679	0.649	0.107

ENSG00000168542	COL3A1	0.317	0.011	25.906	19.668	65.717	38.479	24.772	431.333	45.312	17.455	54.589
ENSG00000149541	B3GAT3	0.317	0.656	32.873	15.659	15.343	25.090	19.243	15.599	6.659	4.617	5.066
ENSG00000188375	H3F3C	0.314	0.353	17.530	68.575	29.649	24.289	8.763	9.514	6.012	9.308	6.187
ENSG00000170627	GTSF1	0.312	0.177	0.000	0.000	0.018	0.000	0.014	0.000	19.576	28.229	34.327
ENSG00000162302	RPS6KA4	0.307	0.308	4.586	3.543	12.129	5.087	9.400	19.853	5.363	6.586	4.310
ENSG00000083807	SLC27A5	0.306	0.348	12.075	22.580	3.484	4.504	10.984	2.125	4.656	4.300	3.394
ENSG00000074582	BCS1L	0.297	0.876	14.092	16.074	6.087	7.036	5.868	5.503	3.925	3.001	1.291
ENSG00000167754	KLK5	0.292	0.186	5.459	3.516	3.675	2.649	13.251	3.366	2.763	3.801	2.743
ENSG00000110328	GALNT18	0.291	0.213	4.872	4.996	8.332	9.424	11.705	19.095	2.122	5.160	6.722
ENSG00000197061	HIST1H4C	0.286	0.390	899.283	301.657	145.919	183.689	27.759	3.947	192.435	571.970	250.559
ENSG00000167799	NUDT8	0.286	0.395	36.737	17.947	8.455	12.969	5.524	4.766	4.139	6.487	3.338
ENSG00000112769	LAMA4	0.283	0.324	13.217	14.115	8.574	6.891	12.081	24.456	7.387	9.590	15.416
ENSG00000122641	INHBA	0.280	0.141	0.000	0.000	0.131	0.018	0.068	0.030	63.707	29.607	124.521
ENSG00000019549	SNAI2	0.265	0.108	11.683	10.064	15.137	12.436	8.455	33.313	49.986	140.395	58.795
ENSG00000204946	ZNF783	0.265	0.460	2.271	6.996	3.000	3.014	6.920	8.565	2.226	3.375	3.333
ENSG00000172005	MAL	0.263	0.029	11.434	3.013	2.696	3.423	8.504	2.153	0.000	0.141	0.000
ENSG00000116729	WLS	0.263	0.389	8.302	12.004	8.208	9.833	10.213	14.745	14.238	11.733	31.304
ENSG00000074416	MGLL	0.262	0.103	13.496	10.346	3.171	13.927	2.033	8.261	5.365	3.932	4.107
ENSG00000150347	ARID5B	0.256	0.092	6.119	8.882	13.262	5.311	14.050	18.023	6.657	5.346	7.289
ENSG00000006530	AGK	0.256	0.632	3.022	4.659	15.907	8.683	22.391	30.203	5.135	4.033	3.959
ENSG00000143369	ECM1	0.251	0.085	6.431	6.609	10.639	8.751	8.414	11.707	24.025	23.144	14.184
ENSG00000129009	ISLR	0.248	0.599	0.000	0.318	0.000	0.311	0.000	0.067	12.925	21.168	6.452
ENSG00000213699	SLC35F6	0.246	0.476	18.594	18.377	12.578	16.400	25.049	40.233	6.446	5.474	4.611
ENSG00000121542	SEC22A	0.242	0.814	5.049	6.184	6.964	7.915	5.955	6.988	7.920	8.300	9.902
ENSG00000163565	IFI16	0.241	0.283	11.221	10.858	12.560	12.254	13.146	41.617	25.456	15.688	12.288
ENSG00000001461	NIPAL3	0.241	0.958	8.137	11.690	2.549	2.906	4.704	10.047	2.657	1.787	3.066
ENSG00000119681	LTBP2	0.233	0.255	5.017	4.069	2.105	3.051	3.016	5.078	16.073	8.567	13.515
ENSG00000086506	HBQ1	0.232	0.380	41.921	39.704	15.691	20.131	11.008	30.547	12.950	8.356	10.423
ENSG00000188910	GJB3	0.228	0.220	10.101	10.804	9.991	8.644	14.751	9.044	0.000	0.000	0.000
ENSG00000161544	CYGB	0.226	0.431	0.470	0.085	0.201	0.274	0.031	0.348	34.642	27.384	13.074
ENSG00000109099	PMP22	0.221	0.255	8.057	7.821	6.005	5.849	6.037	28.267	12.306	6.573	7.691
ENSG00000101940	WDR13	0.219	0.216	19.177	15.584	9.542	13.429	7.948	7.175	3.467	4.280	5.723
ENSG00000147889	CDKN2A	0.218	0.116	11.205	6.702	7.491	14.456	4.911	5.855	5.513	4.158	6.313
ENSG00000176422	SPRYD4	0.217	1.037	8.670	10.860	5.801	5.790	6.924	5.737	3.326	1.697	4.057
ENSG00000164733	CTSB	0.216	0.992	12.468	10.729	9.924	13.233	10.053	22.284	73.349	84.791	101.515

ENSG00000107796	ACTA2	0.215	0.519	4.196	4.309	8.146	2.753	4.586	5.965	12.223	14.816	15.635
ENSG00000013297	CLDN11	0.212	0.233	19.763	3.046	10.355	7.024	12.894	6.144	1.392	1.789	2.573
ENSG00000104524	PYCRL	0.210	0.457	18.862	3.977	18.723	18.245	13.380	12.459	4.036	4.963	3.854
ENSG00000128422	KRT17	0.210	0.184	86.234	95.335	11.719	33.031	6.360	23.754	4.237	2.836	5.917
ENSG00000166946	CCNDBP1	0.205	0.304	6.282	5.487	4.134	6.010	6.160	5.652	4.349	7.526	8.808
ENSG00000100116	GCAT	0.205	0.199	22.570	25.766	41.160	50.831	28.091	17.644	4.122	8.408	2.731
ENSG00000136867	SLC31A2	0.203	0.115	8.246	6.329	5.566	3.175	4.986	4.945	20.925	19.309	5.736
ENSG00000146376	ARHGAP18	0.203	0.532	10.353	8.444	8.458	10.934	20.216	28.958	18.753	15.273	15.608
ENSG00000117525	F3	0.202	0.714	12.264	20.796	20.182	24.393	14.235	23.548	213.370	65.903	176.839
ENSG00000095303	PTGS1	0.200	0.191	12.216	10.559	9.923	10.255	5.523	8.808	26.128	36.315	46.767
ENSG00000148444	COMMD3	0.199	0.205	5.078	10.448	4.963	4.679	6.359	5.234	10.166	6.971	6.813
ENSG00000128335	APOL2	0.195	0.737	11.779	1.393	16.294	25.342	20.877	59.208	3.066	6.263	5.322
ENSG00000204652	RPS26P8	0.193	0.682	46.981	18.674	22.132	17.374	6.703	5.815	3.754	4.958	3.696
ENSG00000115363	EVA1A	0.193	0.168	10.270	8.208	10.994	6.298	15.861	6.110	5.572	6.421	20.765
ENSG00000092607	TBX15	0.185	0.038	8.096	10.814	5.548	4.335	22.131	15.918	0.000	0.000	0.000
ENSG00000151892	GFRA1	0.182	0.224	4.568	3.120	3.089	2.957	4.090	4.970	2.823	3.185	2.282
ENSG00000167646	DNAAF3	0.181	0.193	6.807	21.075	5.946	6.562	1.923	3.326	4.158	6.645	5.059
ENSG00000140465	CYP1A1	0.173	0.068	1.935	1.933	12.179	1.326	20.400	24.162	3.324	4.727	5.163
ENSG00000114251	WNT5A	0.165	0.154	0.000	0.000	0.012	0.000	0.022	0.020	16.264	17.324	11.171
ENSG00000007038	PRSS21	0.162	0.299	1.419	3.159	2.422	3.317	3.149	2.158	4.488	8.470	15.823
ENSG00000198692	EIF1AY	0.143	0.000	14.730	18.177	11.285	10.219	12.409	14.554	28.292	20.349	28.632
ENSG00000204228	HSD17B8	0.143	0.279	41.256	29.839	14.983	10.060	11.949	4.134	6.369	4.640	7.170
ENSG00000101336	HCK	0.141	0.196	3.035	3.035	2.841	5.621	3.949	6.060	2.223	3.510	2.041
ENSG00000263464	PPIAL4C	0.138	0.057	10.268	9.333	9.295	8.682	8.345	6.701	8.400	7.782	6.863
ENSG00000184349	EFNA5	0.134	0.462	43.394	26.839	18.254	11.025	65.989	98.387	4.706	10.949	10.296
ENSG00000117151	CTBS	0.133	0.220	6.139	4.171	4.340	4.374	5.109	5.716	15.068	10.416	8.029
ENSG00000188015	S100A3	0.129	0.079	41.096	49.155	4.327	41.928	1.756	47.382	22.270	16.689	7.704
ENSG00000162595	DIRAS3	0.127	0.019	1.120	4.441	5.333	6.689	2.876	23.681	0.000	0.000	0.000
ENSG00000100234	TIMP3	0.120	0.326	4.142	6.163	5.577	2.121	3.238	3.346	43.513	33.620	24.573
ENSG00000185551	NR2F2	0.116	0.012	7.844	8.252	10.836	5.748	11.998	7.364	29.511	15.172	46.444
ENSG00000166920	C15orf48	0.108	0.000	38.531	27.203	17.729	12.470	87.367	11.148	10.254	25.265	24.569
ENSG00000164171	ITGA2	0.107	0.207	6.522	5.842	10.147	7.522	8.038	10.312	38.253	11.073	45.974
ENSG00000127920	GNG11	0.107	0.188	10.892	12.332	10.739	13.593	14.424	17.246	239.362	156.990	186.947
ENSG00000080007	DDX43	0.105	0.236	0.000	0.000	0.000	0.000	0.000	0.000	15.519	24.569	22.972
ENSG00000112343	TRIM38	0.104	0.103	3.729	2.841	3.038	3.532	13.134	5.153	4.237	2.976	3.680

ENSG00000173559	NABP1	0.103	0.189	1.816	2.373	1.598	2.385	1.677	3.466	9.910	6.622	4.174
ENSG00000169059	VCX3A	0.092	0.076	6.317	5.319	5.139	7.521	8.076	4.318	35.974	60.876	41.513
ENSG00000196460	RFX8	0.092	0.140	0.000	0.000	0.000	0.000	0.000	0.000	13.218	14.029	12.764
ENSG00000123584	MAGEA9	0.092	0.056	4.417	5.417	3.418	4.832	3.038	3.522	18.335	33.187	19.094
ENSG00000162039	MEIOB	0.088	0.103	6.491	5.225	8.988	6.912	7.982	8.161	8.013	16.968	20.986
ENSG00000162039	MEIOB	0.088	1.034	3.491	2.225	3.988	2.912	2.982	4.161	8.013	16.968	20.986
ENSG00000198765	SYCP1	0.085	0.075	4.149	6.317	6.318	5.418	7.076	6.029	10.153	12.101	18.417
ENSG00000186103	ARGFX	0.084	0.068	18.789	10.107	13.386	4.313	18.881	3.709	2.313	0.000	0.000
ENSG00000186103	ARGFX	0.084	0.068	18.789	110.107	13.386	4.313	18.881	3.709	0.000	0.000	0.000
ENSG00000011465	DCN	0.080	0.004	6.467	8.846	7.759	15.532	9.932	8.912	93.798	43.439	101.666
ENSG00000007350	TKTL1	0.071	0.065	0.000	0.000	0.000	0.000	0.000	0.013	36.817	103.924	74.391
ENSG00000176046	NUPR1	0.070	0.063	8.550	11.825	7.941	5.720	8.994	5.648	15.365	34.077	12.392
ENSG00000204291	COL15A1	0.067	0.047	0.014	0.000	0.000	0.023	0.000	0.023	25.256	18.866	59.910
ENSG00000102967	DHODH	0.067	0.685	17.451	5.213	9.259	12.413	8.094	5.429	3.978	2.091	2.816
ENSG00000124575	HIST1H1D	0.062	0.051	15.384	10.696	2.127	2.933	3.957	10.829	12.125	7.261	15.638
ENSG00000167874	TMEM88	0.057	0.073	30.245	230.093	52.605	22.164	24.917	10.136	6.329	10.963	7.498
ENSG00000166796	LDHC	0.055	0.045	3.418	4.158	3.317	4.642	2.023	2.143	6.402	29.953	17.932
ENSG00000168298	HIST1H1E	0.055	0.135	23.418	10.304	10.644	8.974	11.001	8.081	8.616	13.491	10.140
ENSG00000143194	MAEL	0.049	0.088	6.043	5.373	8.259	6.364	4.259	9.422	30.938	45.432	39.486
ENSG00000267978	MAGEA9B	0.048	0.059	3.316	3.318	4.419	7.417	5.020	6.318	14.959	19.787	16.302
ENSG00000128040	SPINK2	0.047	0.052	0.000	0.000	0.000	0.000	0.000	0.000	21.379	11.567	26.030
ENSG00000122862	SRGN	0.041	0.017	0.108	0.062	0.264	0.185	0.614	0.458	428.456	150.831	385.000
ENSG00000165949	IFI27	0.041	0.062	8.848	13.750	7.089	11.537	6.980	6.824	6.539	6.603	5.439
ENSG00000130592	LSP1	0.040	0.028	19.009	12.654	7.610	26.693	6.635	6.035	4.845	5.516	3.133
ENSG00000182583	VCX	0.038	0.046	7.138	5.417	4.316	4.319	6.093	6.417	22.430	45.769	23.694
ENSG00000137767	SQRDL	0.035	0.070	8.336	8.934	10.711	5.928	6.102	6.198	12.705	10.708	15.553
ENSG00000148671	ADIRF	0.031	0.025	6.246	8.744	5.198	5.346	6.358	6.137	37.529	28.187	16.592
ENSG00000131015	ULBP2	0.031	0.201	8.645	13.989	10.775	9.861	8.951	11.728	6.823	26.949	39.680
ENSG00000185686	PRAME	0.030	0.004	4.315	5.297	6.652	6.432	4.496	5.829	15.010	25.327	11.811
ENSG00000187601	MAGEH1	0.028	0.979	4.522	8.085	8.979	6.622	10.215	5.042	24.282	15.513	55.996
ENSG00000109906	PLZF	0.028	0.030	2.133	4.773	2.147	3.310	3.222	3.177	3.972	3.405	3.215
ENSG00000152430	BOULE	0.023	0.005	6.302	3.115	4.567	3.589	5.678	5.146	8.061	7.496	20.000
ENSG00000214107	MAGEB1	0.020	0.100	4.418	3.218	3.169	4.217	4.317	5.316	16.212	36.686	11.036
ENSG00000131126	TEX101	0.020	0.033	6.369	5.248	4.346	6.942	5.269	8.030	73.682	69.726	176.140
ENSG00000160886	LY6K	0.016	0.062	0.169	0.211	0.103	0.096	0.133	0.103	22.149	12.015	17.639

ENSG00000205642	VCX3B	0.015	0.000	5.078	4.045	0.000	6.137	4.148	7.126	10.136	26.010	17.456
ENSG00000078098	FAP	0.013	0.000	0.000	0.115	0.100	0.057	0.070	0.082	19.601	15.753	28.482
ENSG00000116132	PRRX1	0.011	0.025	0.060	0.069	0.065	0.043	0.038	0.084	39.476	40.615	58.391
ENSG00000139351	SYCP3	0.008	0.012	5.335	6.113	5.032	5.134	4.334	4.244	21.087	57.474	64.454
ENSG00000171402	XAGE3	0.000	0.000	5.432	4.268	3.158	3.632	2.415	4.522	12.192	16.300	9.834
ENSG00000196570	PFN3	0.000	0.064	7.198	4.480	3.341	3.176	3.945	3.248	4.135	13.530	5.027
ENSG00000238269	PAGE2B	0.000	0.000	8.418	5.513	10.215	5.417	9.188	4.418	90.232	53.610	42.079
ENSG00000180389	ATP5EP2	0.000	0.088	20.384	31.162	17.476	23.436	17.663	10.034	57.487	27.288	30.826
ENSG00000158639	PAGE5	0.000	0.000	7.318	8.148	5.218	4.159	6.316	9.417	56.349	64.957	31.833
ENSG00000108342	CSF3	0.000	0.000	4.542	5.021	2.458	5.047	3.023	4.153	18.124	7.814	8.525
ENSG00000046774	MAGEC2	0.000	0.000	3.317	2.832	3.317	3.317	4.316	5.417	11.006	37.691	16.423
ENSG00000189023	MAGEB16	0.000	0.033	3.316	5.416	4.316	6.417	3.417	4.422	10.622	14.065	22.072
ENSG00000143452	HORMAD1	0.000	0.060	1.806	1.687	1.560	1.562	2.120	1.060	10.166	37.741	55.429
ENSG00000152670	DDX4	0.000	0.006	9.257	8.128	8.126	4.789	6.524	10.126	9.698	23.008	20.681
ENSG00000164113	ADAD1	0.000	0.000	2.345	2.120	3.126	3.157	3.891	4.345	7.405	14.610	3.335
ENSG00000125207	PIWIL1	0.000	0.000	1.401	1.678	1.568	1.723	2.126	2.512	5.694	2.076	4.259
ENSG00000068985	PAGE1	0.000	0.000	6.422	8.417	4.147	5.317	5.416	9.417	97.210	113.097	109.631
ENSG00000236362	GAGE12F	0.000	0.000	0.000	0.000	0.000	0.000	0.000	0.000	39.918	56.642	35.389
ENSG00000258484	SPESP1	0.000	0.000	6.529	5.419	5.349	9.159	7.427	8.418	26.467	31.404	29.446
ENSG00000241794	SPRR2A	0.000	0.000	11.991	23.796	1.998	6.560	5.613	10.465	1.235	2.346	1.985
ENSG00000176988	FMR1NB	0.000	0.000	6.247	5.359	8.136	8.148	7.422	7.316	109.404	178.832	147.493
ENSG00000181617	FDCSP	0.000	0.000	40.055	49.901	5.944	6.574	5.576	0.433	4.157	5.129	2.921
ENSG00000256618	MTRNR2L1	0.000	0.000	40.005	5.633	25.652	55.285	7.289	18.756	3.141	4.107	3.092
ENSG00000187581	COX8C	0.000	0.000	0.000	0.235	0.000	0.175	0.000	0.000	139.374	125.149	224.030
ENSG00000123496	IL13RA2	0.000	0.000	0.057	0.000	0.000	0.049	0.000	0.129	74.508	297.567	487.733
ENSG00000274391	TPTE	0.000	0.000	0.000	0.000	0.000	0.000	0.043	0.000	26.069	35.415	50.367
ENSG00000166407	LMO1	0.000	0.023	20.387	48.889	10.523	7.028	12.026	3.809	0.000	0.280	0.000
ENSG00000130595	TNNT3	0.000	0.000	21.467	17.480	5.053	11.460	9.829	86.047	0.000	0.000	0.137
