# Supplementary material for: Derivation and propagation of spermatogonial stem cells from human pluripotent cells
Source: Stem Cell Res Ther. 2020 Sep 23;11:408. doi: 10.1186/s13287-020-01896-0 (PMC7509941; doi:10.1186/s13287-020-01896-0)
Supplement: Supplementary file 1 — Additional file 1: Table S1. Primers of real-time PCR for germ cell markers. Tables S2. List of 1042 transcripts and their normalized FPRM from RNA-seq in the group of hPSCs, SSCLCs and GPR125+ cells isolated from human testes. Related to Fig. 4. Tables S3. List of transcripts related to pluripotency, SSC markers, germ cells and their FPKM from RNA-seq in the group of hPSCs, SSCLCs and GPR125+ cells. Related to Fig. 4 and Figure S2. Table S4. SSCLCs restore recipient testicular spermatogenesis after transplantation at different time points by Johnsen’s Score. Related to Figure S3A. [file 13287_2020_1896_MOESM1_ESM.zip › Tables S3_ESM.pdf]

**gene_id**	gene name	hiPS_FPKM	hES2_FPKM	hiPS-SSCLCs-P1-1_FPKM	hiPS-SSCLCs-P1-2_FPKM	hiPS-SSCLCs-P7-1_FPKM	hiPS-SSCLCs-P7-2_FPKM	hES2-SSCLCs-P1-1_FPKM	hES2-SSCLCs-P1-2_FPKM	SSC1_FPKM	SSC2_FPKM	SSC3_FPKM
**Pluripotency-related genes**												
ENSG00000204531	POU5F1	229.914	197.223	10.740	6.944	14.278	10.306	12.073	6.594	0.000	0.000	0.000
ENSG00000111704	NANOG	14.358	14.786	0.000	0.000	0.013	0.000	0.051	0.145	0.000	0.000	0.000
ENSG00000131914	LIN28A	314.452	290.440	0.015	0.035	4.474	0.000	0.058	0.035	0.000	0.000	0.000
ENSG00000179059	ZFP42	44.082	32.323	0.000	0.000	0.750	0.000	0.028	0.000	0.210	0.406	0.621
ENSG00000243709	LEFTY1	21.811	29.336	0.051	0.000	0.041	0.044	0.064	0.029	0.336	0.308	0.000
ENSG00000171794	UTF1	16.855	17.914	0.000	0.000	0.000	0.161	0.000	0.000	1.136	1.306	1.256
ENSG00000088305	DNMT3B	622.774	648.715	0.499	0.297	0.946	0.110	0.559	0.135	0.297	1.273	1.233
ENSG00000128567	PODXL	157.426	162.520	0.654	0.563	0.880	0.445	1.010	0.852	0.206	0.281	0.000
ENSG00000145423	SFRP2	312.107	394.938	0.000	0.000	0.788	0.000	0.236	0.000	0.000	0.000	0.119
ENSG00000147596	PRDM14	42.921	38.778	0.000	0.000	0.000	0.000	0.000	0.000	0.000	0.000	0.000
ENSG00000138336	TET1	18.764	17.750	0.760	0.431	0.381	0.381	1.009	0.693	0.731	0.269	0.052
ENSG00000272398	CD24	133.169	119.321	0.013	0.060	1.886	0.155	0.073	0.401	1.017	1.032	0.000
ENSG00000206557	TRIM71	84.278	83.423	0.000	0.000	0.275	0.032	0.000	0.056	0.404	2.266	0.809
ENSG00000155760	FZD7	220.814	215.598	0.339	0.097	0.321	0.096	0.373	0.302	2.101	2.334	2.071
ENSG00000145423	SFRP2	312.107	394.938	0.000	0.000	2.788	0.000	0.236	0.000	0.000	0.000	0.119
ENSG00000134323	MYCN	75.580	61.690	0.000	0.000	0.204	0.000	0.000	0.000	0.000	0.256	0.000
ENSG00000179899	PHC1P1	172.989	206.133	0.107	0.204	0.492	0.122	0.964	0.541	1.145	0.163	1.424
ENSG00000241186	TDGF1	32.159	28.054	0.000	0.000	0.036	0.000	0.047	0.000	0.357	0.000	0.000
ENSG00000160973	FOXH1	36.589	30.705	0.109	0.000	0.296	0.000	0.000	0.409	0.000	0.000	0.000
ENSG00000130294	KIF1A	39.885	32.945	0.356	0.096	0.431	0.006	0.305	0.170	0.122	0.301	0.000
ENSG00000165821	SALL2	56.216	43.011	0.501	0.232	0.599	0.487	0.531	1.013	0.283	0.457	0.000
ENSG00000175928	LRRN1	88.171	70.619	0.066	0.000	0.479	0.168	0.000	0.615	0.000	0.201	0.000
ENSG00000187140	FOXD3	16.124	11.334	0.000	0.000	0.000	0.000	0.000	0.000	0.000	0.000	0.348
ENSG00000114739	ACVR2B	13.675	9.804	0.481	0.112	0.206	0.155	0.405	0.055	0.946	1.014	0.029
ENSG00000043355	ZIC2	24.087	9.256	0.040	0.092	0.016	0.068	0.013	0.000	0.483	1.225	2.001
ENSG00000156925	ZIC3	19.794	43.414	0.000	0.000	0.016	0.000	0.000	0.000	1.282	0.059	1.460
ENSG00000139800	ZIC5	10.007	7.919	0.094	0.054	0.038	0.040	0.104	0.013	0.047	0.180	0.470
ENSG00000130182	ZSCAN10	20.626	32.482	0.000	0.000	0.061	0.000	0.027	0.018	0.000	0.000	0.000
ENSG00000104112	SCG3	27.563	22.993	0.000	0.000	0.554	0.000	0.237	0.013	0.946	1.836	0.628
ENSG00000039068	CDH1	19.281	18.398	0.015	0.009	0.649	0.006	0.014	0.008	0.000	0.023	0.084
ENSG00000138685	FGF2	11.235	9.598	0.138	0.143	0.117	0.241	0.348	0.255	3.411	5.392	10.038
ENSG00000180182	MED14	18.824	26.512	0.385	0.486	0.258	0.543	1.935	3.187	1.317	2.536	2.554
ENSG00000067840	PDZD4	14.615	43.810	0.000	0.000	0.000	0.000	0.000	0.000	0.387	0.000	0.000
ENSG00000168280	KIF5C	10.715	14.569	0.000	0.000	0.000	0.000	0.020	0.000	0.499	1.237	0.000
ENSG00000119772	DNMT3A	9.763	8.814	0.549	0.234	0.591	0.381	1.210	1.146	0.886	1.505	1.632
ENSG00000104332	SFRP1	69.179	18.948	0.105	0.151	0.203	0.146	0.166	0.081	0.577	0.563	0.380
ENSG00000152284	TCF7L1	55.941	34.695	0.209	0.000	0.245	0.119	0.132	0.222	1.727	1.172	0.722

ENSG00000240563	L1TD1	429.733	370.175	1.303	4.286	3.726	3.278	1.932	3.667	0.683	1.603	0.000
ENSG00000265992	ESRG	389.761	563.594	0.035	0.000	0.423	1.007	0.153	0.254	0.000	0.000	0.000
ENSG00000133980	VRTN	79.653	55.251	0.000	0.000	0.133	0.000	0.034	0.000	0.000	0.000	0.000
ENSG00000147601	TERF1	68.043	58.617	1.410	1.398	1.012	0.644	2.806	1.546	2.030	3.108	3.316
ENSG00000130203	APOE	210.151	216.173	1.158	0.162	0.551	0.096	0.124	1.081	0.114	1.124	0.000
ENSG00000206557	TRIM71	84.278	83.423	0.000	0.000	0.275	0.032	0.000	0.056	0.404	2.266	0.809
ENSG00000165821	SALL2	56.216	43.011	0.501	0.232	0.599	0.487	0.531	1.013	0.283	0.457	0.000
ENSG00000008311	AASS	79.350	66.657	0.168	0.289	0.728	0.658	2.143	4.177	1.042	1.715	5.354
ENSG00000166831	RBPMS2	83.308	71.072	0.442	0.117	1.160	0.000	0.407	0.727	0.821	0.465	0.000
ENSG00000111752	PHC1	43.807	47.342	0.066	0.124	0.262	0.092	0.224	0.196	2.032	1.699	0.921
ENSG00000141736	ERBB2	29.077	18.163	0.540	0.427	0.469	0.297	0.680	0.845	0.262	0.285	0.028
ENSG00000106278	PTPRZ1	36.021	39.612	0.000	0.009	0.000	0.000	0.069	0.607	0.000	0.000	0.556
ENSG00000046604	DSG2	73.794	76.347	0.000	0.019	1.225	0.043	0.043	0.562	2.279	1.961	0.565
ENSG00000140575	IQGAP1	20.825	19.867	0.604	0.399	0.751	0.715	2.746	3.860	3.744	3.574	3.254
ENSG00000165349	SLC7A3	133.300	97.426	0.000	0.000	1.991	0.000	0.000	0.000	0.000	0.000	0.000
ENSG00000092421	SEMA6A	21.117	25.725	0.057	0.013	0.579	0.058	0.132	0.131	0.444	0.095	0.000
ENSG00000157106	SMG1	10.115	9.437	0.309	0.377	0.313	0.323	1.335	1.380	2.195	2.531	2.709
ENSG00000187678	SPRY4	36.975	44.150	1.964	0.737	1.113	0.973	2.404	3.785	3.872	4.785	4.055
ENSG00000075340	ADD2	16.928	15.529	0.117	0.187	0.199	0.095	0.286	1.023	0.023	0.357	0.000
ENSG00000119139	TJP2	28.913	26.174	1.556	1.594	0.869	0.461	1.743	0.428	1.862	1.148	0.981
ENSG00000050344	NFE2L3	39.095	39.071	0.105	0.100	0.255	0.388	0.748	2.064	1.967	2.834	0.039
ENSG00000144730	IL17RD	17.180	16.427	0.008	0.036	0.071	0.027	0.060	0.085	0.956	1.334	0.616
ENSG00000177425	PAWR	15.620	15.318	0.131	0.183	0.177	0.217	0.275	0.745	1.682	2.081	0.501
ENSG00000114739	ACVR2B	13.675	9.804	0.481	0.112	0.206	0.155	0.405	0.055	1.946	1.014	0.029
ENSG00000137693	YAP1	21.748	22.017	0.270	0.596	0.177	0.541	0.308	1.374	3.604	2.560	4.227
ENSG00000157110	RBPMS	14.479	10.861	0.021	0.000	0.098	0.024	0.036	0.080	3.338	1.697	3.344
ENSG00000127946	HIP1	15.787	14.357	0.346	0.316	0.236	0.215	0.292	0.602	1.043	1.047	1.677
ENSG00000104413	ESRP1	20.350	21.073	0.000	0.000	0.457	0.009	0.045	0.046	0.000	0.078	0.000
ENSG00000022556	NLRP2	20.036	9.770	0.000	0.000	1.064	0.009	0.025	0.000	1.054	0.339	0.000
ENSG00000146904	EPHA1	30.927	31.011	0.063	0.145	0.502	0.352	0.060	0.250	0.255	0.097	0.528
ENSG00000131899	LLGL1	24.774	24.949	0.753	0.059	0.176	0.272	0.112	0.115	0.051	0.137	0.028
ENSG00000188322	SBK1	42.215	35.980	0.742	0.050	0.301	0.149	0.316	0.049	0.263	0.301	0.000
ENSG00000130182	ZSCAN10	20.626	32.482	0.000	0.000	0.061	0.000	0.027	0.000	0.000	0.000	0.000
ENSG00000062038	CDH3	21.248	22.190	0.012	0.040	0.588	0.030	0.029	0.945	0.559	0.319	0.000
ENSG00000066735	KIF26A	26.970	29.776	0.152	0.052	0.074	0.000	0.105	0.026	0.397	0.000	0.236
ENSG00000156453	PCDH1	10.416	8.562	0.000	0.007	0.040	0.047	0.000	0.010	0.000	0.112	0.353
ENSG00000143375	CGN	20.680	15.484	0.000	0.093	0.999	0.035	0.655	0.160	0.000	0.620	0.000
ENSG00000128849	CGNL1	19.080	16.451	0.000	0.000	0.213	0.000	0.039	0.000	0.000	0.057	0.000
ENSG00000126464	PRR12	17.416	14.944	0.973	0.199	0.320	0.514	0.262	0.508	0.210	0.514	0.720
ENSG00000081913	PHLPP1	16.451	14.846	0.198	0.152	0.215	0.235	0.562	0.285	2.039	0.961	7.276
ENSG00000162551	ALPL	25.334	45.430	0.052	0.059	1.438	0.029	0.411	0.251	0.035	0.289	1.222

ENSG00000133818	RRAS2	17.819	18.261	0.135	0.463	0.298	0.157	0.370	0.557	2.730	2.489	3.941
ENSG00000185483	ROR1	13.350	7.565	0.046	0.021	0.090	0.047	0.006	0.088	0.074	0.000	0.143
ENSG00000128602	SMO	32.542	26.859	0.385	0.104	0.405	0.387	0.057	0.153	0.000	0.000	0.000
ENSG00000143842	SOX13	12.217	12.749	0.036	0.041	0.206	0.154	0.097	0.417	0.000	0.249	0.000
ENSG00000184697	CLDN6	41.129	34.397	0.092	0.455	1.538	0.652	0.211	0.052	0.000	11.307	3.597
ENSG00000275832	ARHGAP23	14.995	14.882	0.136	0.026	0.073	0.058	0.014	0.038	1.501	2.232	0.377
ENSG00000143494	VASH2	9.117	25.538	0.036	0.050	0.036	0.100	0.096	0.070	0.000	0.000	0.000
ENSG00000136383	ALPK3	11.576	11.663	0.020	0.079	0.080	0.000	0.043	0.028	0.650	0.075	0.000
ENSG00000104112	SCG3	27.563	22.993	0.000	0.000	0.554	0.000	0.237	0.013	0.946	1.836	0.628
ENSG00000147676	MAL2	32.010	42.734	0.000	0.000	0.297	0.072	0.265	0.000	1.751	6.877	5.930
ENSG00000099364	FBXL19	12.577	10.796	0.180	0.069	0.029	0.113	0.023	0.027	0.072	0.018	0.000
ENSG00000174721	FGFBP3	42.259	42.052	0.814	0.540	0.139	0.073	0.162	0.289	0.000	0.262	0.000
ENSG00000130768	SMPDL3B	24.617	13.562	0.125	0.029	0.732	0.299	0.158	0.141	0.000	0.575	0.000
ENSG00000136110	LECT1	43.466	71.374	0.177	0.712	0.288	2.005	0.251	1.421	0.000	2.035	0.000
ENSG00000125753	VASP	15.534	11.202	0.826	0.127	0.516	0.244	0.770	0.652	0.255	0.948	0.212
ENSG00000241697	TMEFF1	37.389	41.106	0.125	0.479	0.509	0.000	0.788	0.000	3.273	0.894	1.392
ENSG00000128045	RASL11B	22.101	19.840	0.000	0.029	0.349	0.065	0.143	0.171	0.915	4.641	2.640
ENSG00000167680	SEMA6B	16.515	16.024	0.201	0.051	0.072	0.000	0.000	0.000	0.359	0.000	0.000
ENSG00000132297	HHLA1	17.099	34.336	0.000	0.000	0.000	0.000	0.000	0.000	0.000	0.000	0.000
ENSG00000180340	FZD2	21.096	15.670	0.052	0.059	0.419	0.132	0.000	0.174	1.735	0.869	0.670
ENSG00000182871	COL18A1	33.973	36.159	0.191	0.164	0.194	0.122	0.475	0.483	1.958	0.497	1.103
ENSG00000101115	SALL4	29.161	25.163	0.982	0.439	1.401	1.053	2.714	1.440	0.000	0.898	0.000
ENSG00000160963	COL26A1	21.302	20.224	0.000	0.000	0.160	0.000	0.000	0.000	0.000	0.515	0.000
ENSG00000103449	SALL1	11.756	17.214	0.881	0.477	0.473	0.341	1.120	2.235	2.217	0.978	0.000
ENSG00000102755	FLT1	13.880	8.113	0.000	0.009	0.000	0.000	0.068	0.039	0.603	0.176	0.444
ENSG00000077782	FGFR1	10.002	10.558	0.038	0.050	0.083	0.102	0.062	0.095	0.603	0.156	1.960
ENSG00000163530	DPPA2	9.638	14.128	0.079	1.627	1.088	0.605	0.744	2.040	2.535	1.235	0.000
ENSG00000154310	TNIK	13.416	9.809	0.125	0.092	0.312	0.069	0.338	0.327	0.665	0.383	0.616
ENSG00000137642	SORL1	11.281	10.638	0.039	0.011	0.249	0.037	0.058	0.055	0.391	0.268	0.000
ENSG00000080345	RIF1	11.150	11.703	0.736	0.392	0.575	0.528	3.614	2.771	4.070	3.936	4.540
ENSG00000182866	LCK	10.320	10.373	0.000	0.014	0.000	0.000	0.000	0.000	0.000	0.674	0.000
ENSG00000105647	PIK3R2	10.235	9.510	0.112	0.064	0.011	0.060	0.009	0.016	1.383	0.043	1.248
ENSG00000138759	FRAS1	11.939	7.013	0.009	0.000	0.125	0.012	0.003	0.236	0.000	0.346	0.161
ENSG00000151276	MAGI1	7.836	5.930	0.120	0.162	0.058	0.084	0.062	0.202	0.942	1.167	2.107
ENSG00000165804	ZNF219	10.449	8.961	0.726	0.407	0.185	0.173	0.069	0.104	0.051	0.181	0.131
ENSG00000169783	LINGO1	10.095	8.009	0.032	0.049	0.070	0.018	0.034	0.018	0.000	0.000	0.000
ENSG00000092758	COL9A3	8.247	11.829	0.086	0.028	0.060	0.010	0.062	0.007	0.467	0.037	0.136
ENSG00000099954	CECR2	8.459	7.661	0.075	0.023	0.099	0.017	0.013	0.004	0.027	0.332	0.000
ENSG00000130396	MLLT4	9.666	8.161	0.142	0.081	0.112	0.071	0.126	0.166	0.494	0.638	1.333
ENSG00000142449	FBN3	9.443	8.897	0.000	0.000	0.401	0.000	0.011	0.000	0.000	0.140	0.000
ENSG00000237515	SHISA9	9.186	5.241	0.046	0.000	0.030	0.000	0.023	0.000	0.000	0.185	0.000

ENSG00000156574	NODAL	9.743	6.975	0.000	0.000	0.087	0.000	0.000	0.000	0.000	0.000	0.000
ENSG00000125966	MMP24	8.711	9.153	0.247	0.000	0.060	0.063	0.000	0.181	1.489	0.076	0.000
ENSG00000101680	LAMA1	6.820	7.152	0.051	0.065	0.123	0.014	0.050	0.291	0.611	0.577	0.350
ENSG00000067141	NEO1	8.275	6.446	0.147	0.064	0.171	0.054	0.040	0.343	0.973	1.438	2.900
ENSG00000046889	PREX2	7.547	6.949	0.000	0.010	0.063	0.000	0.005	0.161	0.000	0.013	0.000
ENSG00000007062	PROM1	8.829	4.990	0.008	0.018	0.190	0.047	0.069	0.202	0.110	0.107	0.000
ENSG00000116852	KIF21B	6.274	7.122	0.040	0.027	0.026	0.007	0.000	0.013	0.032	1.052	0.000
ENSG00000197119	SLC25A29	6.372	6.900	0.410	0.174	0.193	0.276	0.299	0.236	0.071	0.449	0.292
ENSG00000166833	NAV2	7.629	4.228	0.017	0.000	0.057	0.000	0.018	0.039	0.245	0.329	0.452
ENSG00000148143	ZNF462	6.012	5.048	0.000	0.000	0.202	0.009	0.000	0.000	0.510	0.158	0.218
ENSG00000163694	RBM47	6.501	5.454	0.011	0.046	0.494	0.005	0.504	0.163	0.514	0.452	0.063
ENSG00000001617	SEMA3F	6.868	6.109	0.000	0.000	0.009	0.019	0.000	0.038	0.000	0.000	0.000
**SSC-related genes**												
ENSG00000010278	CD9	1.673	1.140	10.798	12.355	13.624	16.229	13.051	13.142	40.399	21.746	16.876
ENSG00000154096	CD90	3.186	3.661	53.823	66.722	57.564	210.217	17.233	150.464	8.140	13.858	8.961
ENSG00000152670	DDX4	0.000	0.006	9.257	8.128	8.126	4.789	6.524	10.126	9.698	23.008	20.681
ENSG00000154277	UCHL1	1.742	1.918	39.650	65.513	87.411	62.796	100.225	68.478	110.415	129.179	74.544
ENSG00000150093	ITGB1	2.972	2.295	14.387	15.488	17.592	13.298	33.685	38.099	113.070	51.828	131.239
ENSG00000147381	MAGEA4	0.346	0.783	7.568	8.120	5.456	5.560	6.880	7.559	26.527	31.764	17.107
ENSG00000244405	ETV5	0.755	0.554	6.518	7.558	5.546	4.733	6.134	8.631	9.933	6.952	10.492
ENSG00000172201	ID4	0.466	0.351	5.033	3.157	4.374	2.456	4.021	4.241	2.711	9.611	3.389
ENSG00000150907	FOXO1	1.653	1.248	4.023	2.832	6.629	3.054	5.230	4.421	6.263	8.122	7.660
ENSG00000151892	GFRA1	0.182	0.224	4.568	3.120	3.089	2.957	4.090	4.970	2.823	3.185	2.282
ENSG00000109906	PLZF	0.028	0.030	2.133	4.773	2.147	3.310	3.222	3.177	3.972	3.405	3.215
ENSG00000125207	PIWIL1	0.000	0.000	1.401	1.678	1.568	1.723	2.126	2.512	5.694	2.076	4.259
ENSG00000092345	DAZL	0.327	0.654	12.126	10.158	20.123	8.567	15.357	11.127	75.258	120.604	86.650
ENSG00000152430	BOULE	0.023	0.005	6.302	3.115	4.567	3.589	5.678	5.146	8.061	7.496	20.000
ENSG00000091409	ITGA6	23.953	20.333	2.121	2.160	2.561	3.367	2.140	5.571	9.132	3.715	4.809
ENSG00000143452	HORMAD1	0.000	0.060	1.806	1.687	1.560	1.562	2.120	1.060	10.166	37.741	55.429
ENSG00000164113	ADAD1	0.000	0.000	2.345	2.120	3.126	3.157	3.891	4.345	7.405	14.610	3.335
ENSG00000187569	STELLA	1.001	1.258	1.356	1.820	1.789	2.456	2.124	2.124	2.640	2.473	2.146
ENSG00000185668	POU3F1	5.302	8.218	2.034	2.599	3.893	1.979	1.223	1.223	0.738	0.225	0.457
ENSG00000141384	TAF4B	4.025	4.427	1.716	1.140	2.422	2.278	2.350	2.350	2.001	3.513	2.810
ENSG00000087303	NID2	0.854	0.644	4.025	10.040	4.641	2.447	4.108	9.108	5.899	4.130	5.903
ENSG00000137090	DMRT1	0.031	0.017	1.023	2.125	1.567	1.823	1.576	1.586	1.401	2.870	1.357
ENSG00000152990	GPR125	0.143	0.269	1.621	1.680	0.390	0.842	1.934	1.702	1.275	1.403	1.866
ENSG00000165731	RET	0.485	1.261	0.073	0.231	0.042	0.048	0.037	0.268	0.719	1.529	0.748
**Gern cell-related genes**												
ENSG00000179598	PLD6	1.184	0.027	16.685	9.617	23.654	12.245	48.294	17.199	9.929	33.417	43.048
ENSG00000125995	ROMO1	3.564	3.362	346.881	411.798	70.450	147.419	26.438	26.084	139.003	116.642	125.376
ENSG00000166147	FBN1	0.948	0.888	11.171	6.547	8.354	5.558	6.996	10.383	88.437	16.180	72.608

ENSG00000151287	TEX30	1.691	1.980	13.111	8.451	9.568	8.401	11.684	10.836	101.555	103.437	194.123
ENSG00000115738	ID2	1.479	1.167	5.071	5.423	6.306	5.377	6.388	5.981	71.332	25.939	41.496
ENSG00000213965	NUDT19	1.393	1.383	14.070	19.544	39.803	21.006	99.780	147.340	10.340	15.400	26.041
ENSG00000179598	PLD6	1.184	0.027	6.685	9.617	23.654	12.245	48.294	17.199	9.929	33.417	43.048
ENSG00000012061	ERCC1	1.134	1.594	33.282	26.934	13.198	15.142	13.131	10.582	20.603	19.563	25.548
ENSG00000101883	MORN2	1.126	0.752	5.803	8.431	5.328	5.753	4.970	6.198	23.551	14.117	21.545
ENSG00000101439	CST3	3.121	2.242	211.654	127.019	37.564	84.369	35.133	59.789	53.415	64.772	36.054
ENSG00000176046	NUPR1	0.070	0.063	8.550	11.825	7.941	5.720	8.994	5.648	15.365	34.077	12.392
ENSG00000278763	FAM27B	0.390	0.319	20.469	26.989	13.325	9.367	12.255	5.182	8.231	7.566	6.823
ENSG00000150783	TCTEX1D2	0.924	0.725	7.390	9.135	5.517	5.005	4.053	8.761	16.481	9.893	24.375
ENSG00000155957	TMBIM4	1.024	0.941	3.571	4.057	3.721	3.635	6.499	6.124	12.109	6.599	8.939
ENSG00000104863	LIN7B	0.412	0.480	7.912	4.602	5.129	4.988	4.850	6.673	5.975	12.696	10.206
ENSG00000119650	IFT43	1.166	1.164	10.464	17.723	3.696	6.183	3.243	3.693	7.873	5.579	10.604
ENSG00000090339	ICAM1	0.650	1.019	6.031	4.773	4.852	3.538	6.955	30.454	10.249	3.733	7.493
ENSG00000120725	SIL1	0.961	0.957	16.180	14.800	11.026	9.544	12.039	8.911	9.163	6.127	6.981
ENSG00000188375	H3F3C	0.314	0.353	17.530	68.575	29.649	24.289	8.763	9.514	6.012	9.308	6.187
ENSG00000108384	RAD51C	0.841	0.440	6.971	6.836	5.424	3.245	7.782	4.236	4.091	4.561	5.244
ENSG00000070404	FSTL3	0.707	0.707	4.715	4.676	2.534	4.302	3.615	5.081	7.771	6.338	5.239
ENSG00000124191	TOX2	0.453	0.647	2.856	5.136	2.935	3.361	4.032	2.910	3.044	2.880	3.298
ENSG00000121350	PYROXD1	0.834	1.003	2.311	3.178	3.433	3.085	2.037	4.391	10.909	9.441	7.260
ENSG00000013297	CLDN11	0.212	0.233	19.763	3.046	10.355	7.024	12.894	6.144	1.392	1.789	2.573
ENSG00000032742	IFT88	0.949	0.934	4.263	3.079	5.170	2.260	4.367	2.502	8.179	9.040	7.595
ENSG00000162039	MEIOB	0.088	1.034	3.491	2.225	3.988	2.912	2.982	4.161	8.013	16.968	20.986
ENSG00000114473	IQCG	0.637	0.660	2.193	2.020	1.937	2.801	1.563	3.320	6.662	9.796	5.213
ENSG00000108384	RAD51C	0.841	0.440	5.971	4.236	5.424	6.836	7.782	4.245	4.091	4.561	5.244
ENSG00000064199	SPA17	0.949	0.903	1.934	2.088	1.664	2.254	2.207	2.853	4.977	7.571	7.282
ENSG00000173559	NABP1	0.103	0.189	1.816	2.373	1.598	2.385	1.677	3.466	9.910	6.622	4.174
ENSG00000140092	FBLN5	0.531	0.520	3.266	2.682	3.214	2.854	5.062	4.161	4.812	3.352	12.438
ENSG00000132141	CCT6B	0.347	0.371	2.521	2.555	2.892	2.810	3.609	3.393	2.545	5.781	6.618
ENSG00000047634	SCML1	0.385	0.151	2.872	2.400	3.360	2.191	3.952	3.486	8.625	20.545	27.773
