# Supplementary figures and images for: Derivation and propagation of spermatogonial stem cells from human pluripotent cells
Source: Stem Cell Res Ther. 2020 Sep 23;11:408. doi: 10.1186/s13287-020-01896-0 (PMC7509941; doi:10.1186/s13287-020-01896-0)

**
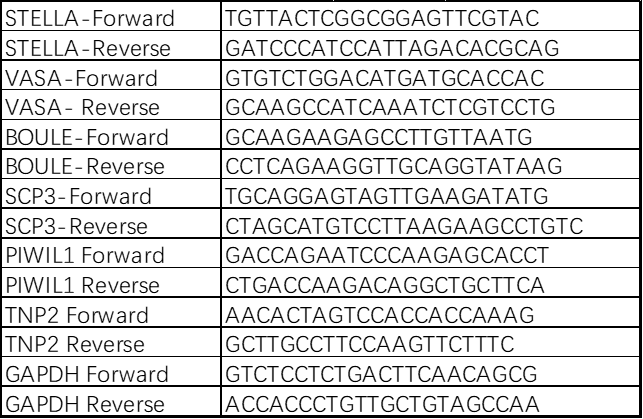
**

Supplement: Supplementary file 1 — Additional file 1: Table S1. Primers of real-time PCR for germ cell markers. Tables S2. List of 1042 transcripts and their normalized FPRM from RNA-seq in the group of hPSCs, SSCLCs and GPR125+ cells isolated from human testes. Related to Fig. 4. Tables S3. List of transcripts related to pluripotency, SSC markers, germ cells and their FPKM from RNA-seq in the group of hPSCs, SSCLCs and GPR125+ cells. Related to Fig. 4 and Figure S2. Table S4. SSCLCs restore recipient testicular spermatogenesis after transplantation at different time points by Johnsen’s Score. Related to Figure S3A. [file 13287_2020_1896_MOESM1_ESM.zip › Table S1_ESM.docx]

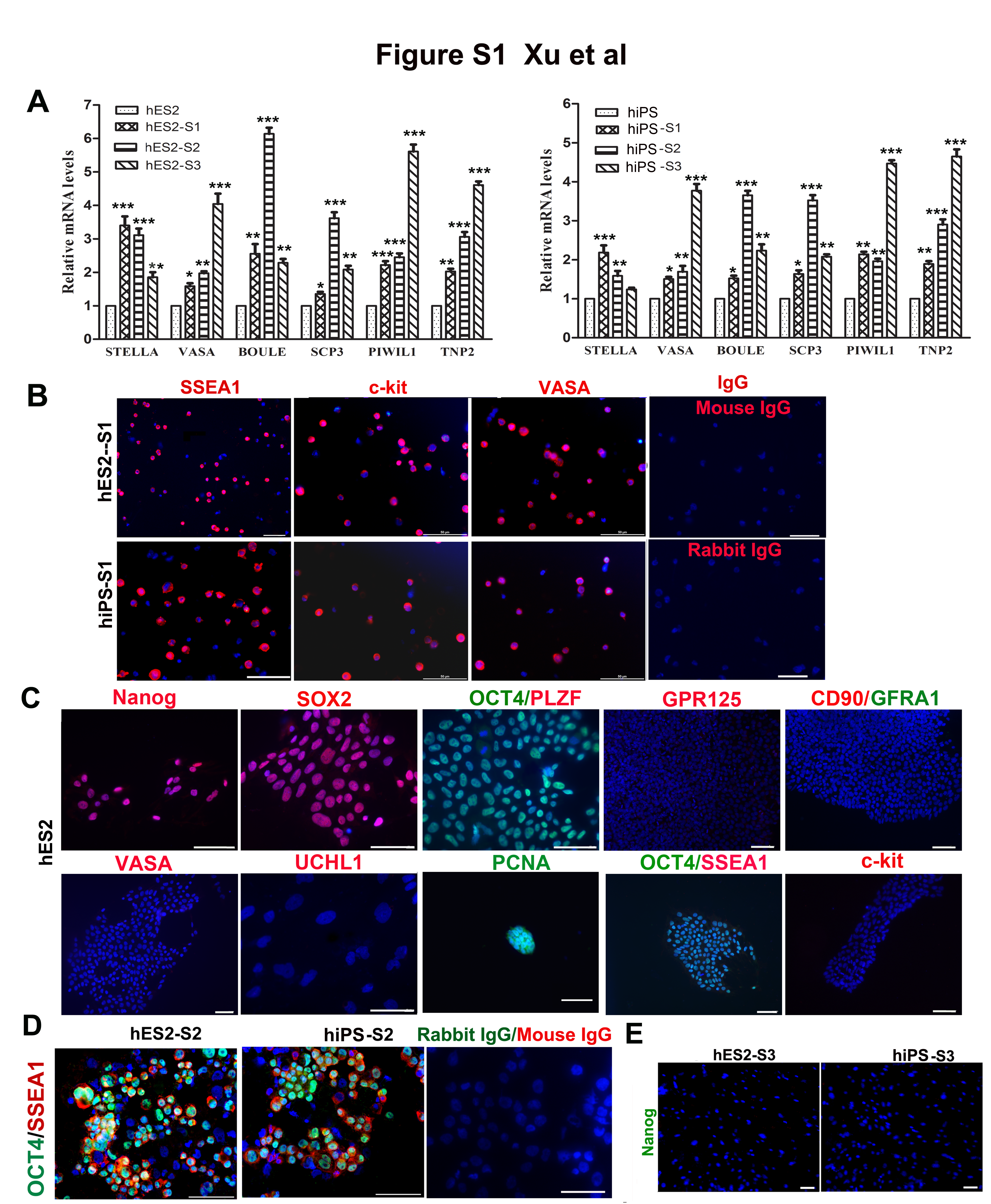

Supplement: Supplementary file 2 — Additional file 2: Figure S1. PGCs, spermatocytes and haploid cells were also generated from hES2 and hiPSC during hPSCs differentiation. a Quantitative real-time PCR of mRNA levels for germ cell marker genes expressed by hES2 and hiPS-differentiated cells at stage1–3. b Immunostaining images of hES2 and hiPS-differentiated cells after 1-step induction with PGC marker antibodies. Mouse and Rabbit IgG were used as negative control. Scale bars: 50 μm. c Immunostaining assay of hES2 with pluripotent markers , SSC markers and PGC markers. Scale bars: 50 μm. d Immunostaining of differentiated cells at stage 2 (S2) with OCT4 and SSEA1. Mouse and Rabbit IgG were used as negative control. Scale bars 50 μm. e Immunostaining of hES2- and hiPS-differentiated cells at S3 with Nanog. Scale bars: 50 μm. [file 13287_2020_1896_MOESM2_ESM.tif]

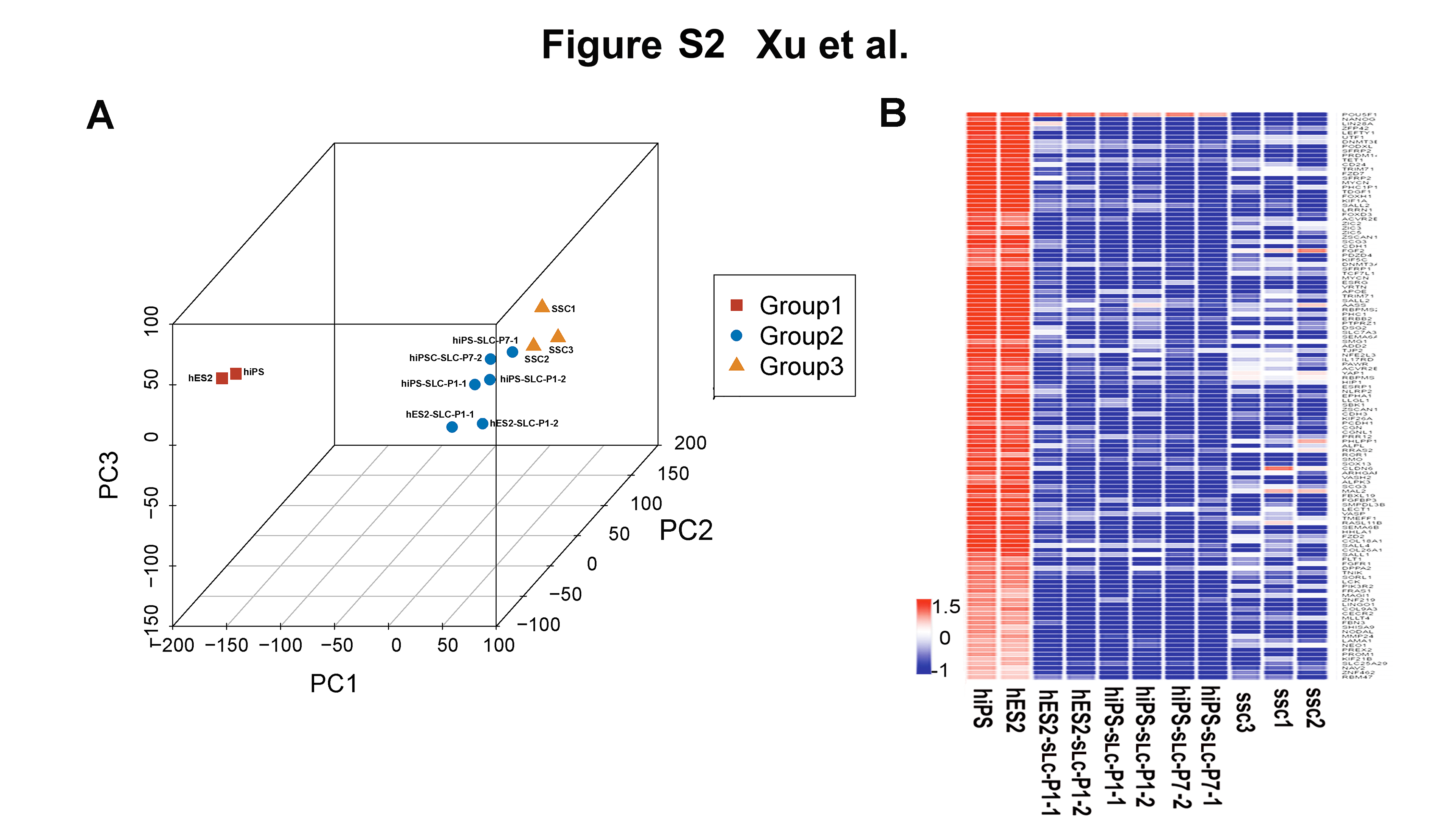

Supplement: Supplementary file 3 — Additional file 3: Figure S2. Transcriptome analyses of hPSCs, SSCLCs and human GPR125+cells isolated from human testes. a PCAon hPSCs, SSCLCs and human GPR125+cells b Heatmap on the transcript expression of pluripotency-related genes in the hES2, hiPS, SSCLCs and human GPR125+ cells. SLC represents SSCLCs. SSC1, SSC2 and SSC3 represent GPR125+ cells. [file 13287_2020_1896_MOESM3_ESM.tif]

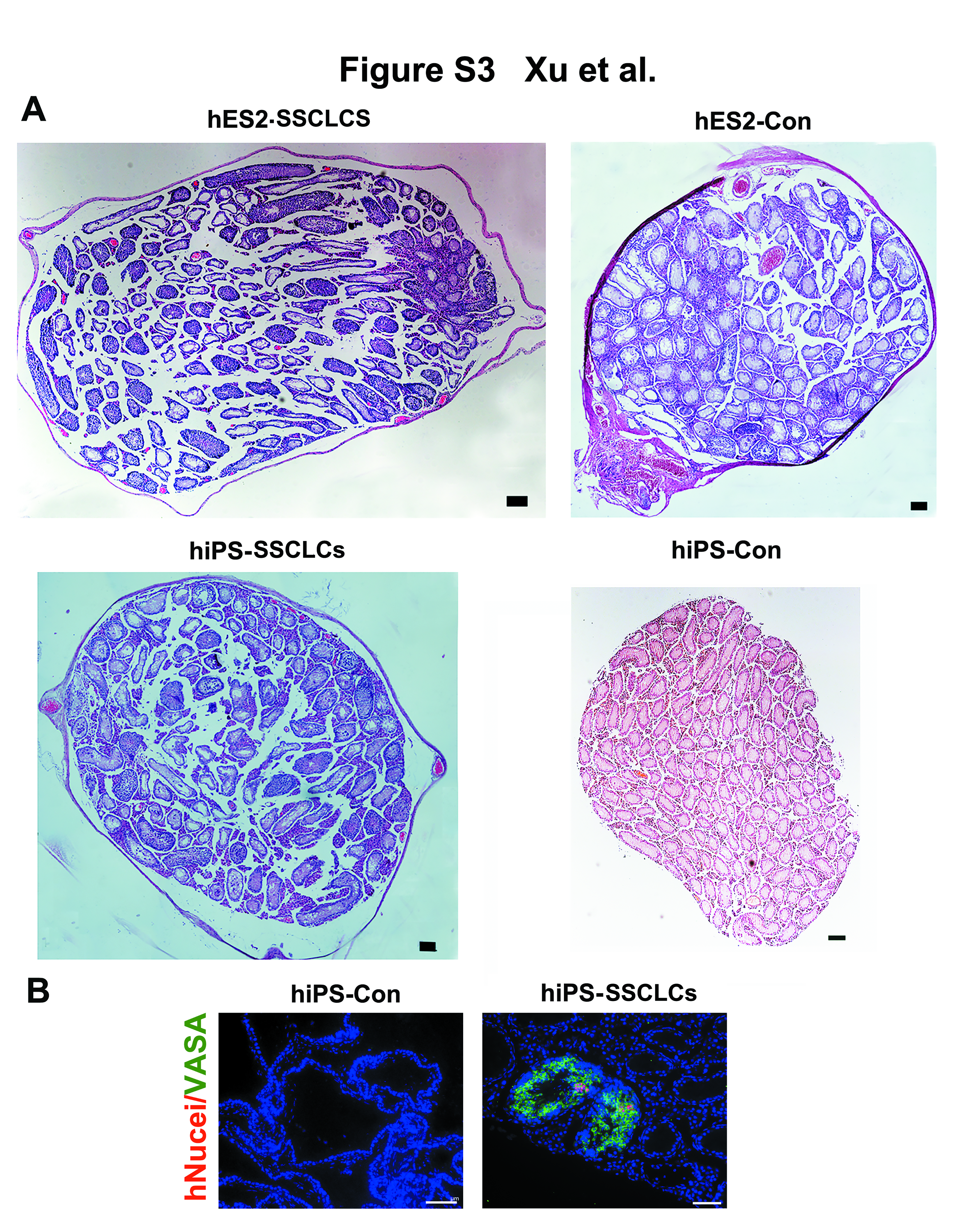

Supplement: Supplementary file 4 — Additional file 4: Figure S3. SSCLCs promote recipient testicular spermatogenesis by H&E staining. a The section of mouse testes at 5 weeks after cell transplantation by H&E staining. Scale bars:100 μm. b Survival of SSCLCs grafts were detected by immunostaining with the antibodies against hNuclei and VASA. Scale bars:50 μm. [file 13287_2020_1896_MOESM4_ESM.tif]

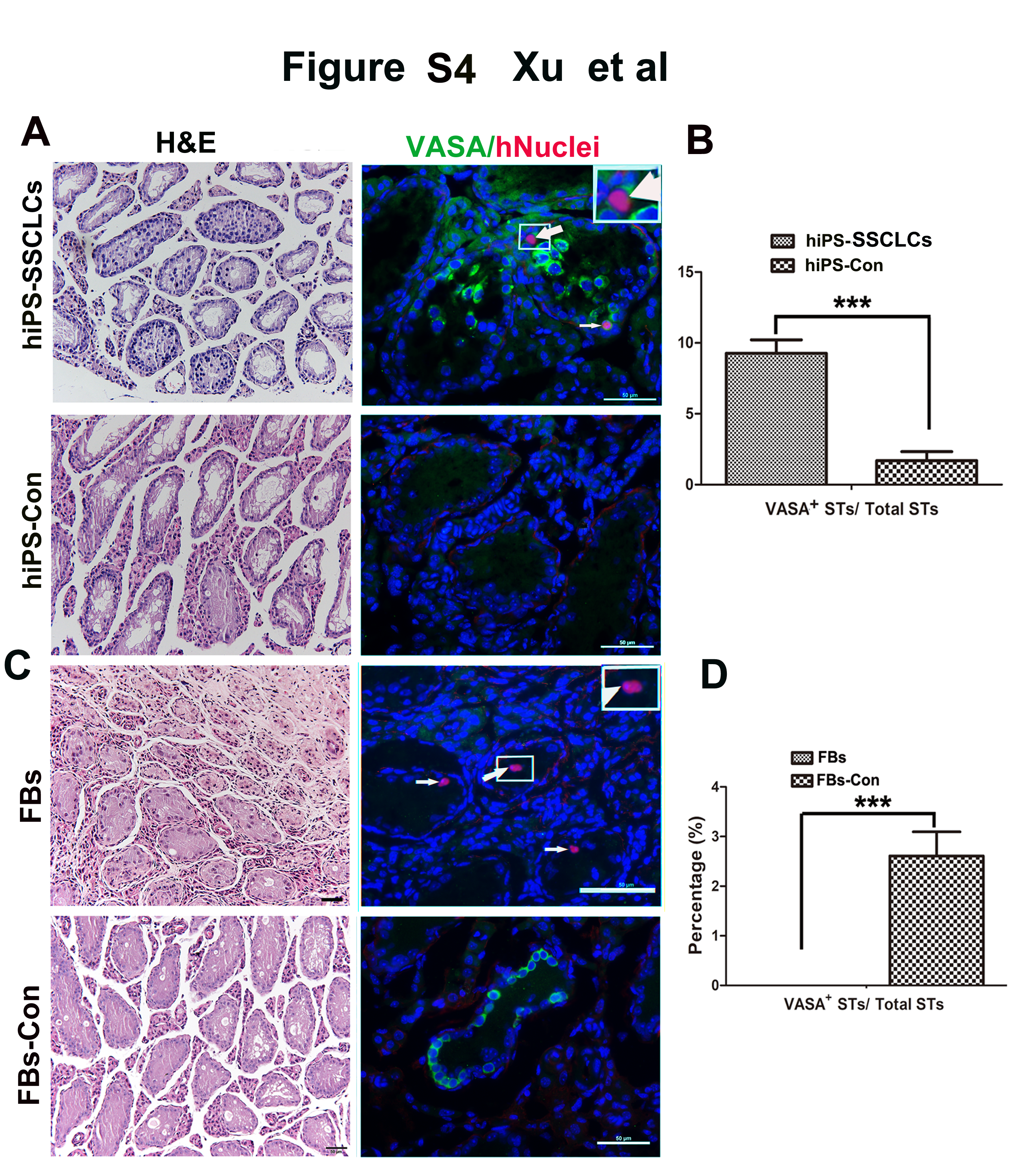

Supplement: Supplementary file 5 — Additional file 5: Figure S4. SSCLCs but not hiPSC-derived fibroblasts promote recipient testicular spermatogenesis. H&E staining and immunostaining with VASA and hNuclei of mouse testes at 5 weeks after cells transplantation. a hiPSC-SSCLCs at P7 promoted recipient mouse testicular spermatogenesis. White arrow represents transplanted SSCLCs. Scale bars: 50 μm. b Quantification of the percentages of seminiferous tubules containing VASA+ cells over the total seminiferous tubules. STs represents seminiferous tubules. c hiPS-derived fibroblast (FBs) at P2 did not promote receipt mouse testicular spermatogenesis. post-transplantation White arrow represents transplanted FBs. Scale bars: 50 μm. d Quantification of the percentages of seminiferous tubules with VASA+ cells over total seminiferous tubules. [file 13287_2020_1896_MOESM5_ESM.tif]
